# Second-order facial features are processed analytically in composite faces

**DOI:** 10.3758/s13414-025-03144-0

**Published:** 2025-08-29

**Authors:** Xue Jun Cheng, Daniel R. Little

**Affiliations:** Melbourne School of Psychological Sciences, The University of Melbourne, Melbourne, Australia

**Keywords:** Face perception, Information processing, Systems factorial technology, Holistic processing

## Abstract

In contrast to claims of holistic processing, upright aligned composite face morphs were recently shown to be processed in the same manner as inverted or misaligned composite face morphs (Cheng et al. 2018. *Journal of Experimental Psychology: Learning, Memory and Cognition, 44*, 833–862). In the present paper, we replicate that work, using a set of schematic faces which vary second-order features (e.g., lip height and eye separation) in the top and bottom halves of the schematic face. We find that the present stimuli show the hallmarks of holistic processing in a complete composite face task, but differ from composite face morphs in that the best fitting MDS metric is more commensurate with an assumption of integrality (i.e., Euclidean distance). Nevertheless, we also find that, as with morph faces, the processing of upright aligned and upright misaligned faces is consistent with a mixture of serial and parallel processing. Importantly, we found little evidence of any strong holistic pooling of the top and bottom face halves into a single object. These results remain consistent with the idea that composite faces are not processed differently from other objects with separable dimensions but instead that composite faces allow more parallel processing when aligned than when misaligned. Data and code are available from: http://github.com/knowlabUnimelb/SCHEMATICFACERULES.

**Supplementary Information:**

The online version contains supplementary material available at 10.3758/s13414-025-03144-0.

## Public significance statement

Faces are often assumed to be treated by the brain as a single object and processed “holistically” (as a whole object). What “holistic” processing actually means though depends on how it is measured. Usually, holistic processing is inferred if upright, aligned faces reveal different patterns of data than other orientations or alignments, but patterns in data do not necessarily reveal anything about the particular way that faces are processed if multiple theories make the same predictions. One popular task is called the composite face task, and in this task different top and bottom faces are shown and people have to detect changes in one half while ignoring the other. We show that upright faces are best explained by assuming that on some trials people process the top and bottom halves together in parallel but on other trials focus attention on one half, processing the top and bottom halves serially. When you split the face, this increases the proportion of serial processing. Notably, we do not find any evidence that faces are treated as holistic objects. Our methods are important because it is notoriously difficult to tell these different types of processes apart. Our results clarify what holistic processing actually means for composite faces.

## Second-order facial features are processed analytically in composite faces

Faces are thought to be processed differently from other objects. Face perception is sub-served by a distinct neural region (Kanwisher et al., 1997; O’Craven & Kanwisher, 2000), and the ”specialness” of faces has been demonstrated by multiple studies which show that faces are susceptible to manipulation in a way that other objects are not. For instance, face recognition is impaired by inversion, but cars, houses, and dogs are not (Scapinello and Yarmey, 1970; Tanaka and Farah, 1993b; but see Cornes et al., 2011; Wenger and Ingvalson, 2003). These results can be attributed either to the intrinsic holistic nature of face processing or to our extensive expertise in viewing faces and using face information for identification and recognition (Diamond & Carey, 1986; Gauthier & Tarr, 1997). Our unique experiences with viewing faces may lead to the development of face templates or mandatory attentional strategies (Kanwisher et al., 1997; Gauthier et al., 1999) that result in differences in how faces are processed or represented; such changes in representation for other types of stimuli are rare (though see Garrett et al., 2022).

Despite a wealth of research, there are ambiguities in how holistic processing is measured and defined. One factor is the use of accuracy measurements (Rossion & Boremanse, 2008; Young et al., 1987). Many theoretical models can predict decrements in accuracy in the task conditions used to examine face processing; this *model mimicry* issue is pervasive. As decisions require the accumulation and combination of information from multiple sources in real time, the analysis of response times (RTs) can provide a more detailed definition of holistic processing and allow different theories of holistic processing to be differentiated. In the present paper, we adopt an RT approach based on Systems Factorial Technology (SFT; Fifić and Little, 2017; Little et al., 2017; Townsend and Nozawa, 1995; Townsend and Wenger, 2004). As described in Altieri (2017), SFT can be used to identify whether information contained in facial features (e.g., eyes, nose, mouth) is pooled into a single holistic representation or processed independently in serial or parallel, along with other fundamental characteristics of information processing.

In detail, SFT provides a methodology for inferring the architecture or organization of information processing (e.g., whether multiple sources of information are processed independently, in serial or in parallel, or pooled into a single coactive decision; Kantowitz, 1974; Sternberg, 1969; Schweickert, 1993; Townsend, 1984), decision stopping rules (e.g., whether processing is exhaustive or self-terminating), the independence of processing channels (e.g., whether there is any "cross-talk" between processing channels), and the efficiency of processing (workload capacity, or simply, capacity). SFT has now been applied to answer questions about, among others, how targets and distractors are processed in visual search (Fifić et al., 2008b), how items are accessed in memory during recognition (Townsend & Wenger, 2004) and semantic search (Shang et al., 2021), speed-accuracy trade-offs (Donkin et al., 2014), the effects of cuing on visual attention and detection (Yang et al., 2014, 2018, 2019; Chang et al., 2016; Howard et al., 2021), change detection (Blunden et al., 2022; Fong et al., 2023), and, most important for the present investigation, how features are processed during perceptual categorization (Blunden et al., 2015, 2020; Fifić et al., 2010a; Little et al., 2011, 2013; Moneer et al., 2016).

In the following sections, we review definitions of holistic processing and illustrate the composite face task in detail. We then describe the link between information processing models and the composite face task. As outlined by Wenger and Townsend (2001), combinations of processing architecture and stopping rule can be placed on a continuous spectrum from strong holism (e.g., pooled coactive processing) to weak holism (e.g., independent parallel processing or exhaustive processing). We review how SFT can be used to tell apart these different processing architectures and stopping rules.

Our primary question concerns the processing of second-order facial features; that is, the distance between facial features (eye-separation and lip-to-nose height) organised into *schematic* composite faces. Hence, we first confirm that our schematic faces demonstrate a composite face effect (Experiment 1). We then apply a double-factorial SFT task (Experiment 2) to show that despite showing a configural face effect and despite showing evidence of integrality, we do not find any strong evidence of coactivity - or in other words, we do not find any evidence for the strongest form of holistic processing. We further draw a contrast between our prior work (Cheng et al., 2018) and the present work. Whereas prior multidimensional scaling results using face *morph* composites were best described using city-block distance, the schematic composites are better explained by a Euclidean metric (Experiment 3). Finally, our computational modelling anlayses show the preferred model for Experiment 2 is one which combines a mixture of serial and parallel processing.

### Definitions of holistic processing

Holistic processing is frequently associated with the idea that facial parts are integrated into a unified whole, consistent with Gestalt principles. Such a perspective suggests faces are processed as complete templates, where individual parts and their configuration are amalgamated into a singular object. Another perspective defines holism as the recognition benefit derived from the specific configuration of an upright face (Diamond & Carey, 1986; Rhodes et al., 1993; Searcy & Bartlett, 1996; Leder & Bruce, 2000), which allows for an attention strategy (learned through our extensive experience with faces) to be automatically deployed for identification and recognition (Richler et al., 2011b, a; Wong & Gauthier, 2010). This latter definition has been most directly explored using the composite face task, which is used to demonstrate that an automatic attentional strategy cannot be “turned off” when faces are aligned leading to facilitation and interference effects (on d-prime) compared to when faces are misaligned.

### Composite face task

In the composite face task, participants must ignore a non-target face half while detecting changes in the target half (Hole, 1994; Young et al., 1987). In a variation known as the *partial* composite design, the target half of the test face is paired with a non-target half from a different face (Goffaux & Rossion, 2007; Rossion & Boremanse, 2008). Faces in aligned and misaligned configurations are compared, and holistic processing is inferred from higher accuracy for “same” decisions with misaligned faces. The assumption is that misalignment helps the observer ignore the interfering non-target half. Rossion’s (2008) preferred interpretation of this effect is that the composite face forms a perceptual illusion when aligned. However, Gauthier and Bukach (2007) highlighted that accuracy, as the dependent variable, may be influenced by response biases independently of sensitivity changes with alignment (see e.g., Green and Swets, 1966; Macmillan and Creelman, 2005).

To accurately assess response bias and sensitivity, Gauthier and Bukach (2007) outline that a *complete* composite design is necessary. In the complete design, first used in Farah et al. (1998), both the target and non-target face halves can either match or mismatch the study face, resulting in congruent (both halves the same or both halves different) and incongruent trials (target same/non-target different or target different/non-target same). Holistic processing is inferred from a congruency $\times$ alignment interaction in sensitivity (e.g., d-prime, Richler et al., 2015).

The explanation for the congruency $\times$ alignment interaction effect is straightforward: misalignment allows attention to focus on the target half, reducing interference from the non-target half and maintaining roughly equivalent d-prime between congruent and incongruent conditions. In aligned faces, incongruency leads to interference from the non-target half, decreasing d-prime, while congruency enhances detection, increasing d-prime. In essence, if holistic processing and subsequent interference from the non-target face half occur (indicating a failure of selective attention; Richler and Gauthier, 2013), the complete composite face task should exhibit an interaction effect. The interaction has been observed for both upright and inverted faces, but the effect is larger for upright faces (Cheng et al., 2018; Cheung et al., 2008; Chua et al., 2014, 2015; Richler et al., 2011a, 2015).

### Linking the composite face task and the logical-rules task

Regardless of whether the partial or complete composite design is used, neither reveals how the face halves are processed. At issue is the fact that the congruency $\times$ alignment interaction can be explained by a number of different processing models.

Wenger and Townsend (2001) outlined that the elements of information processing exist on a continuum that can be organised from less holistic to more holistic. To briefly summarise, serial processing can be thought of as less holistic than parallel processing, and self-terminating processing can be thought of as less holistic than exhaustive processing. The kind of analytic processing which is often contrasted with holism is often characterized as serial and self-terminating (Tversky, 1969; Hole, 1994; Fitousi et al., 2021). Likewise, terminating processing after processing a target feature ensures that there is little interference from non-target features (Fifić & Townsend, 2010; Yang et al., 2018).

Interference can arise from an interaction between channels (Eidels et al., 2011; Townsend & Wenger, 2004); consequently Wenger and Townsend (2001) specify that independent processing is less holistic than interactive processing. Holism is variously defined as an interaction that arises from cross-talk between parallel channels or face templates, which is most closely aligned with pooling into a single channel (i.e., coactivity).

The goal of the composite face task is simply to assert that the congruency $\times$ alignment interaction exists (or not) and, if it does exist, then some form of holistic processing, however defined, must be at play. However, the composite face task is mute with regard to which combination of properties appropriately accounts for processing. To move beyond effect-based or phenomenon-based research (Meehl, 1967; Smith & Little, 2018), we need to take seriously the issues of model mimicry (Townsend, 1990a). This requires the methodological tools offered by Systems Factorial Technology, tools that allow for identification of the relevant aspects of information processing (Townsend & Nozawa, 1995; Townsend & Wenger, 2004; Little et al., 2017). We draw most prominently on the categorization version of the double factorial task introduced by Fifić et al. (2008a).

**Fig. 1:**
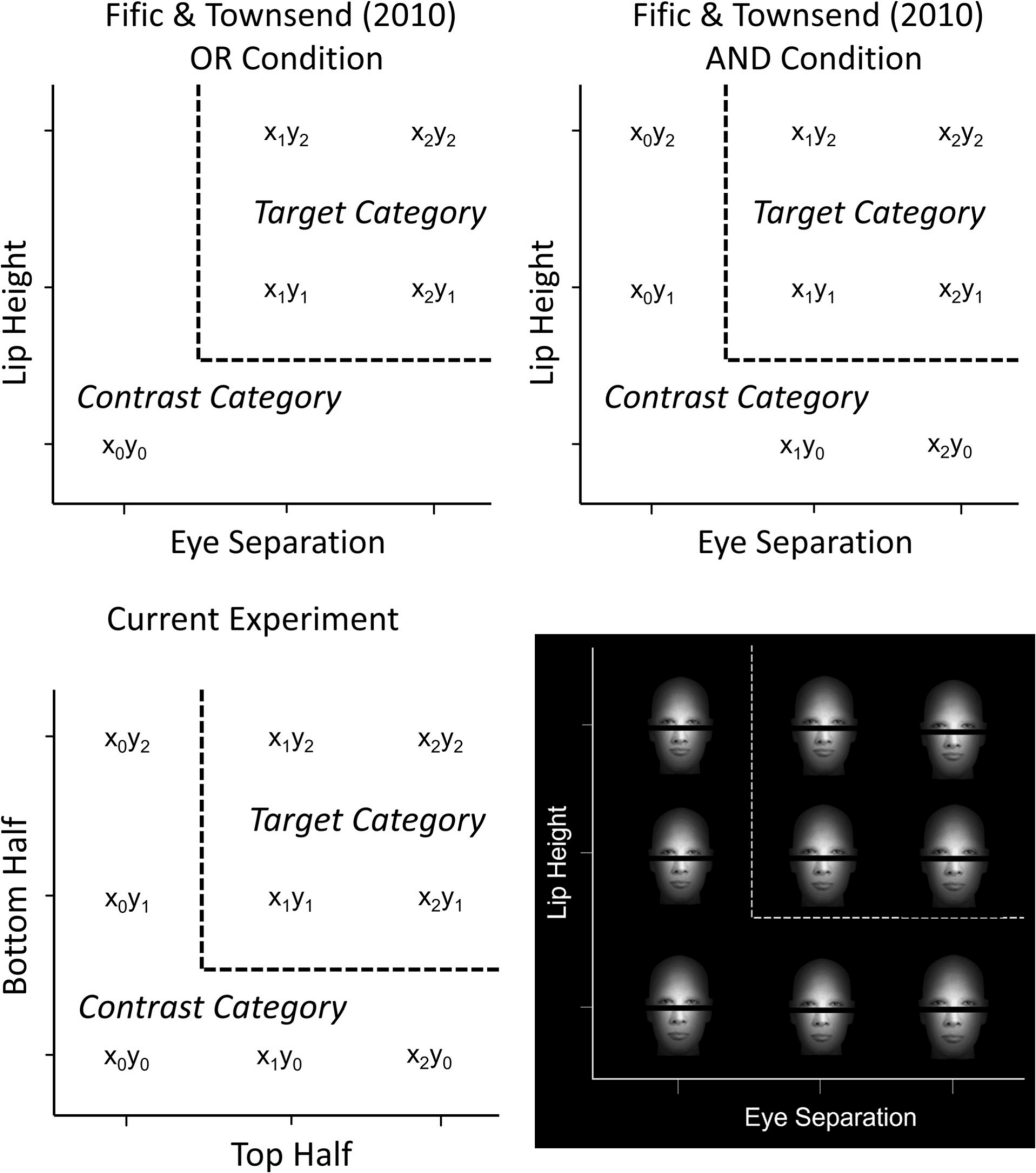
Top left and top right show the schematics of the stimulus spaces in Fifić and Townsend (2010)’s OR and AND conditions. Bottom left shows the current experiment space and bottom right shows the combination of faces used in the upright aligned face category space in the current experiment based on the dimensions of eye separation and lip height

Fifić et al. (2008a) introduced a categorization task in which the participant’s goal was to make a decision about whether the values of specified stimulus dimensions satisfied certain rules (e.g., a conjunction rule; see also Fifić et al., 2010a; Fifić and Townsend, 2010). Several SFT analyses (described further below) can be applied to RTs from decisions about faces from this task to make inferences about holistic processing. The category space used in these experiments is shown in Fig. 1.

As detailed in Griffiths et al. (2017) and Cheng et al. (2017), this category space has been used successfully to uncover detailed information about the architecture underlying the processing of different types of stimulus dimensions. For instance, Little et al. (2013) contrasted integral and separable dimensions (Garner, 1974) showing that the former (squares comprised of different values of brightness and saturation) were processed coactively but the latter were processed in an independent manner, either in serial or in parallel. This is a particularly relevant example because integral dimensions are difficult to attend to in isolation and exhibit similar properties to what we might expect from holistic processing. Many different types of feature combinations have now been explored using this paradigm (Cheng et al., 2018; Fifić et al., 2008a, 2010a; Little et al., 2011, 2013; Blunden et al., 2015; Moneer et al., 2016; Blunden et al., 2020, 2022; Fong et al., 2023), and a natural question is whether this paradigm can be extended to investigate how composite faces are processed.

Cheng et al. (2018) investigated the architecture underlying *face morph* stimuli in an experiment similar to the one reported here and found that processing of the top and bottom half of a composite face were most consistent with the independent processing of each half. More specifically, on some trials, the top and bottom half were processed in serial but on other trials in parallel. Critically, no evidence of strong holistic processing, defined here as a pooling of the top and bottom half into a single coactive processing channel, was found. A model which assumed a trial-by-trial mixture of serial and parallel processing provided the best quantitative account of the data; see Little et al. (2019) for details on the SFT properties of these models. The key takeaway message is that there was little evidence of strong forms of holistic processing during categorization (i.e., coactivity) with these morphed composite faces despite confirming that the same stimuli satisfied other results consistent with holistic processing (e.g., the congruency $\times$ alignment interaction expected in the complete composite face task).

Fifić and Townsend (2010) used schematic face stimuli in a similar SFT categorization paradigm to investigate changes in processing architecture based on manipulations of two second-order relational facial features of eye separation and lip position that varied in feature saliency (i.e., high or low discriminability). These second-order relational features involve spatial relationships between the facial features (e.g., how far apart they are) instead of focusing on the type of features themselves (i.e., a specific nose; Diamond and Carey, 1986) and are considered important in face perception (Leder & Bruce, 2000; Maurer et al., 2002; Rhodes, 1988; Searcy & Bartlett, 1996; Sergent, 1984). Unlike face morphs, schematic faces do contain specific featural information that can be analyzed to form the basis of a response.

During the learning phase of Fifić and Townsend’s (2010) study, participants were assigned to either the OR condition, where it was possible to correctly categorize a face based on only one dimension, or the AND condition, where both dimensions are required. The key manipulation occurred at test, where the same second-order features could be presented either in the same context (i.e., the same background head shape and nose) as used during training, a new context, or as isolated parts without a head. Participants were tested on all three conditions: the old-face context, new-face context, and features-alone context within the same logical rule condition (AND or OR) that they were assigned in the learning phase. In the new-face context, novel configurations of faces were mixed in with old faces used in the learning phase, and participants were reminded that recognition of the lip position and eye separation was still necessary and sufficient to generate a correct response. In the features-alone context, novel configurations were mixed in with the old faces again except some faces would be presented with just their eyes and lips visible, without the entire face context. Finally, in the old-face context, only faces from the learning phase were presented. This old-face context is the most similar to the methods we use in our present paper (our Experiment 2) as participants had to make a decision about which of two categories the displayed face belonged to. The results revealed mainly parallel self-terminating processing in the OR condition, with participants being able to stop processing as soon as either channel had completed processing. In contrast, and unlike Cheng et al. (2018), participants in the AND condition showed some evidence for coactive processing.

Finally, in a related study using second-order features, Yang et al. (2018) used SFT to investigate the other-race face effect. The other-race face effect is the finding that discrimination of faces from people belonging to a race other than one’s own is slower and may occur more analytically than faces belonging to one’s own racial in group. Using an OR task similar to (Fifić and Townsend, 2010, see Fig. 1, top left panel), they found that second-order features of own-race faces (eye separation and lip height) were processed in parallel, which might be considered a weak form of holistic processing (see Wenger and Townsend, 2001). By contrast, other-race faces were processed in a serial self-terminating fashion.

These results, in contrast to Cheng et al. (2018), indicate that second-order features may be processed differently than face morphs. In the present study, we extended our investigation of face processing to schematic faces similar to Fifić and Townsend’s (2010) using the paradigm developed in Cheng et al. (2018). Unlike the morphed faces, schematic faces are made up of fixed values that represent the differences between the space between the eyes and the space between the nose and the lips. Unlike Fifić and Townsend (2010), we combine the second-order features of eye separation and lip height into a composite face. We again employ SFT in order to determine whether there is any evidence for holistic processing. There are many good tutorial reviews of SFT (Algom et al., 2015; Altieri et al., 2017; Harding et al., 2016) so we will focus on a summary of the relevant aspects of SFT for the present analyses. We direct the reader to the original paper (Townsend & Nozawa, 1995) or the published volume (Little et al., 2017) for more detailed information.

### Systems factorial technology (SFT)

Our study focuses on two measures: the Mean Interaction Contrast (MIC) and Survivor Interaction Contrast (SIC). Taken together, these analyses can distinguish between the processing architectures by exploiting a property of the design of our category space (see Fig. 1, top right panel). The four members of the target category ($x_1y_1$, $x_1y_2$, $x_2y_1$, and $x_2y_2$) vary according to their discriminability relative to the category boundary on both dimensions. The stimuli closer to the category boundaries should be harder to distinguish from the contrast category and are thus described as having low discriminability (L). Stimuli further from the category boundaries should be easier to distinguish and are consequently described as having high discriminability (H). In Fig. 1, we can see that stimulus $x_1y_1$ is close to both category boundaries and will hence be referred to as the Low-Low (LL) stimulus. Correspondingly, $x_1y_2$ will be referred to as the Low-High (LH), $x_2y_1$ as the High-Low (HL), and $x_2y_2$ as the High-High (HH) stimulus. The MIC and SIC can be computed by combining these factorially-manipulated dimensions both at the mean and distributional level and provide a powerful tool for differentiating processing models. A major benefit of SFT is that it avoids model mimicry issues that impact many popular experimental designs (Townsend, 1990a; Little et al., 2017).

### Mean interaction contrast (MIC)

The target category requires both dimensions (i.e., eye separation and lip height; see Fig. 1) to be processed; hence, they should be categorized using a conjunctive AND rule. Consequently, in this study, we focus only on the exhaustive MIC patterns as self-termination in the target category would result in a high error rate. The mean RT interaction contrast can be summarized as:

1 $$\begin{aligned} MIC = (\overline{RT}_{LL} - \overline{RT}_{LH}) - (\overline{RT}_{HL} - \overline{RT}_{HH}) \end{aligned}$$

where $\overline{RT}_{LL}$ is the mean or expected RT for item condition LL. A full description of these predictions is provided elsewhere (Fifić et al., 2010a). We simply note here that each model makes unique predictions for the mean RTs and the resulting mean interaction contrast. Serial exhaustive models predict an MIC equal to 0. Parallel exhaustive models predict that the MIC should be less than 0. Coactive models predict that the MIC should be greater than 0.^1^

**Fig. 2:**
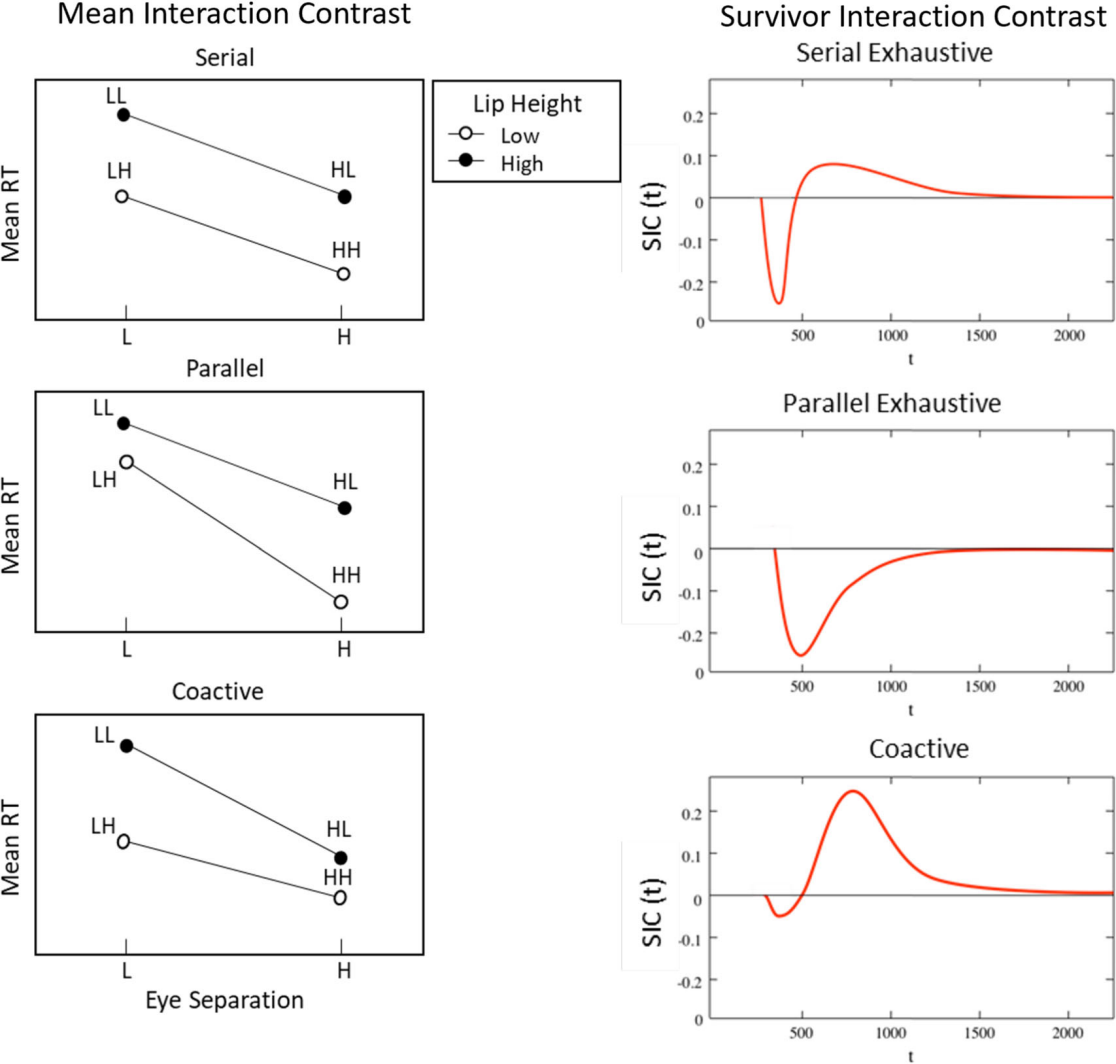
Left panels: Illustration of the mean RT patterns for the target category items in the serial, parallel, and coactive processing architectures. L denotes low discriminability on a dimension; H denotes high discriminability on a dimension. Right panels: Illustration of the survivor interaction contrasts (SICs) associated with the serial, parallel, and coactive processing architectures

### Survivor interaction contrast (SIC)

The survivor interaction contrast (SIC) allows us to gain further information by analyzing the entire RT distribution for each target category item. The SIC is computed analogously to the MIC but uses the survivor function, S(t), for each item condition. The survivor function is defined as the probability that a randomly observed time *T* takes longer than time *t*; $S(t) = P(T>t)$.

2 $$\begin{aligned} SIC(t) = [S_{LL}(t) - S_{LH}(t)] - [S_{HL}(t) - S_{HH}(t)] \end{aligned}$$

As with the MIC, the SIC produces qualitatively distinct patterns for each of the processing architectures (see Fig. 2, right panel). Equal positive and negative area under the curve indicates a serial processing architecture, and an entirely negative curve indicates a parallel processing architecture. Coactive architectures are characterized by a small negative deflection that then becomes mostly positive. As the integral of the SIC gives the value of the MIC (Townsend, 1990b), the MIC is therefore a measure of the sum of the positive and negative area of the SIC.

### Contrast category predictions

The mean RTs from the contrast category items also provide information on distinguishing between the processing architectures and stopping rules via an additional set of qualitative contrasts (Fifić et al., 2010a; Little et al., 2015, 2018). RT patterns from the contrast category items, which follow a disjunctive rule, can distinguish between exhaustive or self-terminating rule for the serial and parallel models.

Following Fifić et al. (2010a), we term the items in the contrast category as *interior* (e.g., $x_1y_0$) or *exterior* (e.g., $x_2y_0$) stimuli or *redundant* ($x_0y_0$; see Fig. 1). Interior refers to the stimuli closer to the dimension boundary, and exterior refers to stimuli further from the dimension boundary. Lastly, the redundant stimulus satisfies the disjunctive rule on both of the dimensions (Fifić et al., 2010a).

**Fig. 3:**
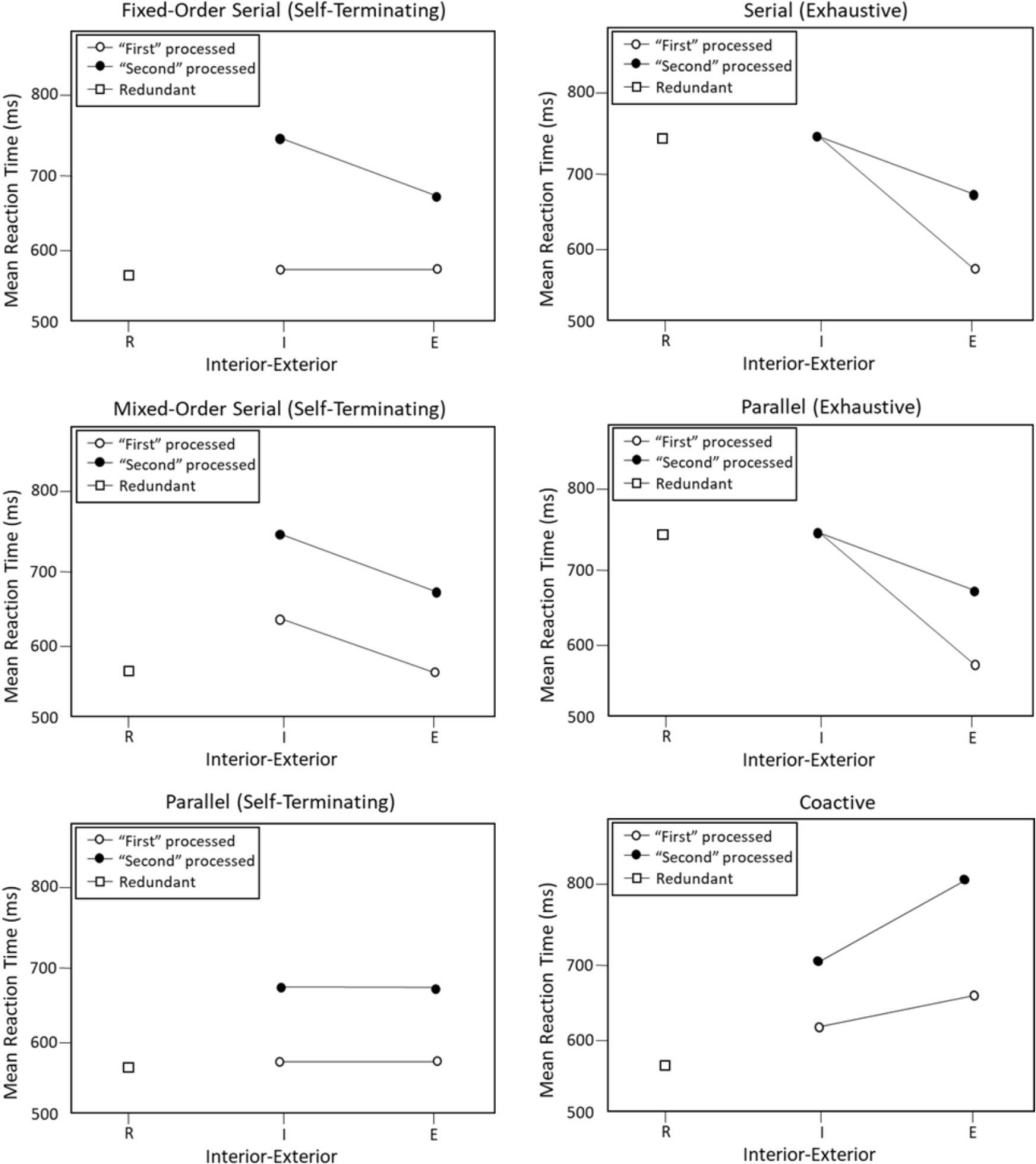
Schematic illustration of contrast category mean RT patterns adapted from Fifić, Little & Nosofsky (2010). R = redundant, I = interior, E = exterior

In a fixed-order serial self-terminating process, one dimension is processed before the other dimension; in this case, the mean RTs for the first processed dimension will be approximately equivalent for both interior and exterior stimuli (see Fig. 3, top left panel). For the second processed dimension, the mean RTs for the interior stimulus (e.g., $x_1y_0$) will be slower than the mean RTs for the exterior stimulus (e.g., $x_2y_0$) since it is harder to discriminate $x_1$ from $x_0$ than it is to discriminate $x_2$ from $x_0$. In a mixed-order serial self-terminating processing (see Fig. 3, middle left panel) either dimension may be processed first. Depending on which dimension is first, processing will have to switch to the other dimension; consequently, the interior stimuli will take longer for the second processed dimension than the exterior stimuli.

For a parallel processing model, processing can also be exhaustive or self-terminating. With a self-terminating stopping rule (see Fig. 3, bottom left panel), decisions along both dimensions are processed simultaneously. Hence, the parallel self-terminating model predicts identical RT distributions for the interior and exterior stimuli within each dimension. It also predicts that the redundant stimulus $x_0y_0$ will be processed fastest as there are more opportunities for processing to self-terminate, leading to a faster minimum RT. In contrast, for parallel exhaustive processing (see Fig. 3, middle right panel), both dimensions are processed so the total time is the maximum of each individual-dimension. This means that the redundant stimulus and interior stimuli are predicted to be processed slower as they lie near both the *x* and *y* decision bounds.

For coactive processing (see Fig. 3, bottom right panel), the interior stimuli are predicted to be faster than the exterior stimuli instead. In coactive processing, we expect a pooling of information across both dimensions; as the interior stimuli are closer to the lower left-hand corner of the stimulus space (i.e., the contrast category), it is expected that they provide more evidence for a contrast category compared to the exterior stimuli. Consequently, the interior stimulus should result in faster categorization decisions compared to the exterior stimuli under coactive processing.

In combination, these non-parametric predictions provide information that allows us to distinguish between different processing architectures, stopping rules, and which order the dimensions are processed in serial processing. More detailed descriptions of the qualitative predictions and their rationale can be found in Fifić et al. (2010a, pp. 313–317).

### Behavioral experiments

The aim of our paper is to investigate the architecture underlying the processing of second-order features in schematic composite faces. In separate conditions, we focus on the processing of aligned and misaligned schematic composite faces. If these composite faces are processed holistically when aligned but not when misaligned, our results should show a different type of responding (e.g., coactive processing) for aligned faces but independent (serial or parallel) processing for misaligned faces. This result would be consistent with Fifić and Townsend’s (2010) findings that suggest coactivity with schematic faces and the existing literature that suggest misaligned faces are not processed holistically.^2^

Before presenting our SFT experiment, we first sought to confirm that our schematic face stimuli demonstrated the hallmark composite face interaction by conducting an experiment using the complete composite face task (Richler & Gauthier, 2013). We followed the procedure used in Richler et al. (2009) and Cheng et al. (2018) with appropriate modifications (reported below).

## Experiment 1: Composite face task method

### Participants

Forty participants were recruited from the Melbourne School of Psychological Sciences’ Research Experience Program (27 F, 13 M, Ages 18-33). Due to computer error, data from four participants was lost leaving 36 participants. Each participant took approximately 45 minutes and received 1 course credit for completion. Testing was approved under the Melbourne Human Research Ethics Committee 1034866. Data were collected in 2015.

A meta-analysis of the complete composite face task recommends at least 13 participants in order to have 95% power to detect the two-way congruency $\times$ alignment interaction (Richler & Gauthier, 2014). We note that, as pointed out by Ross et al. (2015), the reliability of the composite face task is rather low. This reliability is improved by decreasing the number of top and bottom face halves used in the study. Ross et al. (2015) used 5 different top and bottom halves in their Experiment 5, which demonstrated the highest reliability. As explained below, we use two different top and bottom face halves.

### Stimuli and apparatus

Four faces were used combining two levels of eye separation with two levels of lip height. Feature values were chosen based on a preliminary study to find the threshold (75%) separating narrow and wide eye separation and high and low lip height (see Threshold Study for details). Eye separations (105 pixels and 115 pixels subtending an angle of 1.22 degrees and 1.34 degrees, respectively) were chosen to appear “wide” (> 100 pixel separation). Likewise, the distance between the lips and the nose (0 and 8 pixels) was chosen so the lips appeared “high” on the face (< 313 pixels from the top of the head). Eyes and lips were then presented on a 340 width $\times$ 400 height face blank (see Fig. 1). The entire face was presented on a black background with a 5 pixel black bar separating the top and bottom faces halves.

The stimuli were presented on a 1280 $\times$ 1024 resolution CRT monitor with a refresh rate of 100 Hz at a viewing distance of 70 cm. This meant each aligned face subtended a visual angle of 4.65 degrees $\times$ 3.85 degrees and each misaligned face subtended a visual angle of 4.65 degrees $\times$ 3.96 degrees. The black bar separating the face halves subtended a visual angle of 0.11 degrees for all face conditions. RTs were collected using a calibrated response box (Li et al., 2010).

**Fig. 4:**
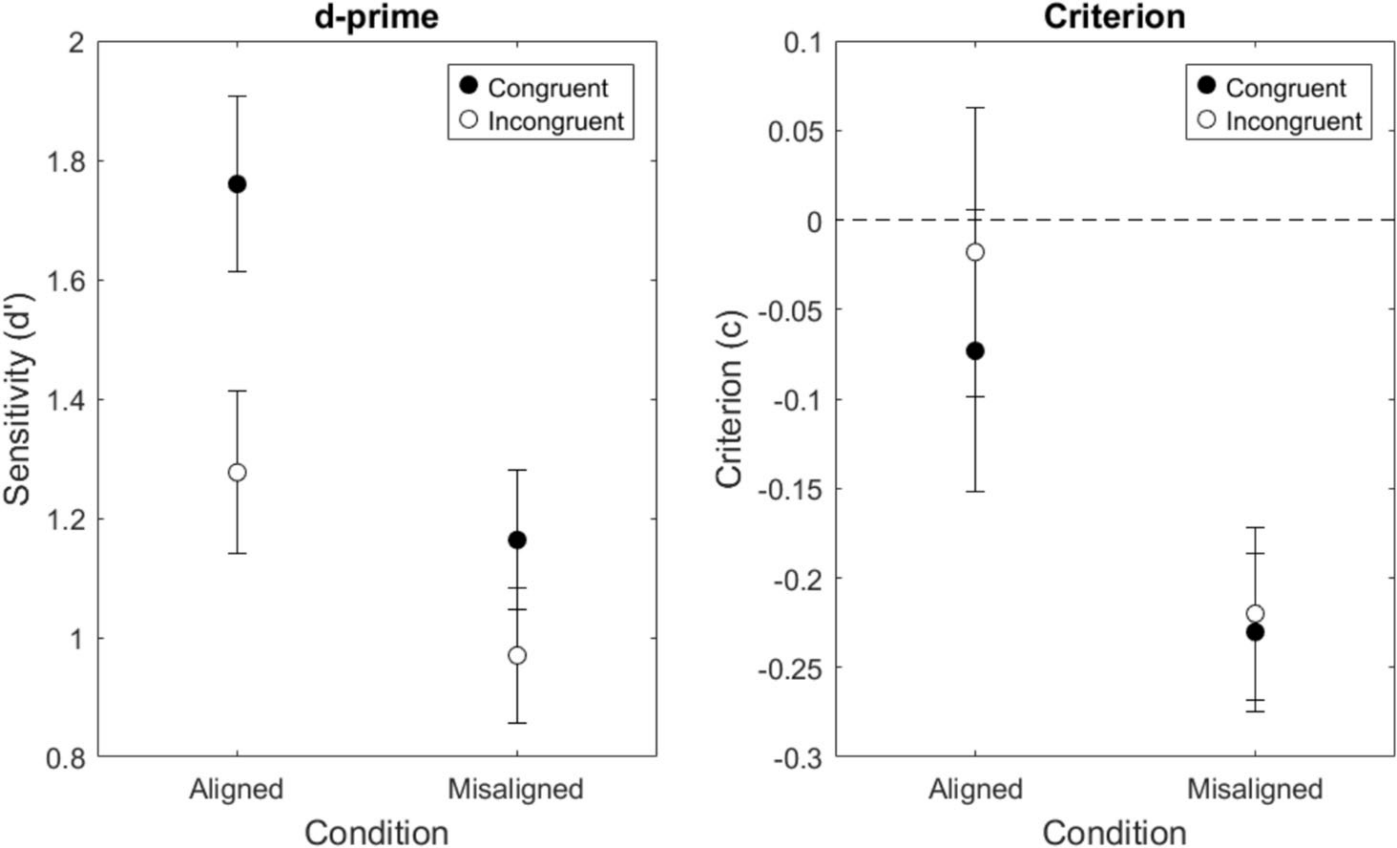
Sensitivity and criterion results (N=36) for Aligned and Misaligned Schematic Face conditions. Error bars represent standard errors

### Procedure

Each participant completed one session of testing that consisted of 16 practice trials and 16 blocks of 32 experimental trials each, making a total of 512 experimental trials. Participants were instructed to respond as accurately and quickly as possible.

At the beginning of each block, participants were asked to attend to either the top or bottom face half with the location of the cued half remaining the same throughout that block (e.g., participants were always cued to attend to the top half in block 1). On each trial, a fixation cross was first presented for 1000 ms. A study face was then presented for 200 ms, followed by the test face also for 200 ms. The participant was then required to indicate whether they thought the two faces were the same or different by pressing the appropriate button on the response box. Feedback was provided at the end of each trial if the response was incorrect (i.e., “...Wrong...”) or if the trial timed out at 5000 ms (i.e., “Too Slow!”). A blank interval of 2000 ms was included between trials. The study face was always upright and aligned, whereas the test face could be either upright or inverted, and aligned or misaligned. Test face direction (upright or inverted) and alignment was randomized on each trial such that all combinations of study and test faces were presented in the experiment. The results for inverted faces are reported in our supplementary material.

### Data analysis

To investigate the interaction between congruency and alignment, we used a 2 Congruency (Congruent vs Incongruent) $\times$ 2 Alignment (Aligned vs Misaligned) Repeated Measures ANOVA separately for the upright and inverted faces.

### Transparency and openness

We have reported how we determined our sample size, all data exclusions, all manipulations, and all measures in the study. All data, analysis code, and research materials are available at https://github.com/knowlabUnimelb/SCHEMATICFACERULES. Experimental code is available here: https://github.com/knowlabUnimelb/FACECOMPRULES. Data were analyzed using custom functions written in Matlab. This study’s design and its analysis were not pre-registered.

## Results

### Composite face task

The d-prime and criterion results are shown in Fig. 4. D-prime was higher in the aligned conditions than in the misaligned conditions and higher when changes were congruent compared to when change were incongruent. There was a significant main effect of alignment, *F*(1,35) = 33.21, *MSE* = 0.22, *p* < .001, $\eta _{p}^{2}$ = .49, and a significant main effect of congruence, *F*(1,35) = 14.24, *MSE* = 0.29, *p* < .001, $\eta _{p}^{2}$ = .29. Importantly, the congruency $\times$ alignment interaction was significant, *F*(1,35) = 6.18, *MSE* = 0.12, *p* = .02, $\eta _{p}^{2}$ = .15.

Evidence for holistic processing can include both perceptual and decisional effects (Wegner & Ingvalson, 2002; Wenger & Ingvalson, 2003; Richler et al., 2008; Cornes et al., 2011; Mestry et al., 2012; Von Der Heide et al., 2018); hence, we additionally report the criterion estimates (see Fig. 4). Criterion estimates were higher in the aligned conditions indicated by a main effect of Alignment, *F*(1,35) = 4.57, *MSE* = 0.25, *p* = .04, $\eta _{p}^{2}$ = .12. The negative values indicate a shift in the criterion value for misaligned faces (see Fig. 4). The main effect of congruence and the two-way interaction were not significant.

## Discussion

The present findings allow confirmation that our schematic composite faces produced the appropriate interaction effect taken to be a hallmark of holistic processing. We next report the face stimuli and the results obtained for the SFT categorization experiment to determine whether processing second-order features in schematic composite faces is associated with coactivity or weaker forms of holistic processing. If there is a difference in processing between upright aligned faces and misaligned faces, then we would expect to see qualitatively different MIC, SIC, and contrast category patterns between our stimulus sets. Namely, if the strongest form of holism prevails, then we would expect to see the signature patterns of coactivity with upright aligned faces but not with misaligned faces.

## Experiment 2: Double factorial task method

We investigated the processing architectures underlying processing of our schematic composite face stimuli using the double-factorial design. In line with prior work, we adopt a small-N approach (Smith & Little, 2018; Little & Smith, 2018). The key difference between the small-N design and the large-N design (as used in Experiment 1) is that our aim in Experiment 2 is to test the formal predictions from each of the models. This differs from the large-N approach where identification of an effect and generalization to some population are the goal. In Experiment 2, each participant also generates a large amount of data for each item condition, allowing us to use computational modeling of RT distributions to further complement our non-parametric analyses and allowing each individual participant to act as a replication of the experiment. We refer to Smith and Little (2018) for further arguments as to the benefits of the small-N design.

### Participants

Twelve participants from The University of Melbourne were assigned to one of the Upright Aligned (N = 6) and Upright Misaligned (N = 6) conditions. If a participant did not complete all eight sessions, they were removed from further analyses. This resulted in the removal of participants in the Upright Aligned (N = 2) and Upright Misaligned (N = 2) conditions, all of whom completed three or fewer sessions.

A total of 8 participants (4 female, 4 male) aged 19-24 (*M* = 21, *SD* = 1.72) remained. Numbers of participants in each condition was based on the logic of small-N designs (Smith & Little, 2018); the number of trials for each item type (N = 400) was chosen so that the RT distributions were well-estimated.

Participants received $6 AUD per session with a $2 AUD bonus per session if accuracy was $$\ge $$ 90%. Each session was approximately 45 minutes in duration. Testing was approved under the Melbourne Human Research Ethics Committee 1034866.11. Data were collected in 2015.

### Stimuli and apparatus

#### Threshold study

We used a QUEST procedure (Watson & Pelli, 1983) to estimate the 75% discrimination threshold for the eye separation and lip height dimensions. To achieve this, a set of faces were created with eye separation varying from 90 to 116 pixels in 2 pixel increments and lip height varying from 0 to 25 pixels in 1 pixel increments. At the outset of the experiment, a comparison face was presented with values set to the midpoint of each of these ranges. On each trial, participants were randomly instructed to focus either on the eyes or the mouth. Participants had to respond whether the eye separation was narrower or wider than the reference image or whether the lip height was higher or lower than the reference image. This procedure was repeated until the QUEST procedure converged. Threshold data for the eye separation (*M* = -3.14, *SD* = 0.94) and lip height (*M* = -5.43, *SD* = 1.05) dimensions were collected from 5 participants. These thresholds were used to find suitable values for the contrast category (with narrow eye separation and high lip separation) and the target category (with wide eye separation and low lip separation).

Close, middle, and far eye separation corresponded to an eye distance of 96 pixels, 102 pixels, and 110 pixels respectively. Low, middle, and high lip separation corresponded to a lip height of 3 pixels, 12 pixels, and 22 pixels. The top and bottom halves were then combined to create a 3 $\times$ 3 matrix of composite faces see middle panel of Fig. 1. The misaligned faces were created by offsetting the top and bottom halves by 50 pixels.

The stimuli were presented on 1280 $\times$ 1024 resolution monitor with a refresh rate of 100 Hz set at a viewing distance of 70 cm. Each aligned face subtended a visual angle of 4.65 degrees $\times$ 3.85 degrees. Each misaligned face subtended a visual angle of 4.65 degrees $\times$ 3.96 degrees. The gap between the face halves subtended a visual angle of 0.11 degrees in all four conditions. A Gaussian filter was applied to blur the edges around each of the faces. RTs were collected using a calibrated response box (Li et al., 2010).

### Procedure

Each participant completed eight 45-minute sessions of testing, with the first session considered a practice session and not analyzed. Participants learned each of the categories via feedback. Participants were instructed to respond accurately but informed that RTs were being recorded. At the start of each trial, a fixation cross was presented for 1500 ms. A single face was then presented, and the participant was required to indicate whether the face belonged to the Target Category or the Contrast Category by pressing the appropriate button on the response box. Feedback was provided only if the response was incorrect (i.e., "...WRONG...") or after 5000 ms when the trial timed out (i.e. "...TOO SLOW..."). A blank interval of 2000 ms was inserted between trials.

Each session began with nine practice trials (each face presented once) followed by ten blocks of experimental trials. Each face was presented 50 times per session for a total of 400 times per participant across all eight sessions. In each session, there were ten blocks of 45 trials each, with the presentation order of the faces randomized within each block. Between blocks, participants were given feedback on their performance accuracy for the previous block and were allowed to take a short break.

### Data analysis

Each participant completed many trials per face stimulus condition; hence, we are interested in contrasting individual item performance in order to determine the specific processing architectures for each individual participant in each condition (see e.g., Little and Smith, 2018; Smith and Little, 2018). As such, we focus on individual participant ANOVAs looking at the factors of session (sessions 2-8) by discriminability on the top half (low or high) by discriminability on the bottom half (low or high) to assess the target category results. To assess the contrast category, we also conducted planned *t*-tests comparing the redundant stimulus to each of the other contrast category stimuli as well as comparing the interior to the exterior stimuli on both eye separation and lip height dimensions.

SIC results were analysed using methods adapted from Houpt and Townsend (2010); Houpt et al. (2014). We checked for stochastic dominance by comparing each relevant pair of survivor functions using a Kolmogorov-Smirnov test (i.e., to check that the HH survivor function was less than the HL survivor function, and so on). We additionally tested the maximum and minimum deflections in the SIC function (termed $D+$ and $D-$, respectively) using the calculations provided in Houpt and Townsend (2010).

Our manipulation of face alignment is between subjects and so we cannot directly compare the conditions; however, our non-parametric and parametric modeling analyses allow us to make consistent interpretations across conditions.

**Table 1:** Observed Mean Correct and Error RTs (ms) and Error Rates for Individual Stimuli for Each Observer

		Item								
Observer	Variable	HH	HL	LH	LL	Ex	Ix	Ey	Iy	R
UA1	RT correct	710	894	749	1041	894	747	1440	1042	670
	RT error	1751	1432	1442	1362	1308	2024	926	1107	-
	p(error)	.03	.25	.05	.42	.07	.01	.80	.22	-
UA2	RT correct	1163	1283	1392	1525	981	900	1564	1940	887
	RT error	1775	2301	1866	2162	1178	789	1812	1921	-
	p(error)	.05	.08	.12	.16	.02	.01	.30	.40	-
UA3	RT correct	615	688	741	807	727	648	756	662	575
	RT error	1059	1004	1082	846	752	696	768	816	478
	p(error)	.01	.10	.14	.41	.22	.05	.11	.03	.00
UA4	RT correct	1190	1571	1499	1940	1245	1171	1512	1672	1086
	RT error	1483	1558	1562	1540	1602	1527	1484	2015	1338
	p(error)	.03	.09	.06	.21	.07	.01	.13	.07	.01
UM1	RT correct	1031	1188	1219	1394	865	850	1276	1375	844
	RT error	-	-	2216	1958	1199	1296	1975	2310	-
	p(error)	-	-	.01	.03	.02	.01	.08	.05	-
UM2	RT correct	920	1006	1012	1105	673	658	1077	1091	644
	RT error	-	1255	1041	1475	1228	1200	1454	1413	-
	p(error)	-	.00	.01	.04	.02	.01	.05	.07	-
UM3	RT correct	909	1066	1176	1395	837	816	1155	1352	766
	RT error	933	1065	1444	1381	1020	1132	1353	1666	-
	p(error)	.01	.03	.07	.17	.08	.03	.05	.04	-
UM4	RT correct	1236	1492	1530	1964	1598	1484	1420	1321	1135
	RT error	-	1926	1979	2006	1586	2066	1973	2004	-
	p(error)	.00	.06	.05	.18	.19	.04	.07	.02	-

*Note.* UA = upright aligned; UM = upright misaligned; HH = high-high stimulus $x_2y_2$; HL = high-low stimulus $x_2y_1$; LH = low-high stimulus $x_1y_2$; LL = low-low stimulus $x_1y_1$; R = redundant stimulus $x_0y_0$; Ix and Iy denote the interior stimuli on the Lip Separation $x_1y_0$ and Eye Separation $x_0y_1$ dimensions respectively; Ex and Ey denote the exterior stimuli on the Lip Height $x_2y_0$ and Eye Separation $x_0y_2$ dimensions respectively. A ’-’ indicates that there were no errors for this stimulus

## Results

For each participant, Session 1 was considered practice and removed. Trials with RTs that were less than 200 ms or greater than three times the standard deviation plus the mean for each item condition were excluded. This led to the removal of less than 2% of the trials.

**Fig. 5:**
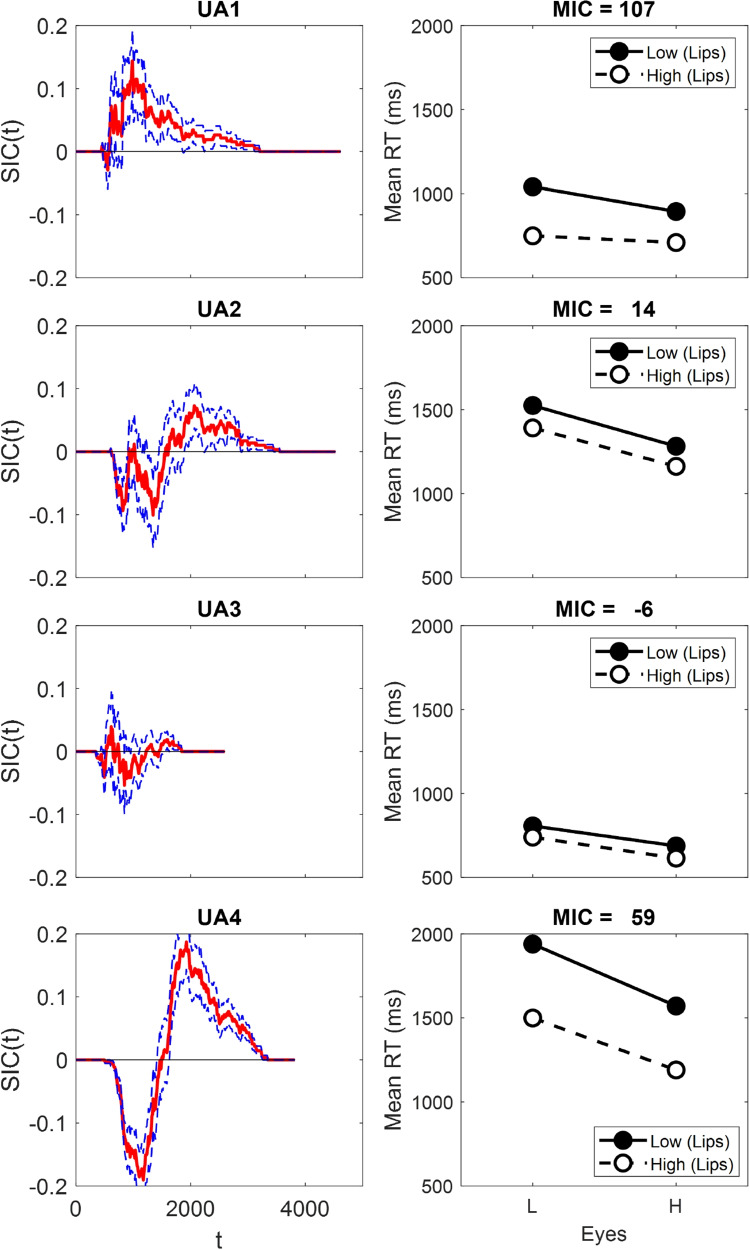
SIC and MIC results for the upright aligned conditions. Confidence bounds on the SIC curves were determined via bootstrapping. Error bars in the Mean RT figures are ± 1 standard error

Mean correct RTs, mean error RTs, and error rates for each participant are presented in Table 1. Participants are referred to by an abbreviation of their assigned condition (e.g., UA1 is the first participant in the upright aligned condition). As shown, error rates were high for some items (e.g., UA1 shows high error rates of 25% for item HL, 42% for item LL, and 80% for item Ey) although the Upright Misaligned condition displayed much lower error rates in comparison. We proceed with the analyses as the SIC is robust to errors in the AND condition (i.e., for the target items) so long as we can assume selective influence and process independnence, and so long as stochastic dominance is preserved (see Footnote 2; Little et al., 2022). The SIC and MIC results are shown in Fig. 5.

For the target category results, we ran a 7 (sessions 2-8) $\times$ 2 (top half: L or H) $\times$ 2 (bottom half: L or H) ANOVA on the target-category RTs for each participant (see Table 2. For the contrast category, we ran a series of planned *t*-tests comparing the redundant stimulus to each of the other contrast category stimuli on each dimension as well as comparing the interior to the exterior stimuli on both dimensions (see Table 3).

**Table 2:** Target Category Statistical Results for Individual Participants

Variable	*df*	*F*	*p*	$\eta _{p}^{2}$	*df*	*F*	*p*	$\eta _{p}^{2}$
	UA1				UM1			
Session	6	10.39	< .001	.05	6	55.73	< .001	.20
Session x Top	6	2.76	.01	.01	6	2.15	.046	.01
Session x Bottom	6	0.93	.47	.01	6	1.09	.36	.01
Top	1	145.25	< .001	.12	1	134.54	< .001	.09
Bottom	1	21.25	< .001	.02	1	190.8	< .001	.12
Top x Bottom	1	6.48	.01	.01	1	0.46	.50	.00
Top x Bottom x Session	6	0.49	.81	.00	6	1.19	.31	.01
Error	1116				1353			
	UA2				UM2			
Session	6	16.9	< .001	.08	6	54.31	< .001	.19
Session x Top	6	2.63	.02	.01	6	0.49	.82	.00
Session x Bottom	6	3.27	.003	.02	6	0.45	.85	.00
Top	1	19.77	< .001	.02	1	77.22	< .001	.05
Bottom	1	66.47	< .001	.05	1	84.34	< .001	.06
Top x Bottom	1	0.08	.78	.00	1	0.19	.67	.00
Top x Bottom x Session	6	0.78	.59	.00	6	1.03	.40	.01
Error	1245				1368			
	UA3				UM3			
Session	6	19.55	< .001	.09	6	20.24	< .001	.09
Session x Top	6	2.36	.03	.01	6	1.19	.31	.01
Session x Bottom	6	0.85	.53	.00	6	1.09	.37	.01
Top	1	38.95	< .001	.03	1	91.89	< .001	.07
Bottom	1	98.86	< .001	.08	1	226.59	< .001	.15
Top x Bottom	1	0.37	.54	<.001	1	2.76	.10	.00
Top x Bottom x Session	6	0.79	.58	.00	6	0.42	.87	.00
Error	1145				1280			
	UA4				UM4			
Session	6	36.45	< .001	.15	6	50.9	< .001	.19
Session x Top	6	7.06	< .001	.03	6	4.26	< .001	.02
Session x Bottom	6	5.34	< .001	.03	6	2.26	.04	.01
Top	1	285.64	< .001	.19	1	143.02	< .001	.10
Bottom	1	186.8	< .001	.13	1	168.44	< .001	.12
Top x Bottom	1	0.01	.91	.00	1	9.81	.002	.01
Top x Bottom x Session	6	1.39	.22	.01	6	0.35	.91	.00
Error	1245				1281			

All of the participants showed an effect of session (and, in some cases, an interaction between session and the top face half or session and the bottom face half, or both) indicating that response times decreased overall across session and that, in some cases, RTs to one or both of the dimensions decreased across sessions (e.g., the top dimension for UA1 in Table 2). The three-way interaction between top, bottom, and session tests whether the interaction effect changes across sessions, with the null hypothesis indicating no change across session. Crucially, the non-significant three-way interaction failed to reject this null hypothesis (see Table 2). As we can see from Table 4, for all observers, the HH RT distribution stochastically dominates the HL and LH distributions, which in turn dominated the LL RT distribution. This indicates that stochastic dominance is preserved and so we proceed with the SIC analyses for the target-category items.

**Table 3:** Contrast Category Statistics for Individual Participants

Variable	*M*	*t*	*df*	*p*	Cohen’s *d*	*M*	*t*	*df*	*p*	Cohen’s *d*
	UA1					UM1				
E_Top_ - I_Top_	6	5.25	669	< .001	0.40	6	0.90	690	.37	0.07
E_Bottom_ - I_Bottom_	6	5.34	340	< .001	0.64	6	-3.13	654	.002	0.24
E_Top_ - R	6	8.83	673	< .001	0.67	6	1.27	689	.20	0.10
I_Top_ - R	6	4.93	692	< .001	0.37	6	0.39	695	.70	0.03
E_Bottom_ - R	6	18.45	417	< .001	1.49	6	18.59	669	< .001	1.42
I_Bottom_ - R	6	13.06	619	< .001	1.00	6	20.63	679	< .001	1.57
	UA2					UM2				
E_Top_ - I_Top_	6	3.97	690	< .001	0.30	6	1.23	691	.22	0.09
E_Bottom_ - I_Bottom_	6	-5.94	460	< .001	0.55	6	-0.82	660	.42	0.06
E_Top_ - R	6	4.77	694	< .001	0.36	6	2.64	692	.008	0.20
I_Top_ - R	6	0.74	696	.46	0.06	6	1.37	693	.17	0.10
E_Bottom_ - R	6	17.64	597	< .001	1.36	6	31.63	680	< .001	2.41
I_Bottom_ - R	6	26.61	563	< .001	2.07	6	31.31	674	< .001	2.39
	UA3					UM3				
E_Top_ - I_Top_	6	5.42	603	< .001	0.44	6	0.88	659	.38	0.07
E_Bottom_ - I_Bottom_	6	6.62	646	< .001	0.52	6	-6.09	667	< .001	0.47
E_Top_ - R	6	13.26	622	< .001	1.02	6	3.35	670	.001	0.26
I_Top_ - R	6	7.66	677	< .001	0.58	6	2.47	685	.01	0.19
E_Bottom_ - R	6	15.00	658	< .001	1.14	6	16.59	678	< .001	1.27
I_Bottom_ - R	6	9.80	684	< .001	0.74	6	21.06	685	< .001	1.60
	UA4					UM4				
E_Top_ - I_Top_	6	2.44	673	.02	0.19	6	2.64	621	.009	0.21
E_Bottom_ - I_Bottom_	6	-4.37	631	< .001	0.35	6	2.28	671	.02	0.18
E_Top_ - R	6	5.47	673	< .001	0.42	6	12.93	630	< .001	1.01
I_Top_ - R	6	3.17	694	.002	0.24	6	10.18	683	< .001	0.78
E_Bottom_ - R	6	14.60	651	< .001	1.14	6	7.49	673	< .001	0.57
I_Bottom_ - R	6	17.78	674	< .001	1.36	6	5.55	690	< .001	0.42

*Note.* UA = upright aligned; UM = upright misaligned; E = exterior; I = interior; R = redundant. The subscript Top or Bottom refers to which dimension is varying in the comparison

**Table 4:** Stochastic Dominance Results for Each Observer

Test	KS	*p*	KS	*p*	KS	*p*	KS	*p*
	UA1		UA2		UA3		UA4	
S_HH_ > S_HL_	0.30	< .001	0.16	< .001	0.16	< .001	0.43	< .001
S_HH_ > S_LH_	0.14	< .05	0.23	< .001	0.32	< .001	0.32	< .001
S_HL_ > S_LL_	0.20	< .001	0.18	< .001	0.33	< .001	0.32	< .001
S_LH_ > S_LL_	0.35	< .001	0.15	< .05	0.18	< .001	0.40	< .001
S_HH_ < S_HL_	0.00	> .99	0.01	.98	0.00	> .99	0.00	> .99
S_HH_ < S_LH_	0.02	.82	0.00	> .99	0.01	.99	0.00	> .99
S_HL_ < S_LL_	0.03	.79	0.00	.99	0.01	.96	0.00	> .99
S_LH_ < S_LL_	0.00	> .99	0.00	> .99	0.01	.99	0.00	> .99
	UM1		UM2		UM3		UM4	
S_HH_ > S_HL_	0.27	< .001	0.20	< .001	0.26	< .001	0.22	< .001
S_HH_ > S_LH_	0.31	< .001	0.23	< .001	0.34	< .001	0.23	< .001
S_HL_ > S_LL_	0.31	< .001	0.23	< .001	0.28	< .001	0.29	< .001
S_LH_ > S_LL_	0.24	< .001	0.20	< .001	0.22	< .001	0.29	< .001
S_HH_ < S_HL_	0.00	> .99	0.00	> .99	0.00	> .99	0.00	> .99
S_HH_ < S_LH_	0.00	> .99	0.01	.99	0.00	> .99	0.00	> .99
S_HL_ < S_LL_	0.01	.99	0.01	.97	0.00	> .99	0.00	> .99
S_LH_ < S_LL_	0.00	> .99	0.00	> .99	0.00	> .99	0.00	> .99

*Note.* UA = upright aligned; UM = upright misaligned. If stochastic dominance is met, the first four tests should be statistically significant, while the last four tests should not. KS stands for the Kolmogorov-Smirnov test statistics which is the maximum difference between the CDFs of the two item conditions being tested

We focus on the two primary diagnostic measures - the two-way interaction (MIC) between eye separation and lip height for the target category stimuli and the interior versus exterior comparison for the contrast category stimuli - for each face condition. To summarize the diagnostic measures, a non-significant target category MIC near zero along with slower interior versus exterior contrast category items is consistent with serial processing. Parallel processing is consistent with a significant negative MIC coupled with a negative SIC and a non-significant difference in the mean RTs for interior versus exterior contrast category items. Lastly, a significant positive MIC along with a mostly positive SIC and faster mean RTs for interior compared to exterior items indicates coactive processing.

Across all conditions, only UM4 showed a significant top $\times$ bottom interaction. Hence, although there was some evidence of an interaction between the top and bottom face half, it was not found in the aligned condition as expected. Inspection of Fig. 5 shows that, in contrast to the statistical results (see Table 5), UA1 does have what appears to be an overadditive SIC.

The contrast category results suggest processing differences between the participants (see Table 3 and Fig. 7, left column). For participant UA1, the analysis indicates coactive processing due to the faster mean RTs for interior compared to exterior items. Participant UA3 also displays RTs consistent with coactivity as the interior is faster than the exterior items on both dimensions. However, RTs on both dimensions are very similar to each other. Results for participants UA2 and UA4 show the exterior stimulus is faster than the interior stimulus for the lip height dimension. However, the interior stimulus is faster than the exterior stimulus on the Eyes dimension, making the contrast category results for participants UA2 and UA4 inconsistent with any of the models (Fig. 5).

While the SIC and contrast category results sometimes suggest coactivity for participants in the aligned condition (e.g., UA1 and UA3), the interaction contrast was non-significant. Target category results for participants UA2 and UA4 look serial, and this inference is supported by non-significant MICs. However, for both these participants, while the lip height dimension shows longer interior than exterior RTs, the Eyes dimension shows longer exterior compared to interior RTs. Overall, the aligned contrast category results are only partially consistent with coactivity.

**Fig. 6:**
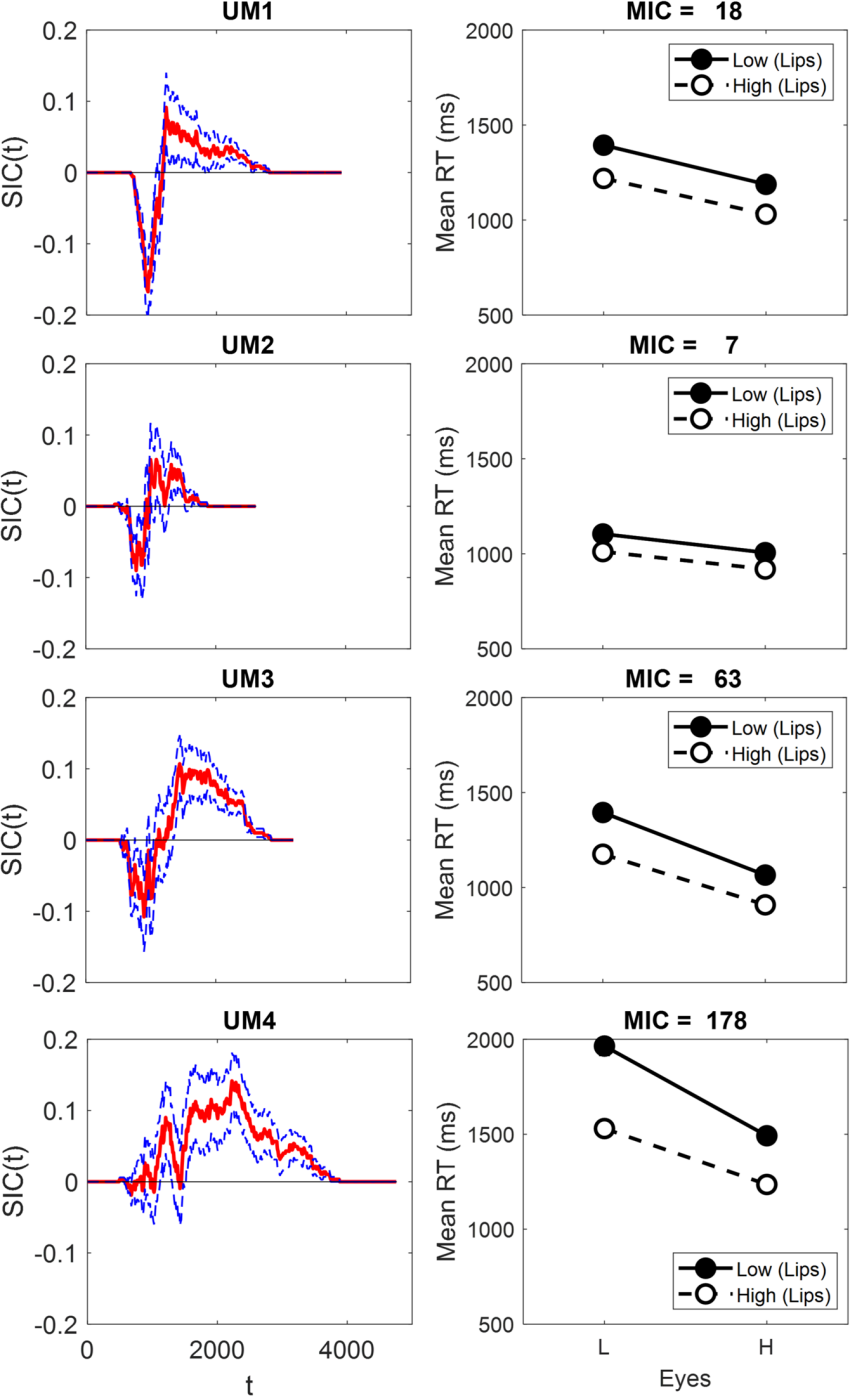
SIC and MIC results for the Upright Misaligned face conditions. Confidence bounds on the SIC curves were determined via bootstrapping. Error bars in the Mean RT figures are ± 1 standard error

When misaligned, performance on upright faces looks mostly consistent with serial processing (for UM1, UM2, and UM3). While noisy, the SIC for participant UM4 displays some characteristics indicative of coactive processing (Fig. 6). The significant eye separation $\times$ lip height interaction confirms that the MIC was positive (see Table 2, right hand columns), thus supporting an inference of coactive processing for participant UM4.

**Fig. 7:**
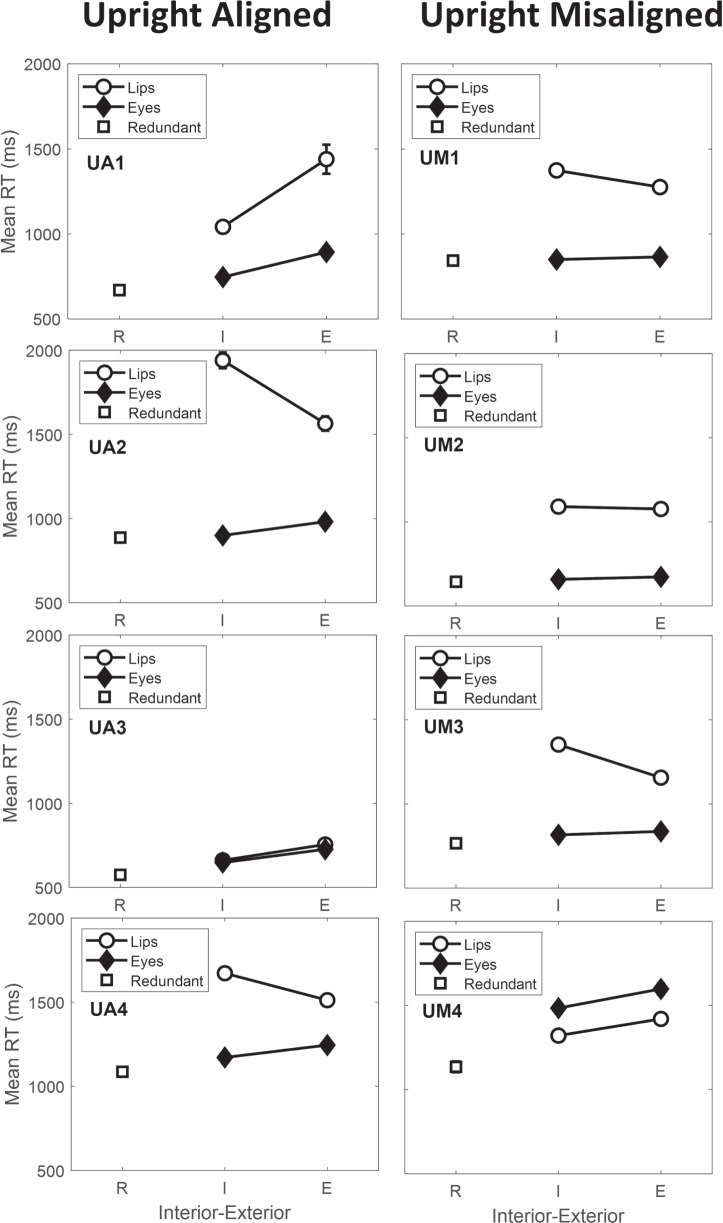
Observed contrast category mean RTs for the aligned (left column) and misaligned (right column) participants. Error bars represent ± 1 SE. Some of the standard error bars for the mean RTs are too small to be seen. The label “Nose-Mouth” refers to items which satisfy the category B rule on the lip height dimension (i.e., the vertical boundary) but vary on the Eyes dimension values. Correspondingly, the label “Eye” refers to items which satisfy the category B rule on the Eyes dimension (i.e., the horizontal boundary) but vary on the lip height dimension values

For the contrast category results, there was no significant difference between the interior and exterior items for UM2 (see Table 3 and Fig. 7, right column), suggesting parallel self-terminating processing. Participants UM1 and UM3 had longer interior RTs compared to exterior RTs on the lip height dimension but no significant difference between interior and exterior items on the Eyes dimension. These results are indicative of fixed-order serial self-terminating processing for both participants (see Fig. 3). Lastly, for participant UM4, the analysis indicates coactive processing due to the faster mean RTs for interior compared to exterior items on both dimensions.

**Table 5:** Statistical Significance of the Positive and Negative Parts of the SIC for Each Observer

Observer	D+	*p*	D-	*p*
UA1	0.14	.06	0.03	.89
UA2	0.07	.43	0.10	.20
UA3	0.04	.80	0.05	.67
UA4	0.19	.003	0.19	.003
UM1	0.09	.24	0.17	.008
UM2	0.07	.47	0.09	.24
UM3	0.11	.15	0.11	.15
UM4	0.14	.04	0.02	.94

*Note.* UA = upright aligned; UM = upright misaligned. D+ refers to the largest positive value of the SIC and D- refers to the largest negative value of the SIC

### Discussion

One main conclusion that can be drawn from the analyses above is that there is no strong evidence of coactive processing in either condition. While there were a few observers who displayed faster interior mean RTs compared to exterior mean RTs, this was seldom accompanied by an overadditive MIC or SIC in the target category.

We also did not find any evidence for exhaustive processing as the redundant stimulus in the contrast category was always processed as fast as or faster than the interior or exterior stimuli. Most of the participants had results that resemble the predictions of parallel or serial processing architectures. In addition, some participants also exhibited predictions which seem to fall between the predictions of these two architectures.

To provide further clarity to our results, we fit and compared several parametric computational models that allow us to test mixtures of serial and parallel processing as well as a more flexible model of coactivity. These models have the added the benefit of allowing us to simultaneously fit both correct and error RTs. Prior to applying the computational models to our data, we first report a multidimensional scaling study which provides us with coordinates that can be used to approximate the psychological representation of the stimuli used by the models.

## Experiment 3: Multidimensional scaling study

To understand the psychological representation of the nine schematic composite faces used in the categorization task, we obtained similarity ratings for each pair of faces via an additional study on the Amazon Mechanical Turk platform. We then compared several multidimensional scaling (MDS) analyses to determine the most parsimonious representation of the faces at each orientation and alignment.

### Method

#### Participants and apparatus

Participants were randomly allocated to the aligned (N = 23) or misaligned (N = 21) condition. All participants were recruited from the Amazon Mechanical Turk platform. Each participant took approximately 30 minutes and received $2 for completion of the task. Testing humans was approved by the University of Melbourne Human Research Committee 1340152.1.

Each experimental condition was programmed in Javascript and HTML and completed by the participant via a browser on their own computer.

**Table 6:** Summary of Sum of Squared Deviations and Bayesian Information Criterion for Each Model

		Full Model		
Condition	N	City block	Euclidean	Minkowski
UA	12	321.42 (42.18)	287.36 (-6.21)	300.42 (19.07)
UM	9	281.36 (98.81)	273.39 (89.49)	283.85 (107.44)
		Constrained Model		
Condition	N	City block	Euclidean	Minkowski
UA	12	364.7 (23.94)	**283.44 (-84.96)**	285.05 (-76.44)
UM	9	306.48 (57.14)	**277.31 (24.73)**	273.22 (25.70)

*Note.* Cond = Condition, UA = Upright Aligned, UM = Upright Misaligned. BIC values are presented within parentheses. The best fitting model is bolded

#### Stimuli and procedure

Face stimuli used in this experiment were identical to the categorization experiment stimuli. In each of the four face conditions, there were 36 unique pairings of the 9 face stimuli. Each pair of stimuli was presented three times in a random order across the experiment. The presentation location of each face on the screen (i.e., left vs right) was also randomized.

Participants were instructed to use the entire response scale at the outset of the experiment and to respond as honestly as possible. At the beginning of the experiment, participants were sequentially presented with all nine faces in random order (e.g., either in an aligned or misaligned fashion, depending on the condition). Each face was presented for 500 ms. At the start of each experimental trial, a fixation cross was shown for 500 ms. Participants were then presented with a pair of faces, which they rated from 1 (Least Similar) to 8 (Most Similar). The experiment was self-paced with each trial initiated by a key press.

#### Data analysis

Participants were first screened to determine whether they had used the full response scale. Histograms of the frequency of responding with each scale option were plotted; participants were removed upfront if they did not use all of the response options or had a much higher frequency of using one or two response options compared to the remainder. Seven participants were removed from the aligned (updated N = 15) and misaligned conditions (updated N = 14).

To find the best fitting coordinates, we compared a number of models by estimating the X and Y coordinates of the stimuli as free parameters (i.e., the psychological locations of each value of the top and bottom face halves). This method is similar to the confirmatory MDS method described in Borg and Groenen (2005). In the *free coordinate* model, the coordinates were only constrained to be monotonically increasing separately at each level of the X and Y dimensions. This model had 16 coordinate parameters (the X and Y coordinates of the first stimulus were fixed at 0, 0). We also fit a version of the model where the X and Y coordinates were constrained to be equivalent across each level of X and Y. This constraint forces the stimuli to lie on a grid while still allowing differences between the levels of X and Y. This model had 4 coordinate parameters; here the first values of X and Y were set to 0.

**Fig. 8:**
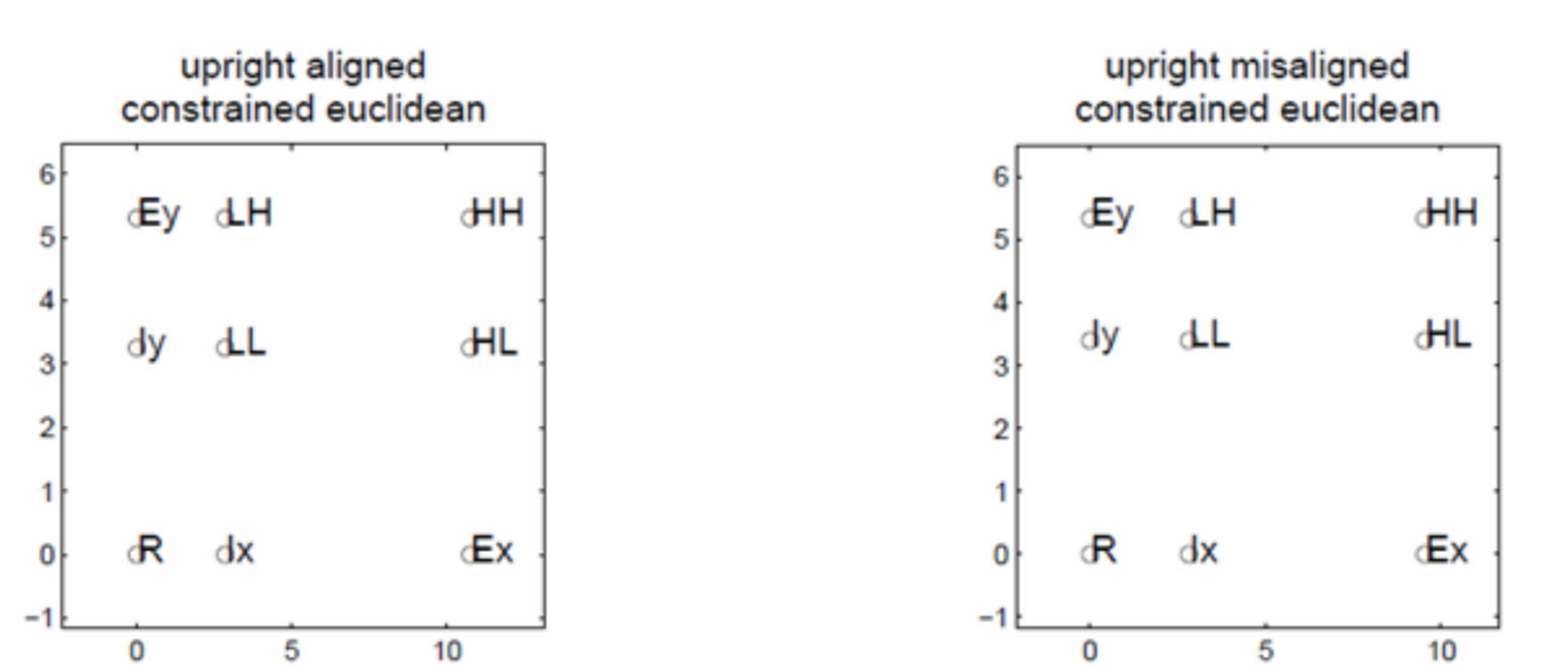
Best-fitting MDS solutions

We used an Individual Differences Scaling (INDSCAL) method (Carroll & Chang, 1970) and estimated a weight for each participant which was then used as a scaling factor in the distance calculation. This allows the distances for each participant to be scaled from a common set of coordinates. This added one additional parameter for each participant to each model.

We compared the distances between the estimated coordinates to the similarity rating data by calculating the sum of squared deviations (SSD) using either a city-block metric ($r=1$), a Euclidean metric ($r=2$), or by allowing r to vary as a free parameter. The SSD for each model was converted to a BIC by assuming a Gaussian likelihood function using the following equation:

3 $$\begin{aligned} BIC = -2\log (L) + k\log (n) \end{aligned}$$

where *L* is the likelihood, *k* is the number of parameters, and *n* is the number of data points used to estimate the likelihood.

### Results and discussion

Table 6 shows the sum of squared deviations and Bayesian Information Criterion for each model. As shown in Fig. 8, the MDS solutions align closely with our schematic distance settings. The Constrained Euclidean model has the lowest BIC for both of the upright conditions. This solution with a Euclidean distance metric is consistent with the idea that the top and bottom halves of the composite faces are integral dimensions (Garner, 1974; Griffiths et al., 2017; Shepard, 1987; Shepard & Chang, 1963).

We highlight that these results differ markedly from what was observed with the morph composite faces used in our earlier study (Cheng et al., 2018). In that study, the MDS results supported use of a city-block metric with values of *r* estimated to be close to 1 for the upright aligned and misaligned conditions (Attneave, 1950; Nosofsky, 1992; Shepard, 1987; Shepard & Chang, 1963; Torgerson, 1958; Shepard, 1991). This suggests that the top and bottom halves of our schematic face morphs are closer to integral dimensions than separable dimensions providing a further rationale for contrasting schematic composites with morph composites.

Nevertheless, the best models across all of our face sets force the faces to be constrained to a grid. Such a constraint suggests a dimensional representation where the lip height and eyes dimensions are represented independently. MDS is not able to provide further insight (cf. Howard et al., 2017), but we use the coordinates for each face condition in our computational modeling which we turn to next.^3^

## Computational modeling

To provide further clarification of our non-parametric results, we fit a set of computational models that allow us to test parametric implementations of the main processing architectures described above, as well as mixtures of serial and parallel processing and a more flexible model of coactive processing. Unlike with the SFT analyses, we also fit the error RTs and fit all of the data simultaneously. Hence, our computational modeling has some advantages over the nonparametric SFT analyses, albeit requiring specific parametric assumptions to be made about the RT distributions.

To begin, we fit a set of logical rule-based models (Fific et al., 2010b) in which the processing of each dimension is modeled as an evidence accumulation process (Brown & Heathcote, 2008; Luce, 1986; Nosofsky & Palmeri, 1997; Ratcliff, 1978). The predicted RTs for processing each stimulus dimension are then combined using logical OR or AND operations depending on the specific mental architecture. For example, for the AND rule in a serial self-terminating model, the two independent processing times are summed. By contrast, for the AND rule in a parallel exhaustive model, the RT is the maximum of the two independent processes. In contrast, the corresponding self-terminating processes would use an OR rule for certain stimuli such as the contrast category stimuli. Due to the logical rules design of our study, we focus our analyses on self-terminating models as the analyses of the contrast category rule out exhaustive processing.

Based on General Recognition Theory (GRT; Ashby and Townsend, 1986) and decision boundary theory (Ashby & Gott, 1988), the logical rule models assume that the perception of any stimulus dimension is noisy and that repeated presentations may not result in the same percept. Hence, the distribution of percepts for each stimulus dimension is represented as a normal distribution with a mean given by the MDS coordinates and a freely estimated variance parameter for each dimension. Note that the mean locations must be fixed to allow drift rates to be identifiable, but the locations can be translated or scaled (with appropriate transforms to the decision bound and item variances) without changing the predictions of the models.

For the serial and parallel models, samples are taken from the marginal distribution on each dimension which is then used to drive independent sequential sampling decision processes. If a participant samples from the lip height dimension and that identifies it as belonging to the target category, then evidence is accumulated towards the target category. For the coactive model, each stimulus location is identified by a bivariate normal distribution of perceptual effects. These perceptual effects along the two dimensions (e.g., lip height and eyes dimensions) are statistically independent for each stimulus.

It is assumed for all models that all stimuli have the same perceptual variability along the two dimensions but that this variability differs between dimensions. As such, we have separate free parameters, $\sigma _{lips}$ and $\sigma _{eyes}$ for the standard deviation of each dimension. The means, $\mu$, of the perceptual distributions were fixed to the coordinate values obtained from the MDS solutions for each specific face stimulus (see MDS section above).

The models assume that the location of the decision bounds on the dimensions $D_{lips}$ and $D_{eyes}$, respectively, are established by each participant in order to differentiate the target and contrast category. The evidence for each category is obtained by integrating the bivariate normal distribution within each of the category regions. This evidence is then used as the rate of evidence accumulation, or drift rate *v*, in the sequential sampling model.

While previous applications of the logical rule models have used the random-walk model which is a discrete time sequential sampling model, we followed Cheng et al. (2018) and used the Linear Ballistic Accumulator model (LBA; Brown and Heathcote, 2008) to predict the RTs from each model.

### LBA and the logical rule models

The LBA generates RTs by assuming that two accumulators race against each other with the faster of the two accumulators generating the decision time for that trial. A single accumulator’s finishing time is determined by the relationship between the distance between the starting point of accumulation and the threshold for making a decision where the rate of evidence accumulation is given by a standard velocity function: time = distance/rate. To achieve variability in the RT distribution, the LBA assumes that the start point for each accumulator varies from trial to trial according to a uniform distribution (parameter *A* provides the range of this distribution) and that the drift rate varies from trial to trial according to a normal distribution (parameter *s* provides the drift rate standard deviation). The LBA also has two threshold parameters, one for the target category accumulator, and one for the contrast category accumulator. Each model also assumes a non-decision time parameter, $t_0$, that captures time not associated with decision-making processes (e.g., processes such as encoding or motor execution). The final predicted RT is the sum of the decision time predicted by the LBA and the non-decision time.

For the serial and parallel architectures, there are separate accumulators for the lip height and eye separation dimensions. By contrast, for the coactive model, there is a single accumulator for the entire face. Each pair of accumulators contains one accumulator corresponding to the target category and one accumulator corresponding to the contrast category.

To determine the drift rates (e.g., for the lip height dimension), we integrate the marginal distribution with respect to the decision boundary to determine the area under the curve that falls into each category region along that dimension; hence, the drift rates for the correct and error accumulators sum to one.

**Table 7:** DICs (and DIC weights) for each model and each participant

	Model							
Condition	Participant	Serial ST	Mixed SC	Parallel ST	Mixed PC	Mixed SP	Coactive	Free Drift
UA	UA1	2753 (0)	2774 (0)	2517 (0)	2522 (0)	**2348 (1)**	2565 (0)	2945 (0)
	UA2	5328 (0)	5332 (0)	5227 (0)	5242 (0)	**4955 (1)**	5409 (0)	5324 (0)
	UA3	-889 (0)	-912 (0)	-1040 (0)	-1026 (0)	**-1058 (1)**	-761 (0)	-674 (0)
	UA4	4964 (.14)	**4960 (.86)**	5024 (0)	5009 (0)	4974 (0)	5289 (0)	5251 (0)
UM	UM1	785 (0)	**764 (1)**	869 (0)	804 (0)	795 (0)	959 (0)	1373 (0)
	UM2	-1246 (.02)	-1222 (0)	-766 (0)	-1018 (0)	**-1254 (.98)**	-713 (0)	-538 (0)
	UM3	2541 (.02)	**2534 (.85)**	2884 (0)	2864 (0)	2538 (0)	2924 (0)	3202 (0)
	UM4	6219 (0)	6219 (0)	6205 (0)	6276 (0)	**6100 (1)**	6363 (0)	6365 (0)

*Note.* UA = Upright Aligned, UM = Upright Misaligned, ST = Self-terminating, SP = Serial-Parallel, SC = Serial Contaminant, PC = Parallel Contaminant. The lowest DIC model is bolded

Across the models, there are nine free parameters in total: the perceptual standard deviation parameters ($\sigma _{lips}$ and $\sigma _{eyes}$); location of the decision-bounds on the lip height and eyes dimensions ($D_{lips}$ and $D_{eyes}$); the range of the start-points for evidence accumulation (*A*); the correct and error thresholds ($b_{Target}$ and $b_{Contrast}$); drift rate variability (*s*); and non-decision time ($t_0$). The serial self-terminating model requires an additional parameter, $p_x$ representing the probability that, on a given trial, the lip height dimension is processed before the eyes dimension.

We also fit an additional independent channel model that assumed that for any stimulus, the lip height and eyes dimensions may be processed in parallel on some of the trials and serially on other trials. This mixture of processing might represent fluctuations in controlled attention across the course an experiment (e.g., Little et al., 2019; Schneider and Shiffrin, 1977). The mixed serial-parallel model also includes the same $p_x$ parameter as in the serial self-terminating model. In addition, the mixed serial-parallel model includes another extra parameter, $p_{Serial}$, which represents the proportion of serial trials. To allow for differences between the parallel and serial components, we multiplied the perceptual variability parameters in the parallel component by a freely estimated constant, $\sigma _{Parallel} = m\sigma _{Serial}$. We also estimated a separate starting point parameter, $A_{Parallel}$, for the parallel model component.

We additionally fit two further models, a mixed serial contaminant model and a mixed parallel contaminant model. Contaminant RTs are RTs assumed to be generated by a cognitive process other than the one that is being studied (Ratcliff & Tuerlinckx, 2002) and can represent processes such as guessing. Those contaminant RTs that overlap the distribution of RTs from the process under investigation can be corrected for during the fitting process, thereby improving the parameter estimates. An assumption in these two contaminant models is that contaminants come from a uniform distribution with a range determined by the minimum and maximum response times in each experimental condition, with the probability of a contaminant, $p_0$, being independent of the stimulus (Ratcliff & Tuerlinckx, 2002). As such, the distribution of response times from the LBA is weighted with probability 1$- p_0$ and the uniform distribution is weighted with probability $p_0$.

To model coactivity, we assume that the stimulus is represented by a joint bivariate perceptual distribution. In this model, a single drift rate is estimated by integrating the bivariate distribution with respect to both decision boundaries. To provide a more powerful test of coactivity that is unconstrained by our assumptions of the locations or variances of each stimulus, we fit a *free-stimulus-drift* model which estimates the drift rate associated with each stimulus as a free parameter. If we find that our constrained independent channel models fit better than this highly flexible model, then this would provide a strong result arguing against coactivity.

**Fig. 9:**
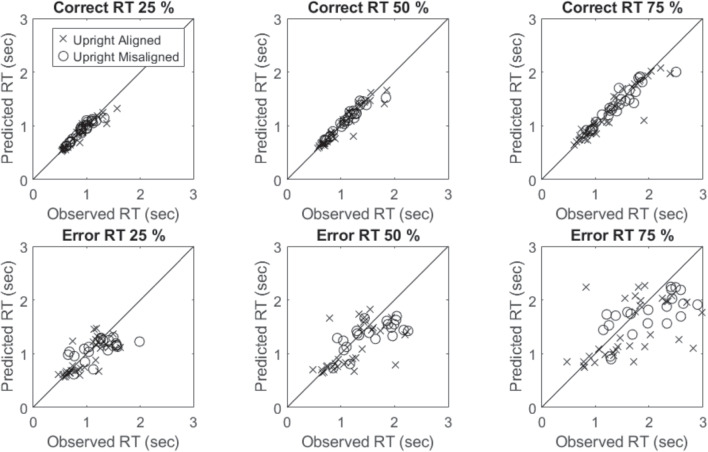
Posterior predictions of the Mixed Serial-Parallel model for the 25, 50, 75 tertiles for the correct and mean RTs for each item and each participant

**Table 8:** Summary of Nonparametric and Parametric Results for Upright Face Participants

Participant	SIC	MIC	Interior vs Exterior	Nonparametric Result	Best Parametric Model
UA1	Positive	Sig.	I < E	Coactive	Mixed S-P
UA2	S-shaped, neg. then pos.	Non-sig.	I < E Top, I > E Bot	Serial ST	Mixed S-P
UA3	Mostly negative	Non-sig.	I < E	Parallel ST	Mixed S-P
UA4	S-shaped, neg. then pos.	Non-sig.	I < E Top, I > E Bot	Coactive/Serial ST	Mixed S-C
UM1	S-shaped, neg. then pos.	Non-sig.	I > E Bot	Serial ST	Mixed S-C
UM2	S-shaped, neg. then pos.	Non-sig.	I = E	Parallel/Serial ST	Mixed S-P
UM3	S-shaped, mostly pos.	Non-sig.	I > E Bot	Serial ST	Mixed S-C
UM4	Positive	Sig.	I < E	Coactive	Mixed S-P

*Note:* sig = significant, non-sig = non-significant, pos = positive, neg = negative, ST = Self-terminating, I = interior, E = exterior, S-C = Serial-Contaminant, S-P = Serial-Parallel, Bot = Bottom. The Nonparametric Result is found by interpreting the SIC, MIC, and Interior vs Exterior results in that order. Where multiple interpretations are possible, all are listed

### Fitting procedure

To compute likelihoods for each item, we simulated 50,000 data points from the model and used (Holmes, 2015) probability density approximation method (PDA; see also Turner and Sederberg, 2014) to determine the likelihood of each decision response and each observed RT. The likelihood of the parallel and coactive logical rule models (LR) is given by the product over RTs and response on each trial:

$$\begin{aligned} &  L(D_{lips},D_{eyes},\sigma _{lips}^2,\sigma _{eyes}^2,A,b_A,b_b,s,t_0|resp,rt)\\= &  \prod _{i = 1}^{N}LR(resp_i, rt_i|D_{lips},D_{eyes},\sigma _{lips}^2,\sigma _{eyes}^2,A,b_A,b_b,s,t_0) \end{aligned}$$

The remaining models have other parameters such as the additional parameter, $p_x$, in the serial self-terminating model. The mixed serial-parallel model has four additional parameters ($p_x$, $p_{serial}$, *m*, $A_{parallel}$). The mixed serial and parallel contaminant models have an additional free parameter, $p_0$. The free drift model replaces the GRT parameters with freely-estimated drift rates for each item ($\nu _{HH}$,$\nu _{HL}$,$\nu _{LH}$,$\nu _{LL}$,$\nu _{Ex}$,$\nu _{Ix}$,$\nu _{Ey}$,$\nu _{Iy}$,$\nu _R$).

We used a Differential Evolution Markov chain Monte Carlo (DE-MCMC; Turner et al., 2013) to generate proposals from the posterior distributions of each parameter.

To compare the models, we used the Deviance Information Criterion (DIC; Spiegelhalter et al., 2002). The deviance of a posterior sample of parameters, $\theta$, is computed as $D(\theta ) = -2\textrm{ln}L(y|\theta )$. The DIC is computed as:

4 $$\begin{aligned} DIC = \overline{D}(\theta )+2p_D \end{aligned}$$

where $\overline{D}(\theta )$ is the mean of the distribution of posterior deviances and $p_D = \overline{D}(\theta )-D(\overline{\theta })$. $\overline{\theta }$ is the average posterior parameter value.

Consequently, DIC penalizes the average negative log likelihood by a term which accounts for the functional form complexity of the model. Thus, the model that yields the smallest DIC is preferred. The DIC values for each participant and each model are presented in Table 7.

### Summary of model fits

For most participants, the most preferred model was the mixed serial-parallel model. This model was preferred for three participants in the aligned condition and for two participants in the misaligned condition. For the remaining participants, the preferred model was the serial or mixed serial-contaminant model. Overall, we did not find any evidence for coactivity in either of the conditions. We also did not observe any strong differences between the conditions.

We plot the 25, 50, and 75 percentiles from the Mixed Serial-Parallel model for the correct and mean RTs for each item and each participant in Fig. 9. The model fit the data well and the parameter estimates were sensible. Detailed posterior predictive distributions for each participant and each item, along with posterior parameter distributions, are available in the materials.

### Discussion

As for the morphed faces (Cheng et al., 2018), the schematic faces did not show strong evidence of holistic processing. A summary of both the non-parametric and parametric results is provided in Table 8. While there are some discrepancies arising between the non-parametric analyses and the parametric computational modelling, we note that the modelling results take into account both the correct and error RTs for all items simultaneously. As such, we focus our discussion on the outcome of our computational model fitting.

Our modeling results show that processing was mostly consistent with a mixture of serial and parallel processing. This form of mixture model has also been found to provide good fits for different stimulus types such as overlapped separable dimensions (Little et al., 2011, Experiment 2), whole object features (e.g., size, colour, and saturation varied within a single object; Moneer et al., 2016), and particularly also with morphed composite faces (Cheng et al., 2018). As such, our results suggest that the composite faces were largely treated similarly to other objects with separable dimensions.

Importantly, while the faces we used appear to be processed holistically in the standard composite task, there is no consistent evidence for coactivity when participants categorize upright aligned faces. We also did not find that aligned faces are treated differently from misaligned faces. Based on our non-parametric and parametric results, we can also see that the main differences between the face conditions were quantitative rather than qualitative (see Richler et al. 2011b).

## General discussion

We examined processing architectures underlying face processing using a set of composite schematic faces that varied on two second-order features: the distance between the nose and mouth (lip height) and the distance between the eyes (eye separation). In particular, we were interested in investigating whether composite faces were processed holistically by pooling information across both dimensions into a single processing channel or whether some other form of processing explained the difference between aligned and misaligned schematic composites.

Although we have conducted similar analyses using composite faces comprised of face morph top and bottom halves, there is ample empirical evidence and theoretical argument highlighting that schematic second-order features are processed differently than morph features. Indeed, we show in our MDS study (Experiment 3) that our schematic composites are consistent with an assumption of integrality as it is typically inferred using similarity ratings (see Griffiths et al., 2017). Taken together with the composite face effect found in Experiment 1, we expected to find some evidence of strong holistic processing, namely coactivity, in our double factorial experiment (Experiment 2).

By comparing across a number of different processing models using a set of strong non-parametric and parametric inference techniques, we found little evidence for coactive processing of schematic composite faces. We also found little evidence of a difference between conditions; instead, processing of faces across the aligned and misaligned face conditions consisted of mostly a mixture of serial and parallel processing. These results can be added to several recent studies which have failed to find evidence of strong holism with composite faces (Fitousi, 2015, 2020, 2021; Cheng et al., 2018; Fifić et al., 2025).

From the perspective of the mixed serial-parallel model, the implication is that we should generally find more of an influence of the serial component when faces are misaligned. Our participant sample and small-N design preclude us from making a definitive conclusion, but examining the median values of the average $p_{serial}$ parameter based on the fits of the mixed serial-parallel model to each participant (UA = .33, UM = .99) , we do find that misalignment increased the probability of serial processing.

### Holistic processing in different tasks

One further potential explanation for the difference between our composite face task results and our categorization results is related to work carried out by Lynch et al. (2021) which combined the complete composite face task with a signal-to-respond task. The signal-to-respond task allows us to manipulate the amount of time to make a face recognition decision. Like in the composite face task, the participant was instructed to attend to one face half. They were then shown a study face followed by a test face that had either a congruent or incongruent change and asked to make a same-different judgment. A response signal appeared at one of six time lags between 50 to 1800 ms, and resulting changes in accuracy were mapped over the time course of processing.

Their study found that the interaction effect (i.e., the marker of holistic processing) emerged late in the time course of processing - after 400 ms for easy-to-discriminate faces, and after 1000 ms for difficult-to-discriminate faces. This suggests that as discriminations get harder, the interaction between alignment and congruency takes longer to arise; hence participants might respond before holistic processing comes about. This may provide a methodological explanation for our findings from the SFT task; discriminations are difficult to make and may result in holistic processing only emerging later in the time course of processing (e.g., after the response).

### Towards an information processing approach to understanding face processing

We note that the composite face task and the SFT double factorial task require different types of decisions. The composite face task might be thought of as a change detection task or recognition memory task, whereas the double factorial experiment requires a categorization decision. One line of argument against our investigation is that different tasks may produce different results simply by virtue of requiring different decisions. To preempt this line of argument, we note that categorization, recognition, and change detection are related tasks that can be modeled using the same underlying theoretical mechanisms (cf. Nosofsky et al., 2011; Nosofsky et al., 2012; Blunden et al., 2022; Fifić et al., 2010a; Cropper et al., 2013). A second objection might be that our analyses rely on different dependent variables. The composite task focuses on accuracy and d-prime, while our categorization task uses response time (RT) as the main dependent variable. There is now a large body of literature which identifies the relations between accuracy and RT (e.g., Ratcliff and McKoon, 2008); without reiterating this work, a fundamental takeaway is that accuracy and RT should be treated together. Our computational modeling focuses on analysis of both correct and error RT distributions.

Finally, the composite face task is a selective attention task (Richler & Gauthier, 2013) whereas the categorization task can be done on the basis of both the top and bottom face halves. This is a difference in *instruction* but not necessarily a difference in the underlying process or strategy that participants employ in the task. For instance, the contrast category can be processed on the basis of selectively attending to one of the face halves. The benefit of our SFT approach is that we can tell apart these different strategies. Ultimately, we would like a theoretical account of processing that applies across different decision tasks and can identify differences inherent in how stimuli of different types are processed. If composite faces are pooled into a single source, then we should have observed this as coactivity - we did not find any evidence of coactivity. Instead, we conclude that composite face stimuli are not processed holistically according to the strongest holistic notion of coactivity. Instead, the consistent findings of a mixture of serial and parallel processing across the face conditions suggest either a weaker form of holism or that the composite faces are processed as separable stimuli.

## Supplementary Information

Below is the link to the electronic supplementary material.

Supplementary file 1 (pdf 3126 KB)

## Data Availability

Data and materials are available from: https://github.com/knowlabUnimelb/SCHEMATICFACERULES.

## Appendix

**Table 9:** Prior Parameter Values and Transformations for the Normal distribution priors used in DE-MCMC Sampling

Parameter	Transformation	Prior Parameter	Values
$D_{lips}, D_{eyes}$	$\hat{D}=logit\{(D-x_0)/(x_1-x_0)\}$	$\mu =0$	$\sigma =.5$
$\sigma _{lips}, \sigma _{eyes}$	$\hat{\sigma }=\log (\sigma )$	$\mu =-1.5$	$\sigma =.2$
*A*	$\hat{A}=\log (A)$	$\mu =-1.05$	$\sigma =.2$
$b_A, b_B$	$\hat{b}=\log (b-A)$	$\mu =-1.05$	$\sigma =1$
*s*	$\hat{s}=\log (s)$	$\mu =-1.39$	$\sigma =.5$
$t_0$	$\hat{t}_0=\log (t_0)$	$\mu =-1.51$	$\sigma =.2$
$p_{X}$	$\hat{p}_x=logit(p_x)$	$\mu =0$	$\sigma =2$
$p_{Serial}$	$\hat{p}_{serial}=logit(p_{serial})$	$\mu =0$	$\sigma =2$
*m*	$\hat{m}=\log (m)$	$\mu =0$	$\sigma =.2$
*v*	$\hat{v}_x=logit(v_x)$	$\mu =0$	$\sigma =1$

*Note.* Each parameter was transformed to the range $-\infty$ to $+\infty$. All parameters were then assigned Normal distribution priors

## Footnotes

1 The initial proofs of the MIC and SIC were formulated based on the assumption of error-free performance (Townsend & Nozawa, 1995). Little et al. (2022) showed that for the AND MIC and SIC, one can condition on any level of accuracy and, so long as the assumption of selective influence, independence, and stochastic dominance are preserved, the predictions are preserved. We assume that selective influence and independence hold and test for stochastic dominance in the target category by ensuring that $S_{LL}\left( t\right) \ge \left[ S_{LH}\left( t\right) ; S_{HL}\left( t\right) \right] \ge S_{HH}\left( t\right)$ with a strict inequality holding for at least one time point (Heathcote et al., 2010; Townsend, 1990b; Houpt et al., 2014).

2 We additionally examined the same effect in inverted faces. Face inversion is thought to disrupt holistic processing (Yin, 1969; Tanaka & Farah, 1993a; Scapinello & Yarmey, 1970), but the composite face interaction has been found with inverted faces, albeit with a smaller effect size and at a longer time scale (Richler et al., 2011a). To foreshadow, we did not find a composite task interaction with inverted faces (Experiment 1) nor did we find any evidence of coactivity; consequently, we report these results in our supplementary material.

3 We note that there may be concern that the representation derived from the MDS analysis of our online participants in Experiment 3 may not match the representation of the observers in Experiment 2. Following relevant precedent (Blunden et al., 2015; Cheng et al., 2018; Little et al., 2016; Moneer et al., 2016; Nosofsky et al., 2011; Osth et al., 2023), we note that we adopt the MDS coordinates as mean item locations in our computational modeling for convenience and not as a strong assumption about the underlying representation assumed by our observers. The mean locations ensure that the drift rate estimates derived from our model are identifiable. Note that the exact same drift rates can be predicted if we allow for other model parameters to scale appropriately. To be certain that the models were not being overly constrained by using the MDS coordinates for the mean item locations, we additionally tested a highly flexible free drift model which is not constrained by the MDS results.

## Code availability

Code is available from: https://github.com/knowlabUnimelb/SCHEMATICFACERULES.

## Author note

Portions of this paper were completed as part of partial fulfillment of a PhD degree by Xue Jun Cheng. This work was supported by ARC Discovery Project Grant DP160102360 to Daniel R. Little and by an Australian Government Research Training Program Scholarship awarded to Xue Jun Cheng. Correspondence concerning this article should be addressed to Daniel R. Little, School of Psychological Sciences, University of Melbourne, Melbourne, Victoria 3010, Australia. E-mail: daniel.little@unimelb.edu.au.
