# Supplementary material for: Second-order facial features are processed analytically in composite faces
Source: Atten Percept Psychophys. 2025 Aug 29;87(8):2388–416. doi: 10.3758/s13414-025-03144-0 (PMC12568841; doi:10.3758/s13414-025-03144-0)
Supplement: Supplementary file 1 — (pdf 3126 KB) [file 13414_2025_3144_MOESM1_ESM.pdf]

Supplement: Second-order facial features are processed analytically in composite faces

Xue Jun Cheng[1], Daniel R. Little[1]
[1]The University of Melbourne, Melbourne, Australia

This supplement contains the results of a composite face task, logical rules double-factorial task, and a multidimensional scaling study applied to inverted schematic composite faces as used in Cheng and Little (2025). The posterior predictive distributions from the mixed serial-parallel model for both upright and inverted condition participants are also presented.

Composite Face Task

Results

For the inverted faces, there was a significant effect of alignment, $F(1,35) = 15.98$, $MSE = 0.13$, $p < .001$, $\eta_p^2 = .31$, but not for congruence, $p = .18$. Furthermore, the congruence $\times$ alignment interaction was not significant for the inverted faces, $F(1,35) = 0.98$, $MSE = 0.10$, $p = .33$, $\eta_p^2 = .03$.

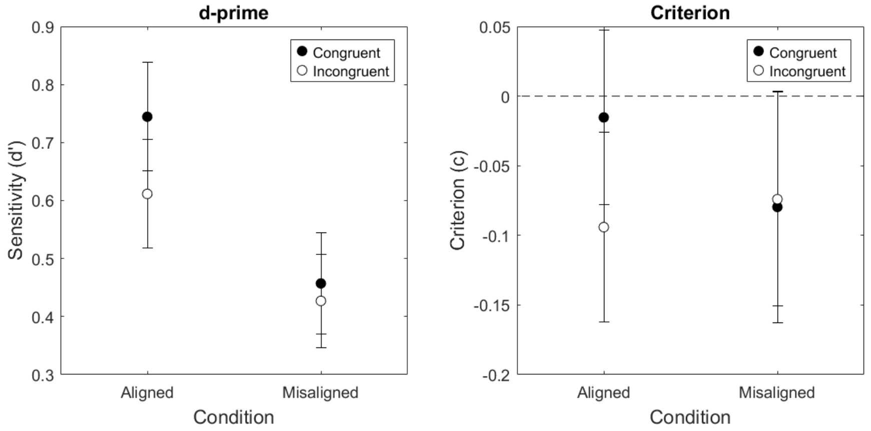

Figure 1. Sensitivity and criterion results (N=36) for Inverted Aligned and Inverted Misaligned Schematic Face conditions. Error bars represent standard errors.

As no interaction was found for the inverted faces, we conclude that inverting the faces disrupted holistic processing as it is measured by the composite face task.

None of the main effects or the interaction were significant for the criterion estimates in the inverted condition.

Experiment 2: Double Factorial Task

Method

Participants. Twelve participants from The University of Melbourne were assigned to one of the Inverted Aligned (N = 5) and Inverted Misaligned (N = 7) conditions. If a participant did not complete all eight sessions, they were removed from further analyses. This resulted in the removal of participants in the Inverted Aligned (N = 1) and Inverted Misaligned (N = 2) conditions, all of whom completed three or fewer sessions.

A total of 9 participants (8 female, 0 male) aged 20-30 ($M = 23$, $SD = 3.85$) remained.

Stimuli. The stimuli were the same as in the main text, but with the faces rotated by 180 degrees.

Results

For each participant, Session 1 was considered practice and removed from analyses. Trials with RTs that were less than 200 ms or greater than three times the standard deviation plus the mean for each item condition were excluded. This led to the removal of less than 2% of the trials.

Mean correct RTs, mean error RTs, and error rates for each participant are presented in Table 1. The SIC and MIC results are shown in Figure 6.

For the target category results, we ran a 7 (sessions 2-8) $\times$ 2 (top half: L or H) $\times$ 2 (bottom half: L or H) ANOVA on the target-category RTs for each participant (see Table 2). For the contrast category, we ran a series of planned *t* tests comparing the redundant stimulus to each of the other contrast category stimuli on each dimension as well as comparing the interior to the exterior stimuli on both dimensions (see Table 3).

All of the participants showed an effect of session (and, in some cases, an interaction between session and the top face half or session and the bottom face half, or both) indicating that response times decreased overall across session and that, in some cases, RTs to one or both of the dimensions decreased across sessions. Crucially, the three-way ANOVA was not significant for any observer indicating that the interaction between the top and bottom face halves did not change across sessions (see Table 3). As we can see from Table 4, for all observers in all four conditions, the HH RT distribution stochastically dominates the HL and LH distributions, which in turn stochastically dominate the LL RT distribution. This indicates that stochastic dominance is preserved and so we proceed with the SIC analyses for the target-category items.

We focus on the two primary diagnostic measures - the two-way interaction (MIC) between eye separation and lip height for the target category stimuli and the interior versus exterior comparison for the contrast category stimuli - for each face condition. To summarize the diagnostic measures, a non-significant target category MIC near zero along with slower interior versus exterior contrast category items is consistent with serial processing. Parallel processing is consistent with a significant negative MIC coupled with a negative SIC and a non-significant difference in the mean RTs for interior versus exterior contrast category items. Lastly, a significant positive MIC along with a mostly positive SIC and faster mean RTs for interior compared to exterior items indicates coactive processing. Two observers showed a significant top $\times$ bottom interaction: IM2, and IM3.

The SIC and MIC results for the inverted conditions are shown in Figures 2 and 3. The SIC results for participants IA1, IA2, and IA3 are consistent with serial processing, while participant IA4 displays some characteristics indicative of coactive processing. However, the non-significant eye separation $\times$ lip height interactions for all four participants (see Table 2, right hand columns) support an inference of serial processing.

The contrast category results for participant IA1 show that the interior stimulus displays longer RTs compared to the exterior on the Eyes dimension, and no significant differ-

ence between interior and exterior items on the lip height dimension. This result is consistent with fixed-order serial self-terminating processing. Participants IA2 and IA3 showed no significant differences between the interior and exterior items on both dimensions consistent with parallel self-terminating processing. Finally, participant IA4 had longer RTs for the interior stimulus compared to the exterior stimulus on the Eyes dimension and no significant difference between interior and exterior items on the lip height dimension. This result is consistent with fixed-order serial self-terminating processing.

SIC results for participants IM1 suggests parallel processing (see Figure 3, right column), but the non-significant interaction between the eye separation and lip height dimensions is consistent with serial processing. SIC results for both participants IM2 and IM3 suggest coactive processing. This inference is further supported by the significant eye separation x lip height interaction, confirming that the MICs for both participants were positive (see Table 2, right hand columns). For participant IM3, there was no significant difference between the interior and exterior items on both dimensions. However, both dimensions are processed significantly slower than the redundant stimulus. SIC results for participant IM4 looks inconsistent with any of the canonical model predictions. However, there was no significant difference between the interior and exterior items in the contrast category, suggesting parallel self-terminating processing.

Discussion

There is no strong evidence of coactive processing in either inverted condition. We also did not find any evidence for exhaustive processing as the redundant stimulus in the contrast category was always processed as fast as or faster than the interior or exterior stimuli (save for participants IA4 and IM4 who had one dimension which was processed faster than the redundant stimulus). Most of the participants show results that resemble the predictions of parallel or serial processing architectures. In addition, some participants also exhibited predictions which seem to fall between the predictions of these two architectures.

Table 1

Observed Mean Correct and Error RTs (ms) and Error Rates for Individual Stimuli for Each Observer in the Inverted Face Conditions

		Item								
Observer	Variable	HH	HL	LH	LL	Ex	Ix	Ey	Iy	R
IA1	RT correct	848	988	1019	1166	978	1013	845	856	740
	RT error	1190	1094	1152	1192	1014	1158	1257	1465	1012
	p(error)	.003	.03	.03	.08	.02	.02	.05	.02	.01
IA2	RT correct	822	1052	1027	1238	926	933	943	964	783
	RT error	-	1191	1665	1572	1155	1738	1618	2562	-
	p(error)	-	.006	.009	.02	.01	.01	.03	.006	-
IA3	RT correct	1155	1333	1414	1637	1254	1213	1158	1155	967
	RT error	1185	1258	1419	1560	1192	1454	1558	1516	1153
	p(error)	.03	.06	.17	.26	.31	.16	.07	.03	.01
IA4	RT correct	1083	1297	1131	1353	1125	1219	952	916	999
	RT error	1490	1397	1921	1588	1691	1707	1390	1648	-
	p(error)	.003	.12	.04	.11	.12	.11	.02	.01	-
IM1	RT correct	1414	1715	1649	1832	1555	1549	1626	1471	1284
	RT error	2789	2430	2503	2207	2065	2272	2468	2476	1468
	p(error)	.03	.06	.11	.23	.20	.11	.06	.02	.01
IM2	RT correct	1149	1304	1332	1642	1043	1028	1403	1383	953
	RT error	-	1791	1886	1963	1332	1609	1968	1732	-
	p(error)	-	.01	.03	.06	.02	.003	.03	.009	-
IM3	RT correct	1267	1381	1302	1602	1332	1415	1407	1417	1188
	RT error	797	1878	2068	2021	1124	1712	1901	2199	1584
	p(error)	.009	.03	.03	.05	.02	.02	.07	.11	.003
IM4	RT correct	708	783	802	882	772	790	575	562	619
	RT error	811	738	590	756	945	936	720	865	860
	p(error)	.02	.04	.20	.19	.10	.05	.03	.03	.006

Note. IA = inverted aligned; IM = inverted misaligned; HH = high-high stimulus x_2y_2; HL = high-low stimulus x_2y_1; LH = low-high stimulus x_1y_2; LL = low-low stimulus x_1y_1; R = redundant stimulus x_0y_0; Ix and Iy denote the interior stimuli on the Lip Separation x_1y_0 and Eye Separation x_0y_1 dimensions respectively; Ex and Ey denote the exterior stimuli on the Lip Height x_2y_0 and Eye Separation x_0y_2 dimensions respectively.

Table 2

Target Category Statistical Results for Individual Participants in the Inverted Conditions

Variable	*df*	*F*	*p*	η_p^2	*df*	*F*	*p*	η_p^2
		IA1				IM1		
Session	6	7.19**	< .001	.03	6	55.79**	< .001	.21
Session x Top	6	0.91	.48	.004	6	4.16**	< .001	.02
Session x Bottom	6	1.56	.16	.007	6	2.91*	.008	.01
Top	1	118.52**	< .001	.08	1	98**	< .001	.07
Bottom	1	173.82**	< .001	.12	1	78.01**	< .001	.06
Top x Bottom	1	0.02	.90	<.001	1	3.01	.08	.002
Top x Bottom x Session	6	0.90	.50	.004	6	0.62	.71	.003
Error	1324				1227			
		IA2				IM2		
Session	6	54.76**	< .001	.19	6	18.50**	< .001	.08
Session x Top	6	3.54*	.002	.02	6	3.30*	.003	.01
Session x Bottom	6	6.25**	< .001	.03	6	2.49*	.02	.01
Top	1	325.64**	< .001	.19	1	97.04**	< .001	.07
Bottom	1	258.16**	< .001	.16	1	126.08**	< .001	.09
Top x Bottom	1	0.28	.59	<.001	1	10.24*	.001	.008
Top x Bottom x Session	6	1.16*	.33	.005	6	1.46	.19	.006
Error	1366				1341			
		IA3				IM3		
Session	6	20.65**	< .001	.09	6	17.03**	< .001	.07
Session x Top	6	2.86*	.009	.01	6	1.15	.33	.005
Session x Bottom	6	2.19*	.04	.01	6	0.39	.89	.002
Top	1	49.81**	< .001	.04	1	51.67**	< .001	.04
Bottom	1	95.39**	< .001	.07	1	21.17**	< .001	.02
Top x Bottom	1	0.04	.84	<.001	1	10.34*	.001	.008
Top x Bottom x Session	6	0.57	.76	.003	6	0.48	.82	.002
Error	1200				1329			
		IA4				IM4		
Session	6	43.10**	< .001	.17	6	130.86**	< .001	.39
Session x Top	6	0.57	.75	.003	6	0.75	.61	.004
Session x Bottom	6	0.85	.53	.004	6	0.81	.56	.004
Top	1	84.31**	< .001	.06	1	57.06**	< .001	.04
Bottom	1	5.04*	.03	.004	1	61.83**	< .001	.05
Top x Bottom	1	0	.96	<.001	1	0.79	.37	<.001
Top x Bottom x Session	6	1.77	.10	.008	6	1.20	.30	.006
Error	1284				1215			

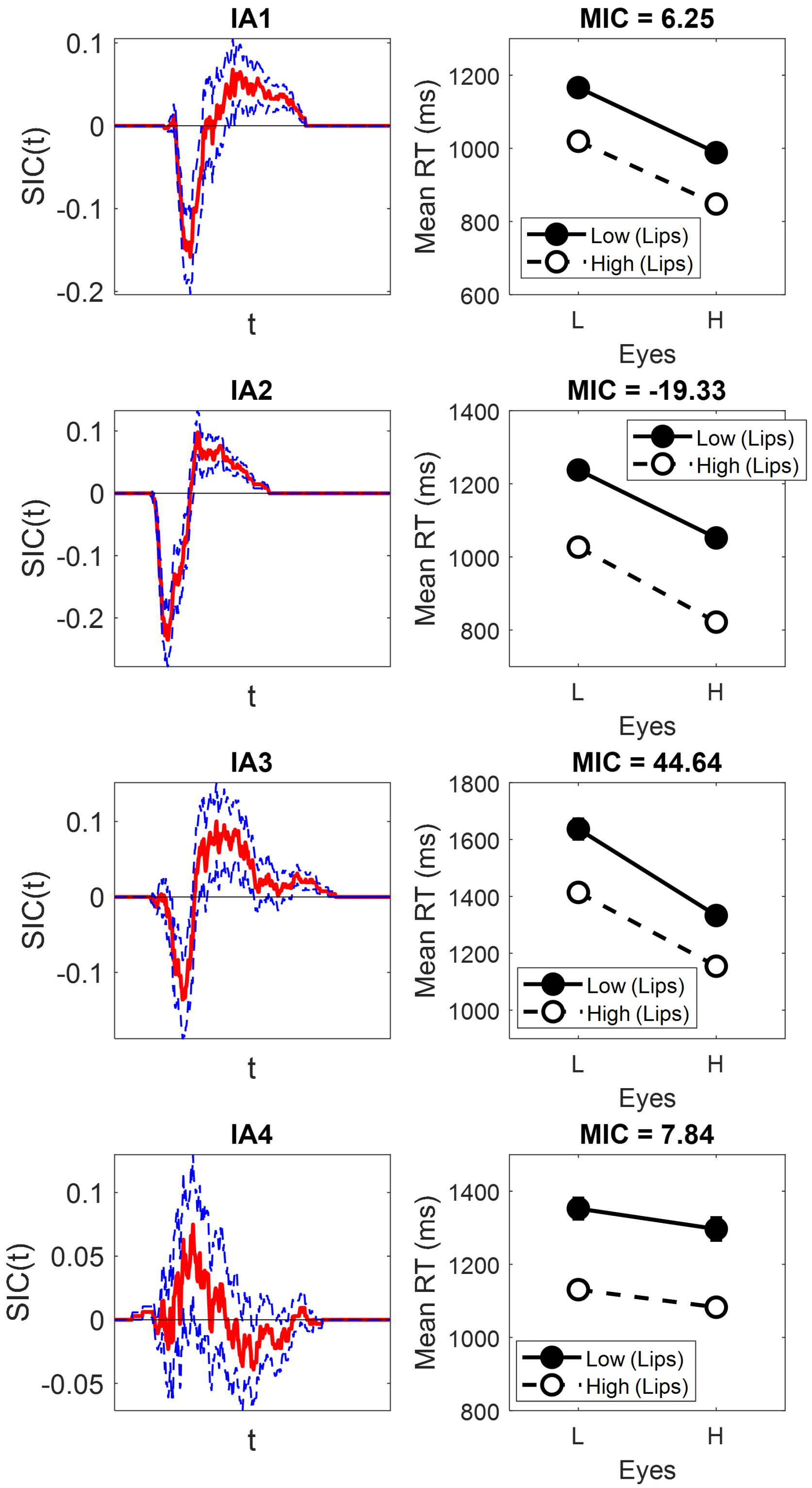

Figure 2. SIC and MIC results for the Inverted Aligned face conditions.

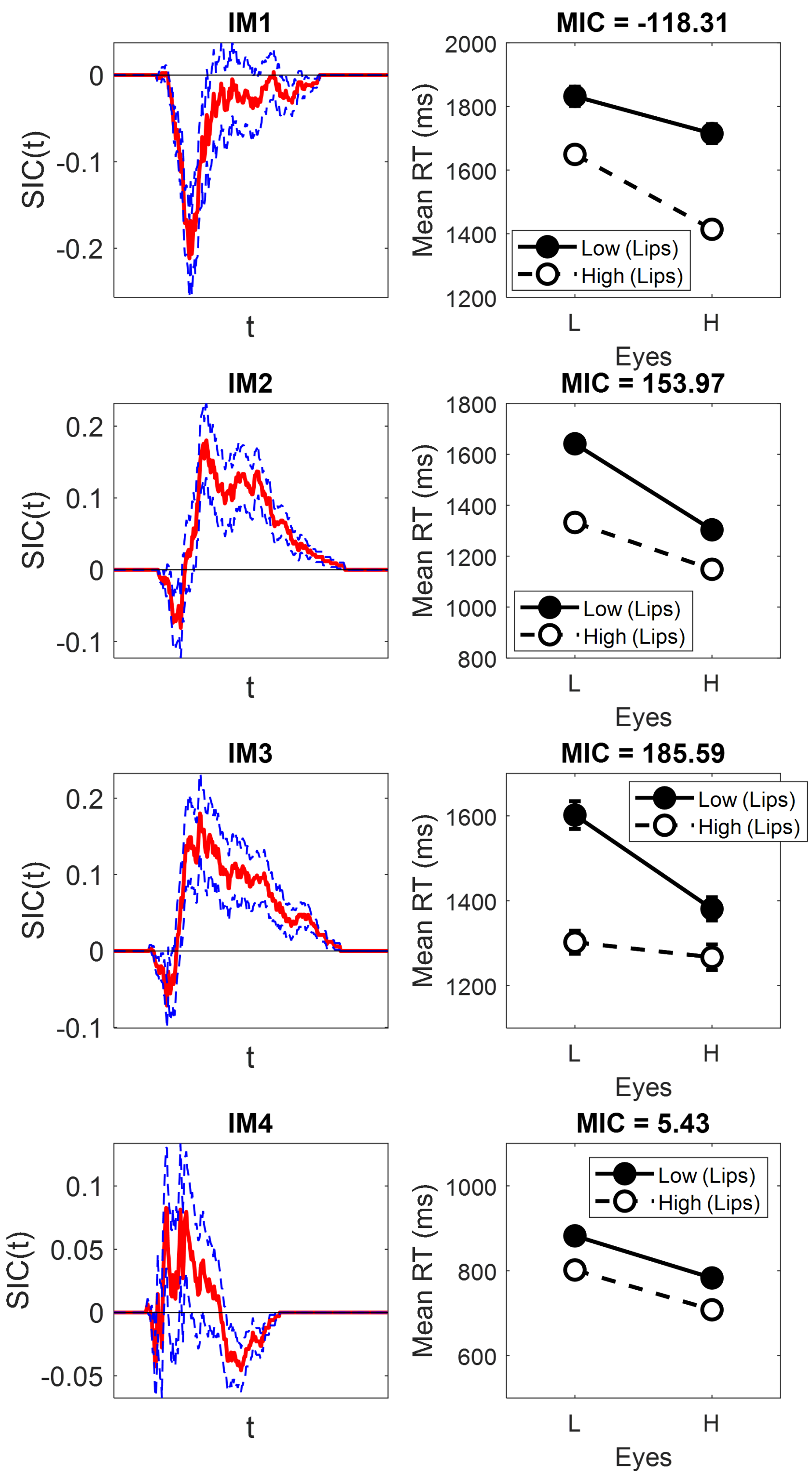

Figure 3. SIC and MIC results for the Inverted Misaligned face conditions.

Table 3

Contrast Category Statistics for Individual Participants in the Inverted Conditions

Variable	*M*	*t*	*df*	*p*	Cohen's *d*	*M*	*t*	*df*	*p*	Cohen's *d*
		IA1						IM1		
E_{Top} - I_{Top}	6	-2.08**	684	.04	0.16	6	0.18	591	.86	0.01
E_{Bottom} - I_{Bottom}	6	-0.58	676	.56	0.04	6	3.95**	666	< .001	0.30
E_{Top} - R	6	16.97**	688	< .001	1.29	6	9.36**	622	< .001	0.74
I_{Top} - R	6	18.24**	688	< .001	1.39	6	8.83**	655	< .001	0.68
E_{Bottom} - R	6	6.74**	680	< .001	0.51	6	9.77**	670	< .001	0.75
I_{Bottom} - R	6	7.10**	688	< .001	0.54	6	6.47**	682	< .001	0.49
		IA2						IM2		
E_{Top} - I_{Top}	6	-0.43	688	.67	0.03	6	0.45	694	.66	0.03
E_{Bottom} - I_{Bottom}	6	-0.96	686	.34	0.07	6	0.59	688	.56	0.04
E_{Top} - R	6	11.93**	691	< .001	0.91	6	2.92**	695	.004	0.22
I_{Top} - R	6	9.87**	693	< .001	0.75	6	2.36**	703	.02	0.18
E_{Bottom} - R	6	9.60**	684	< .001	0.73	6	13.28**	695	< .001	1.00
I_{Bottom} - R	6	11.03**	698	< .001	0.83	6	14.58**	697	< .001	1.10
		IA3						IM3		
E_{Top} - I_{Top}	6	0.95	533	.34	0.08	6	-1.77	685	.08	0.14
E_{Bottom} - I_{Bottom}	6	0.08	668	.93	0.01	6	-0.24	638	.81	0.02
E_{Top} - R	6	9.05**	579	< .001	0.74	6	3.71**	686	< .001	0.28
I_{Top} - R	6	7.32**	634	< .001	0.57	6	5.21**	689	< .001	0.40
E_{Bottom} - R	6	7.35**	667	< .001	0.57	6	5.75**	666	< .001	0.44
I_{Bottom} - R	6	7.07**	681	< .001	0.54	6	5.88**	662	< .001	0.46
		IA4						IM4		
E_{Top} - I_{Top}	6	-2.38**	627	.02	0.19	6	-1.00	649	.32	0.08
E_{Bottom} - I_{Bottom}	6	1.16	684	.25	0.09	6	1.22	680	.22	0.09
E_{Top} - R	6	3.44**	666	.001	0.27	6	10.13**	663	< .001	0.79
I_{Top} - R	6	5.50**	669	< .001	0.42	6	10.32**	684	< .001	0.79
E_{Bottom} - R	6	-1.30	697	.20	0.10	6	-3.39**	691	.001	0.26
I_{Bottom} - R	6	-2.49**	695	.01	0.19	6	-4.54**	687	< .001	0.35

Note. IA = inverted aligned; IM = inverted misaligned; E = exterior; I = interior; R = redundant. The subscript Top or Bottom refers to which dimension is varying in the comparison.

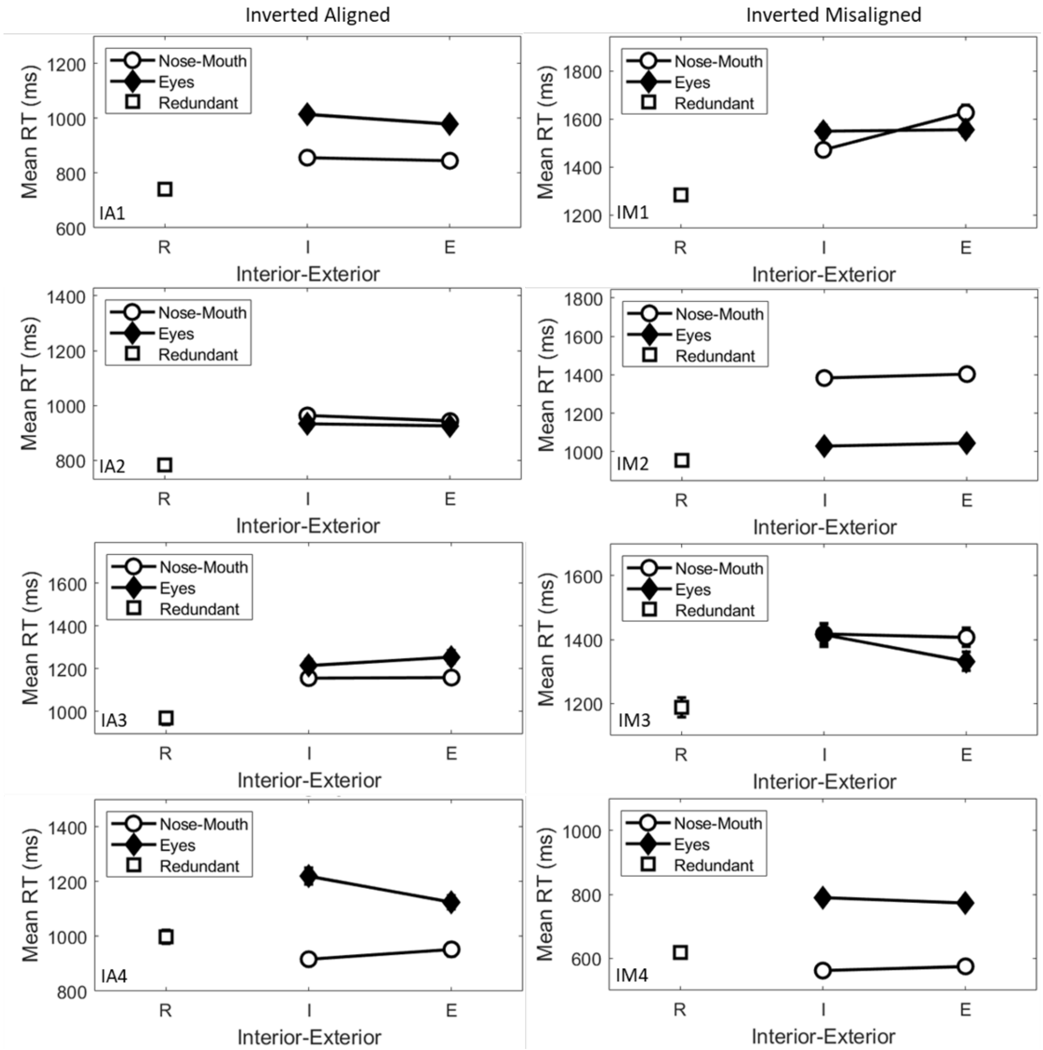

Figure 4. Observed contrast category mean RTs for the inverted aligned (left column) and inverted misaligned (right column) participants. Error bars represent ± 1 SE. Some of the standard error bars for the mean RTs are too small to be seen. The label "Nose-Mouth" refers to items which satisfy the category B rule on the lip height dimension (i.e., the vertical boundary) but vary on the Eyes dimension values. Correspondingly, the label "Eye" refers to items which satisfy the category B rule on the Eyes dimension (i.e., the horizontal boundary) but vary on the lip height dimension values.

Table 4
Stochastic Dominance Results for Each Observer in the Inverted Face Conditions

Test	Statistic	*p*	Statistic	*p*	Statistic	*p*	Statistic	*p*
	IA1		IA2		IA3		IA4	
$S_{HH} > S_{HL}$	0.35**	< .001	0.49**	< .001	0.25**	< .001	0.20**	< .001
$S_{HH} > S_{LH}$	0.31**	< .001	0.37**	< .001	0.29**	< .001	0.10**	<.05
$S_{HL} > S_{LL}$	0.26**	< .001	0.24**	< .001	0.27**	< .001	0.13**	<.05
$S_{LH} > S_{LL}$	0.26**	< .001	0.30**	< .001	0.17**	< .001	0.25**	< .001
$S_{HH} < S_{HL}$	0.00	> .99	0.00	> .99	0.003	> .99	0.009	.97
$S_{HH} < S_{LH}$	0.00	> .99	0.00	> .99	0.003	> .99	0.01	.93
$S_{HL} < S_{LL}$	0.00	> .99	0.003	> .99	0.009	.98	0.03	.79
$S_{LH} < S_{LL}$	0.00	> .99	0.00	> .99	0.011	.96	0.006	.99
	IM1		IM2		IM3		IM4	
$S_{HH} > S_{HL}$	0.35**	< .001	0.27**	< .001	0.21**	< .001	0.17**	< .001
$S_{HH} > S_{LH}$	0.33**	< .001	0.25**	< .001	0.14**	< .05	0.12**	< .05
$S_{HL} > S_{LL}$	0.19**	< .001	0.36**	< .001	0.19**	< .001	0.18**	< .001
$S_{LH} > S_{LL}$	0.21**	< .001	0.31**	< .001	0.30**	< .001	0.23**	< .001
$S_{HH} < S_{HL}$	0.03	.79	0.03	.75	0.01	.95	0.006	.99
$S_{HH} < S_{LH}$	0.009	.98	0.006	.99	0.02	.87	0.04	.62
$S_{HL} < S_{LL}$	0.01	.94	0.006	.99	0.00	> .99	0.02	.83
$S_{LH} < S_{LL}$	0.003	> .99	0.003	> .99	0.00	> .99	0.01	.96

Note. IA = inverted aligned; IM = inverted misaligned. If stochastic dominance is met, the first four tests should be statistically significant, while the last four tests should not.

Table 5
Statistical Significance of the Positive and Negative Parts of the SIC for Each Observer

Observer	D+	*p*	D-	*p*
IA1	0.06	.49	0.16	.01
IA2	0.11	.14	0.25	< .001
IA3	0.08	.31	0.15	.02
IA4	0.08	.31	0.03	.84
IM1	0.02	.91	0.20	< .001
IM2	0.16	.01	0.09	.22
IM3	0.16	.01	0.08	.29
IM4	0.11	.14	0.04	.79

Note. IA = inverted aligned; IM = inverted misaligned. D+ refers to the largest positive value of the SIC and D- refers to the largest negative value of the SIC.

Experiment 3: Multidimensional Scaling Study

Participants and Apparatus. Participants were randomly allocated to the inverted aligned (N = 21) or the inverted misaligned (N = 22) condition. All participants were recruited from the Amazon Mechanical Turk platform. Each participant took approximately 30 minutes and received $2 for completion of the task. Testing humans was approved by the University of Melbourne Human Research Committee 1340152.1.

Each experimental condition was programmed in Javascript and HTML and completed by the participant via a browser on their own computer.

Stimuli and Procedure. The procedure was the same as in the main text with the exception that at the beginning of the experiment, participants were sequentially presented with all nine faces in random order (e.g., either in an inverted aligned or inverted misaligned fashion, depending on the condition).

Data Analysis. Four participants were removed from the inverted aligned condition (updated N = 17), and ten were removed from the inverted misaligned conditions (updated N = 12) for not using the full response scale.

Results and Discussion

Table 6 shows the sum of squared deviations and Bayesian Information Criterion for each model. As shown in Figure 5, the MDS solutions for all both conditions align closely with our schematic distances settings. The Constrained Minkowski model (which allowed the r parameter to vary) fits best for both inverted aligned and misaligned face conditions. The estimated value of r was 2.82 in the inverted aligned condition and 3.26 in the inverted misaligned condition. Values of $r > 2$ indicate that the distance between the dimensions is influenced more by the largest distance on the top or bottom face half.

Computational modeling

See the main text for details about our computational modeling procedure.

Summary of Model Fits

For most participants, the most preferred model was the mixed serial-parallel model. This model was preferred for three participants inverted aligned and inverted misaligned conditions. For the remaining participants, the preferred model was the serial or mixed serial-contaminant model. The sole exception was participant IA2 who was best fit by the mixed parallel-contaminant model, but examination of the DIC weights (see Table 7; Wagenmakers and Farrell, 2004) reveals that the mixed serial-parallel model performed only marginally worse.

Figure 6 shows the 25, 50, and 75 percentiles from the Mixed Serial-Parallel model for the correct and mean RTs for each item and each participant.

Table 6

Summary of Sum of Squared Deviations and Bayesian Information Criterion for Each Model

		Full Model		
Condition	N	City block	Euclidean	Minkowski
IA	15	741.02 (365.93)	656.05 (300.16)	686.84 (331.22)
IM	10	528.51 (291.27)	461.42 (242.4)	461.8 (248.58)
		Constrained Model		
Condition	N	City block	Euclidean	Minkowski
IA	15	856.24 (368.66)	665.73 (232.57)	**651.92 (227.54)**
IM	10	578.43 (253.12)	462.09 (172.28)	**452.84 (170.89)**

Note. Cond = Condition, IA = Inverted Aligned, IM = Inverted Misaligned. BIC values are presented within parentheses.

Table 7

DICs (and DIC weights) for each model and each participant

		Model						
Condition	Participant	Serial ST	Mixed SC	Parallel ST	Mixed PC	Mixed SP	Coactive	Free Drift
IA	IA1	-288 (0)	-321 (0)	13 (0)	-32 (0)	**-376 (1)**	67 (0)	152 (0)
	IA2	-385 (0)	-508 (0)	-587 (0)	**-634 (.52)**	-633.8 (.48)	-459 (0)	-247 (0)
	IA3	5274 (0)	5270 (0)	5258 (0)	5266 (0)	**5168 (1)**	5325 (0)	5511 (0)
	IA4	4275 (0)	4265 (0)	4301 (0)	4316 (0)	**4092 (1)**	4209 (0)	4223 (0)
IM	IM1	5088 (0)	4989 (0)	4936 (0)	4966 (0)	**4884 (1)**	5084 (0)	5134 (0)
	IM2	**3624 (1)**	3641 (0)	3865 (0)	3894 (0)	3646 (0)	3806 (0)	4068 (0)
	IM3	5263 (0)	5270 (0)	5265 (0)	5273 (0)	**5103 (1)**	5247 (0)	5516 (0)
	IM4	-347 (0)	-340 (0)	-24 (0)	-19 (0)	**-365 (1)**	-71 (0)	177 (0)

Note. IA = Inverted Aligned, IM = Inverted Misaligned, ST = Self-terminating, SP = Serial-Parallel, SC = Serial Contaminant, PC = Parallel Contaminant. The lowest DIC model is bolded and italicized.

Table 8

Summary of Nonparametric and Parametric Results for Inverted Face Participants

Participant	SIC	MIC	Interior vs Exterior	Nonparametric Result	Best Parametric Model
IA1	S-shaped, neg. then pos.	Non-sig.	I > E Top	Parallel ST	Mixed S-P
IA2	S-shaped, neg. then pos.	Non-sig.	Non-sig.	Parallel ST	Mixed P-C
IA3	S-shaped, neg. then pos.	Non-sig.	Non-sig.	Serial ST	Mixed S-P
IA4	Mostly pos.	Non-sig.	I > E Top	Parallel ST	Mixed S-P
IM1	Negative	Non-sig.	I < E Bot	Parallel ST	Mixed S-P
IM2	S-shaped, more pos. than neg.	Sig. pos.	Non-sig.	Parallel ST/Serial ST	Serial ST
IM3	S-shaped, more pos. than neg.	Sig. pos.	Non-sig.	Coactive	Mixed S-P
IM4	S-shaped, pos. then neg.	Non-sig.	Non-sig.	Parallel ST/Serial ST	Mixed S-P

Note. sig = significant, non-sig = non-significant, pos = positive, neg = negative, ST = Self-terminating, I = interior, E = exterior, S-C = Serial-Contaminant, S-P = Serial-Parallel, Bot = Bottom. The Nonparametric Result is found by interpreting the SIC, MIC, and Interior vs Exterior results in that order. Where multiple interpretations are possible, all are listed.

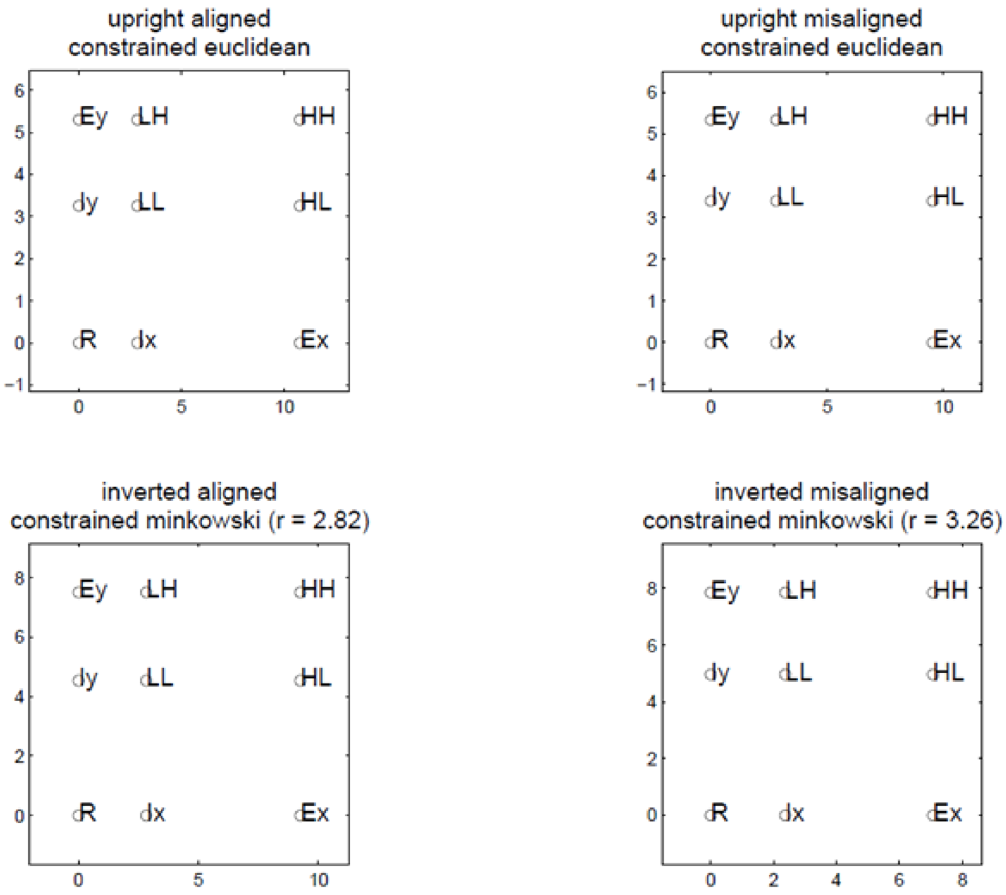

Figure 5. Best-fitting MDS solutions for each of the four face conditions. The r values for both Inverted Aligned and Inverted Misaligned face conditions are reported as Minkowski allows the r parameter to vary.

Discussion

For inverted faces, our modeling results show that processing was mostly consistent with a mixture of serial and parallel processing. This matches the inferences that were made for upright faces.

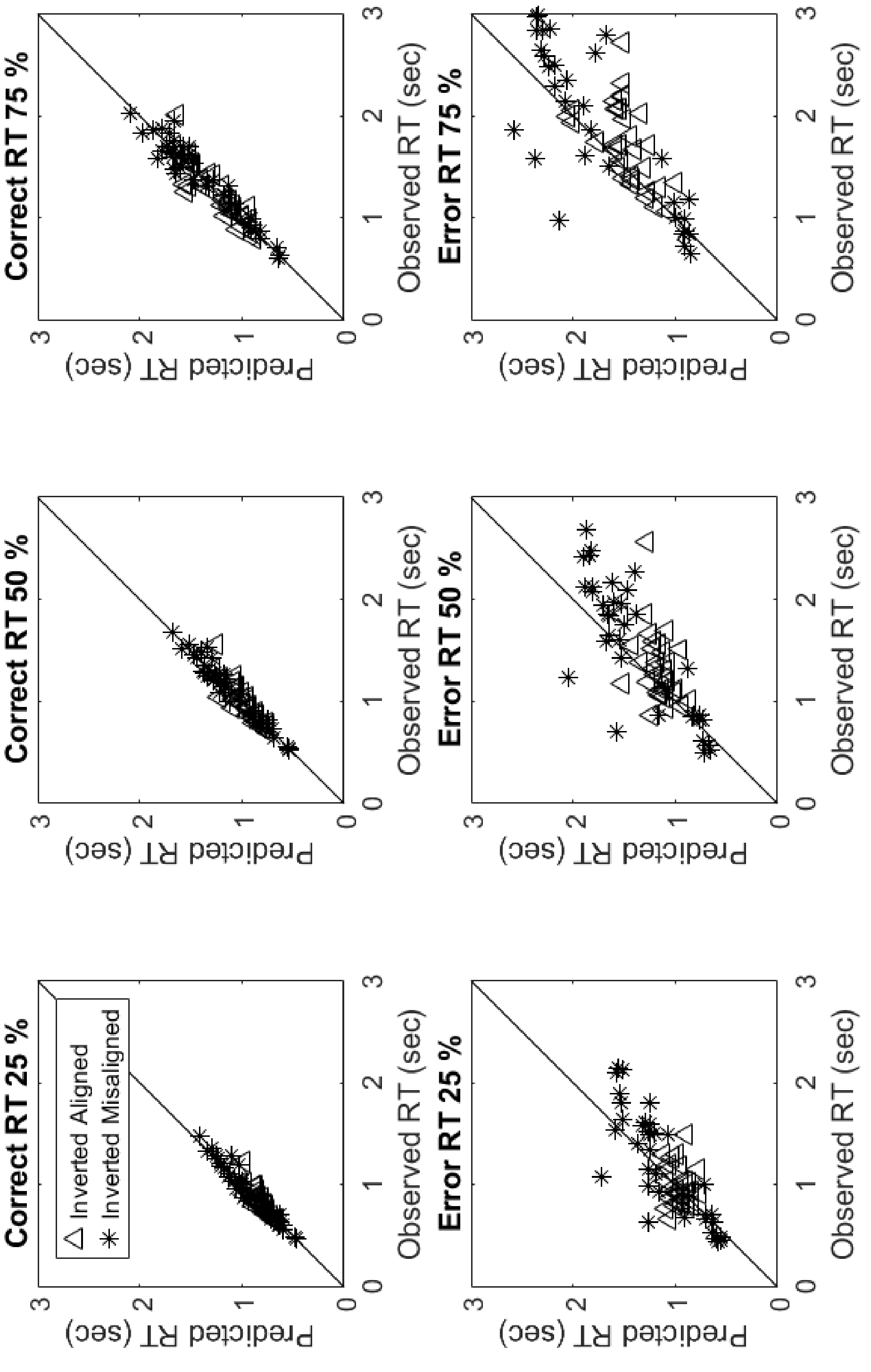

Figure 6. Posterior predictions of the Mixed Serial-Parallel model for the 25, 50, 75 tertiles for the correct and mean RTs for each item and each participant.

UA1: Mixed Serial-Parallel Model

Posterior Predictive Distributions

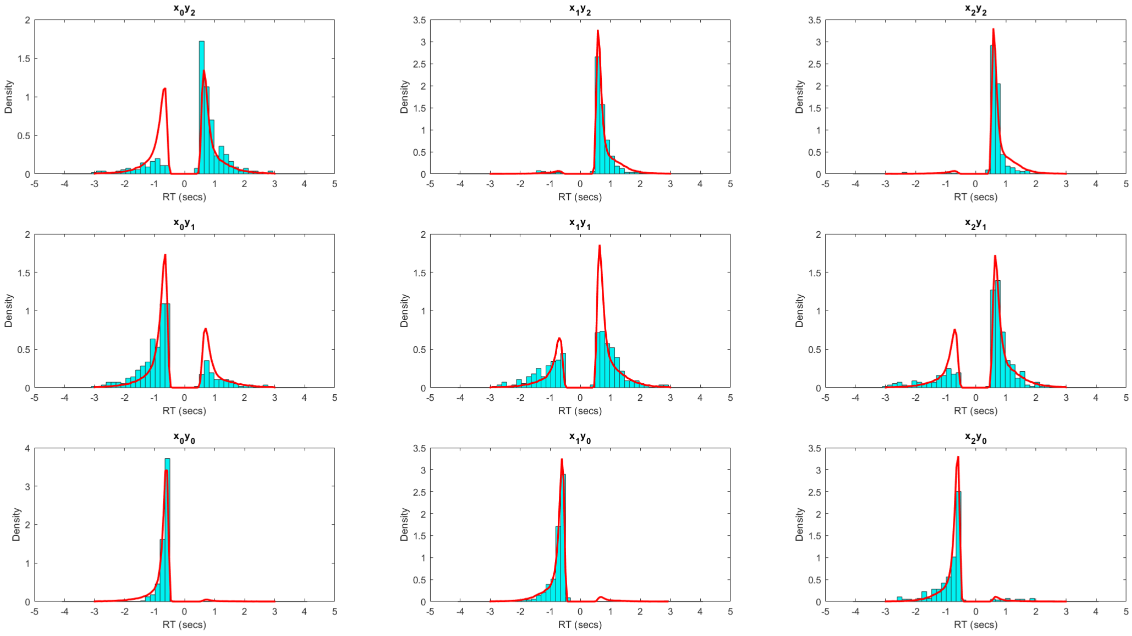

Posterior Parameter Distributions

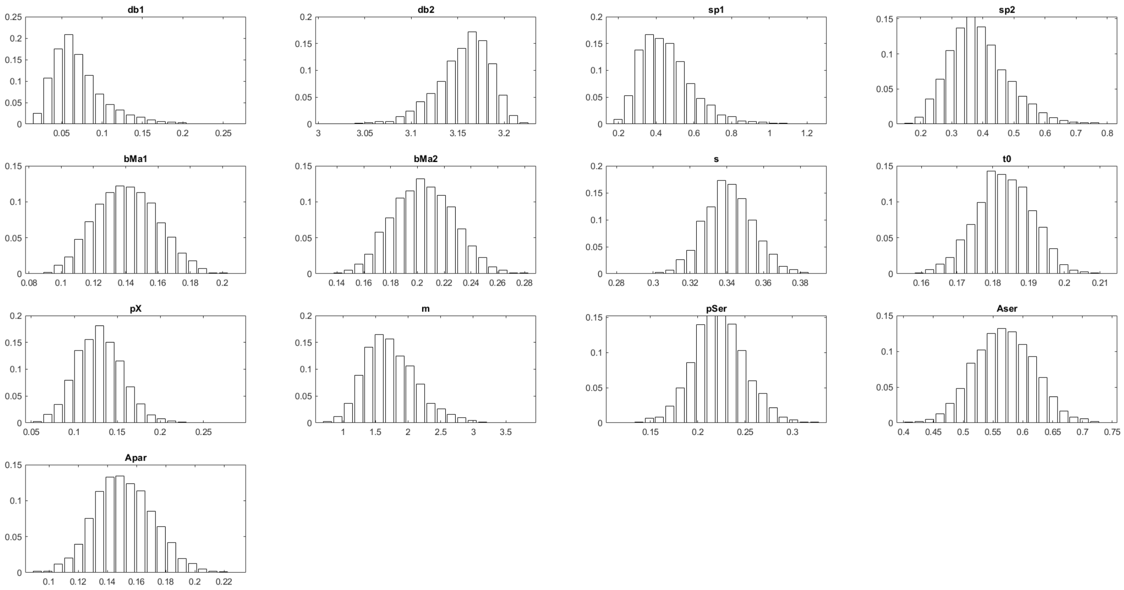

UA2: Mixed Serial-Parallel Model

Posterior Predictive Distributions

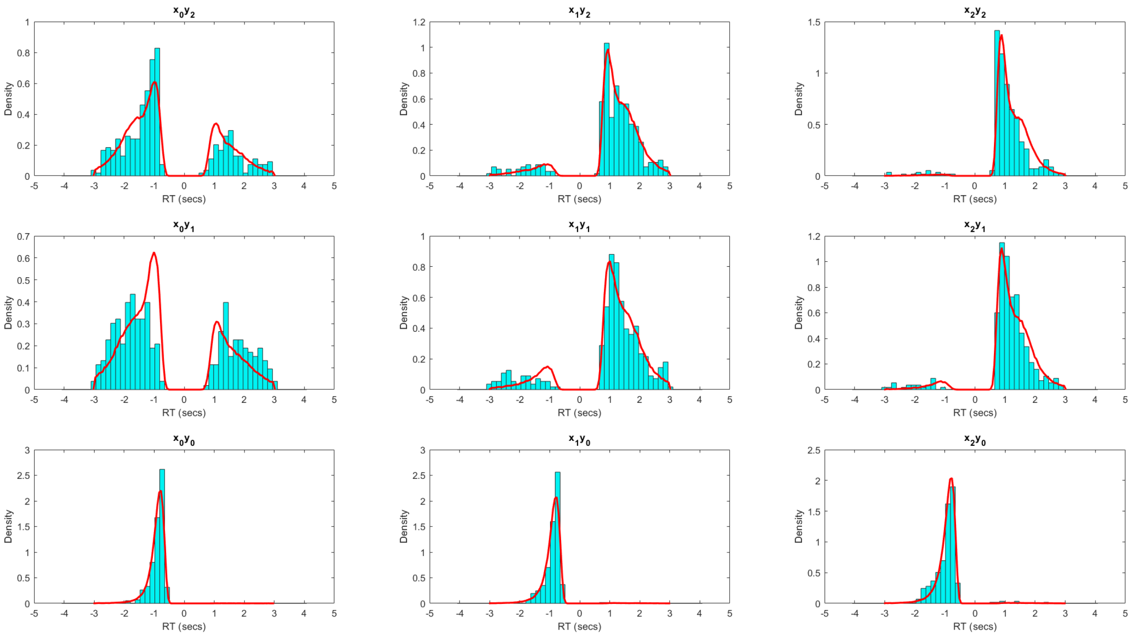

Posterior Parameter Distributions

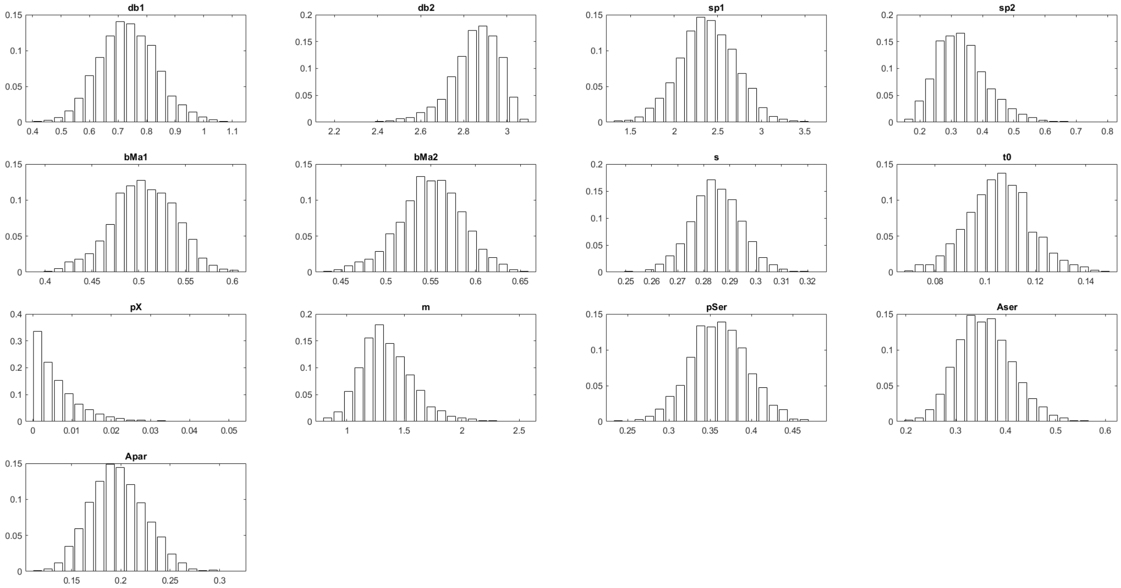

UA3: Mixed Serial-Parallel Model

Posterior Predictive Distributions

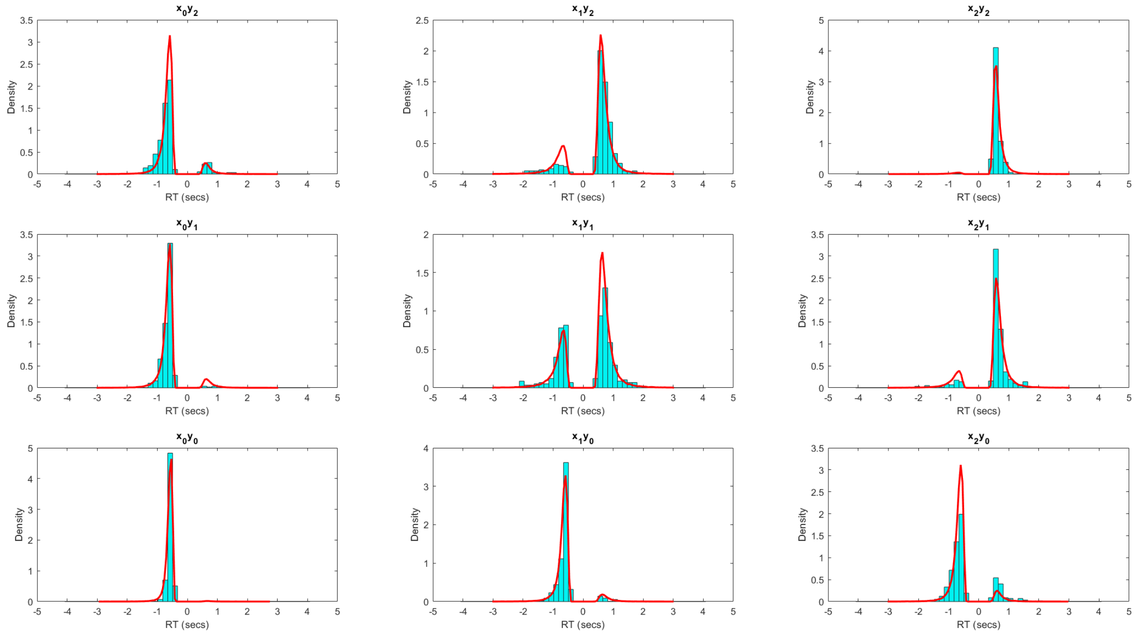

Posterior Parameter Distributions

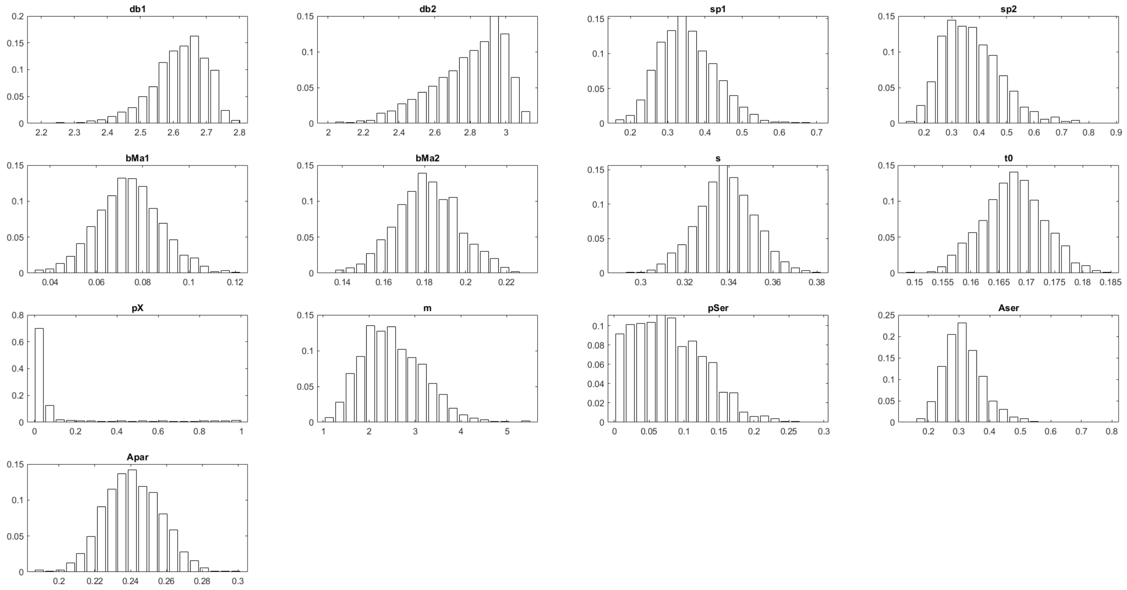

UA4: Mixed Serial-Contaminant Model

Posterior Predictive Distributions

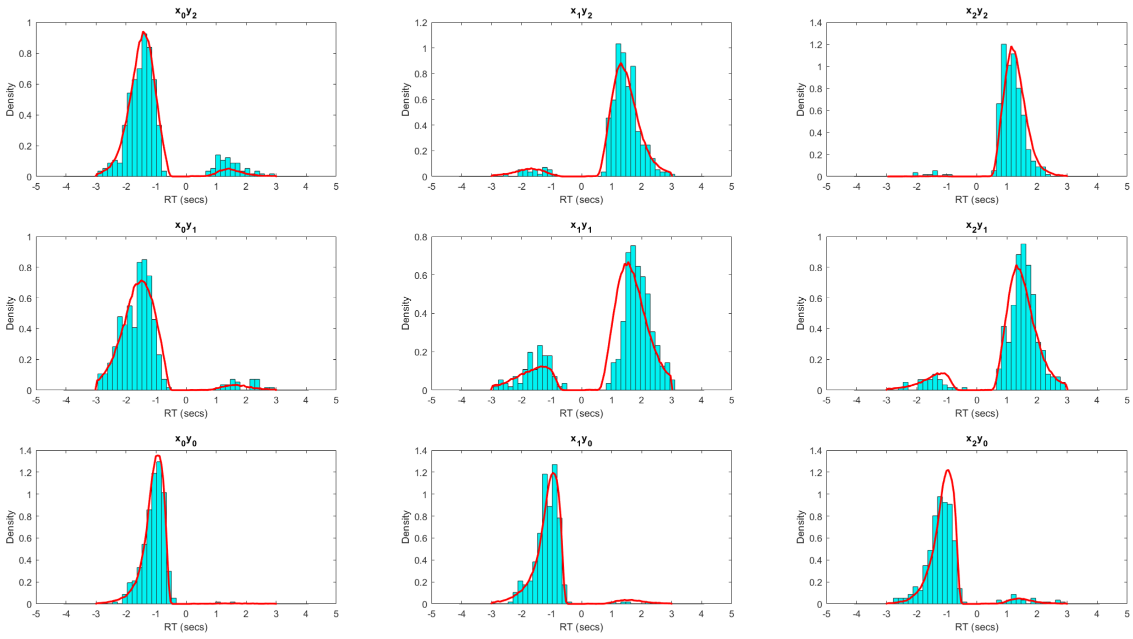

Posterior Parameter Distributions

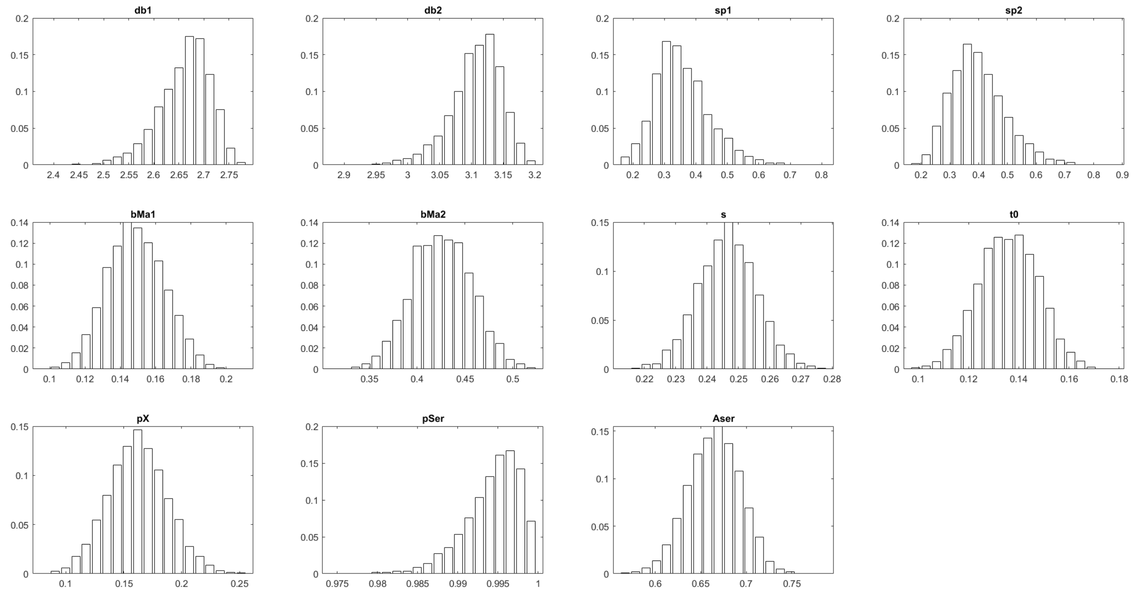

UM1: Mixed Serial-Contaminant Model

Posterior Predictive Distributions

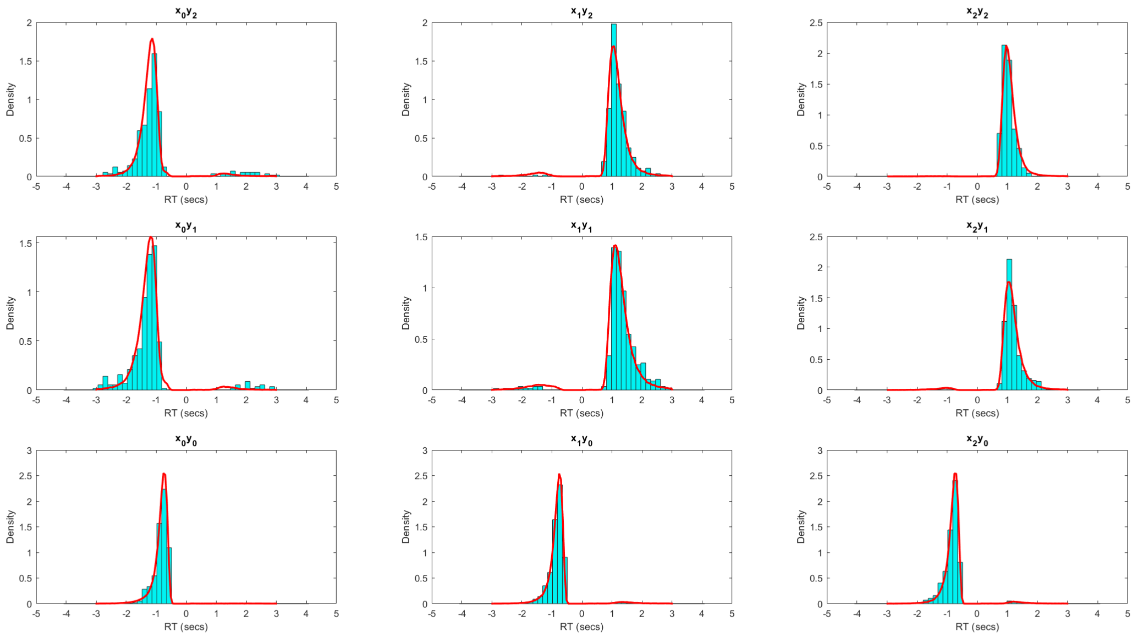

Posterior Parameter Distributions

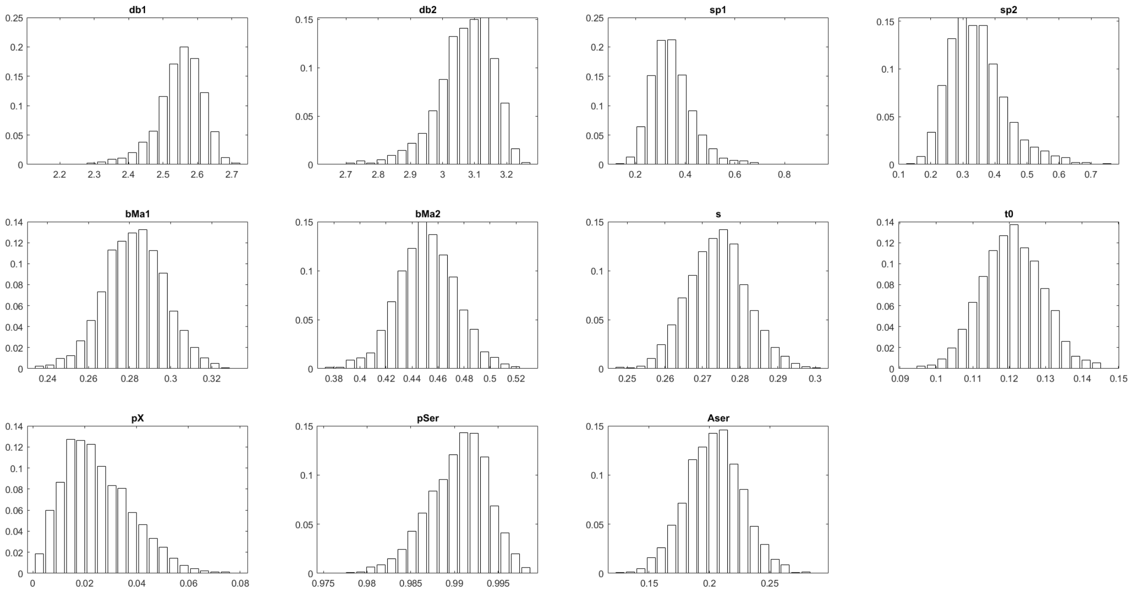

UM2: Mixed Serial-Parallel Model

Posterior Predictive Distributions

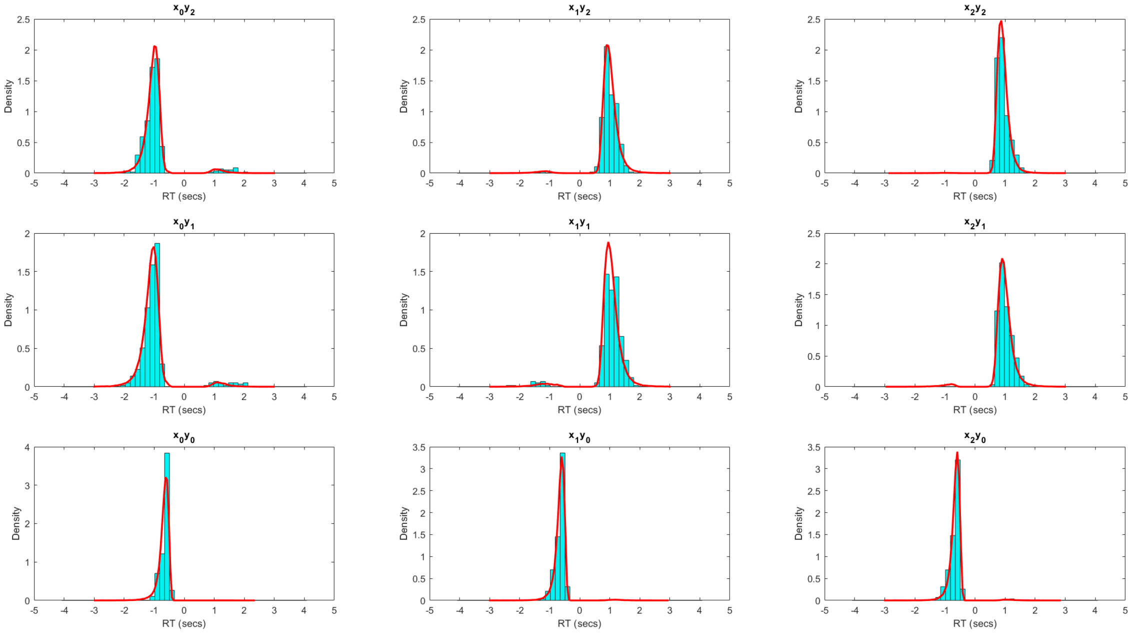

Posterior Parameter Distributions

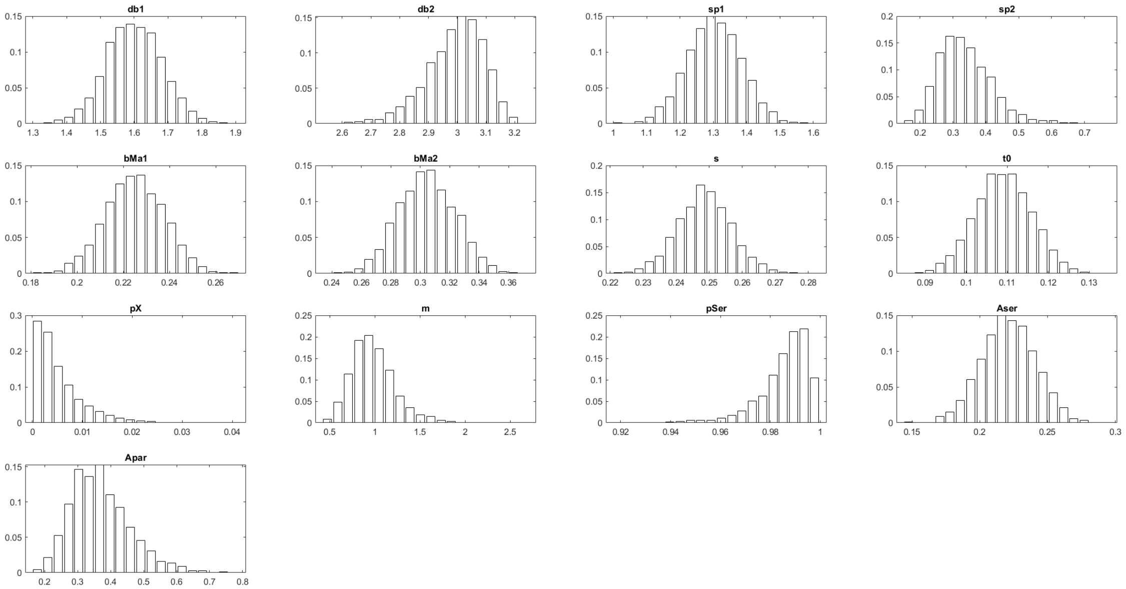

UM3: Mixed Serial-Contaminant Model

Posterior Predictive Distributions

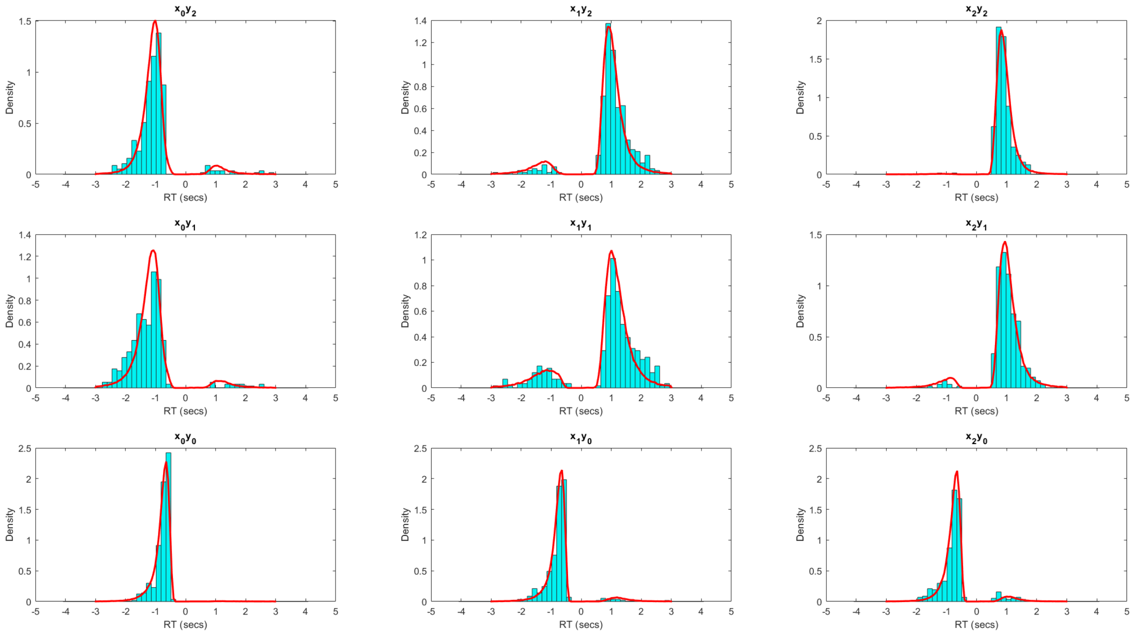

Posterior Parameter Distributions

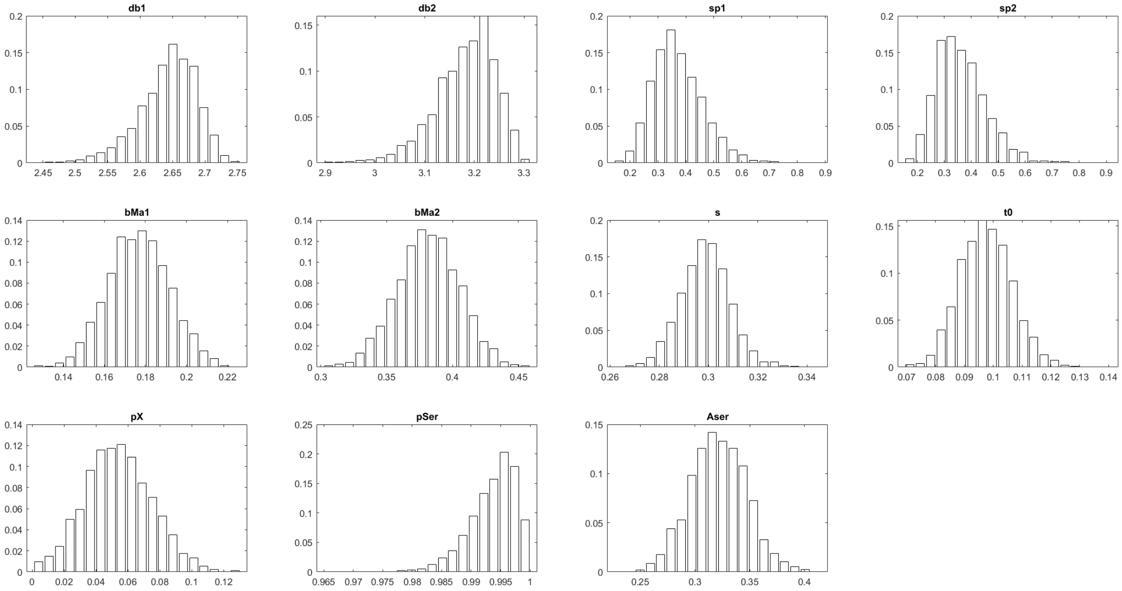

UM4: Mixed Serial-Parallel Model

Posterior Predictive Distributions

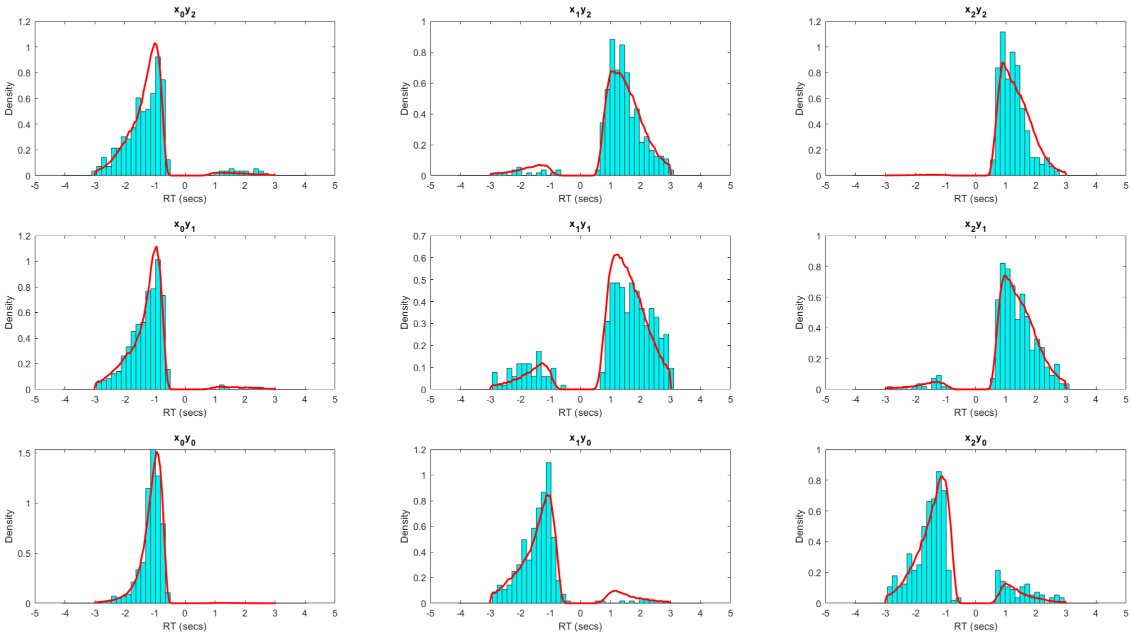

Posterior Parameter Distributions

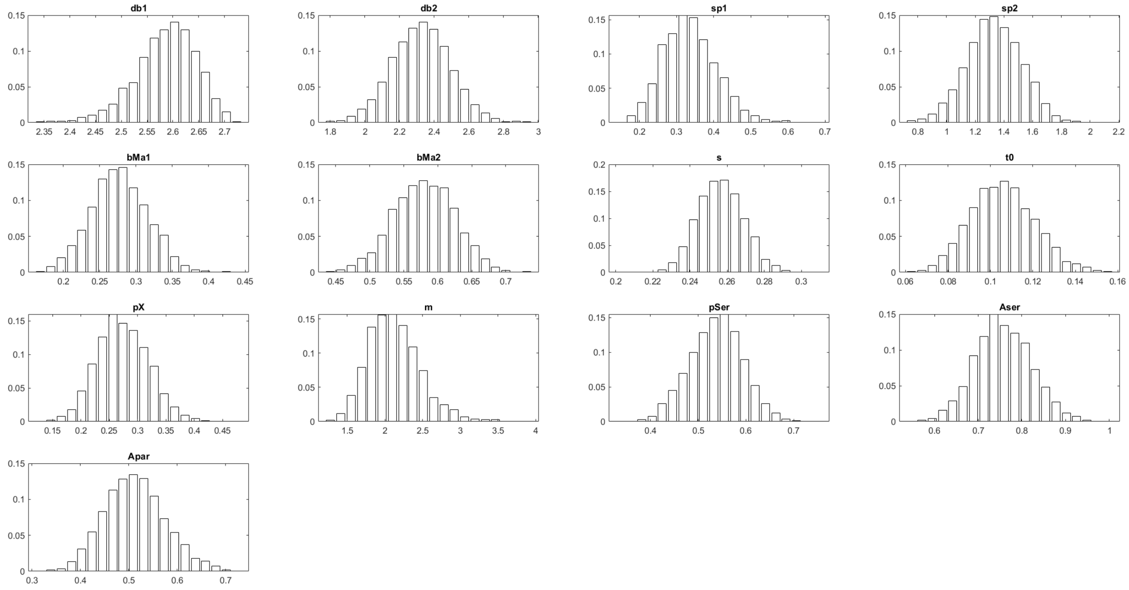

IA1: Mixed Serial-Parallel Model

Posterior Predictive Distributions

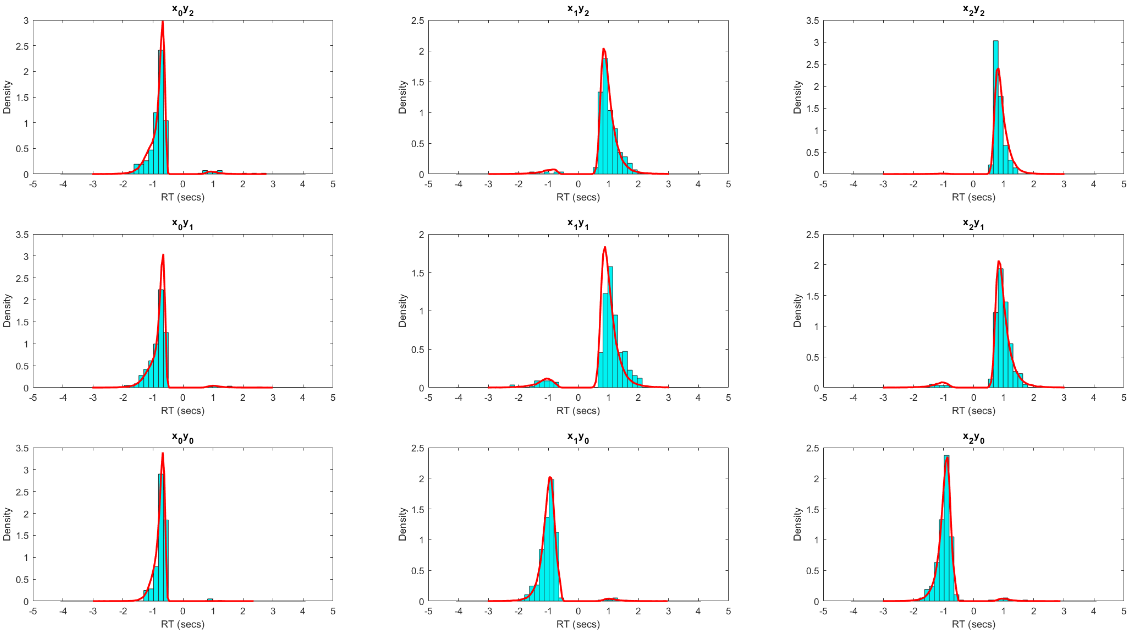

Posterior Parameter Distributions

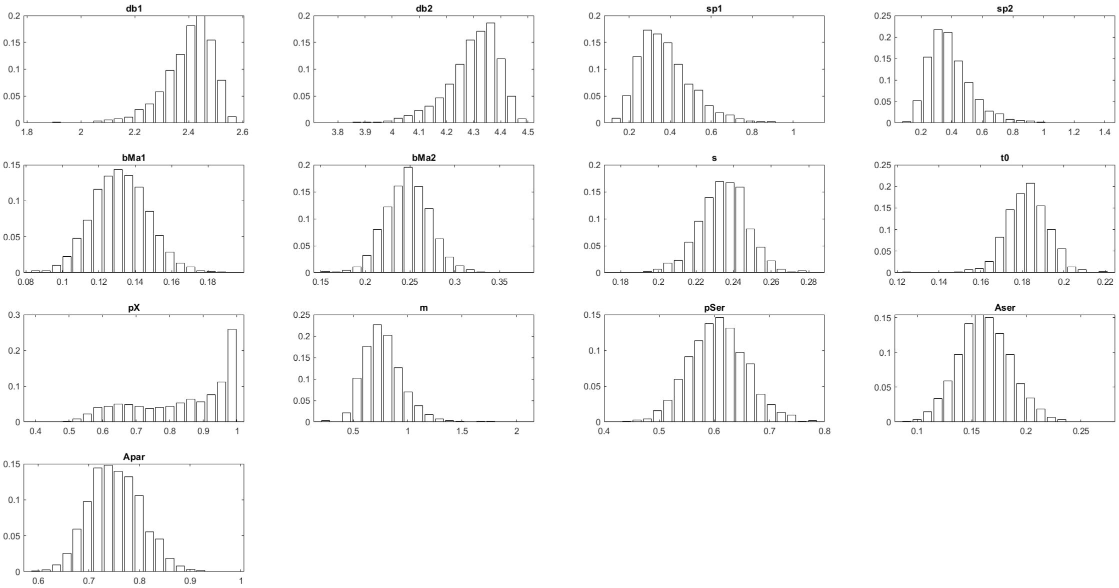

IA2: Mixed Serial-Contaminant Model

Posterior Predictive Distributions

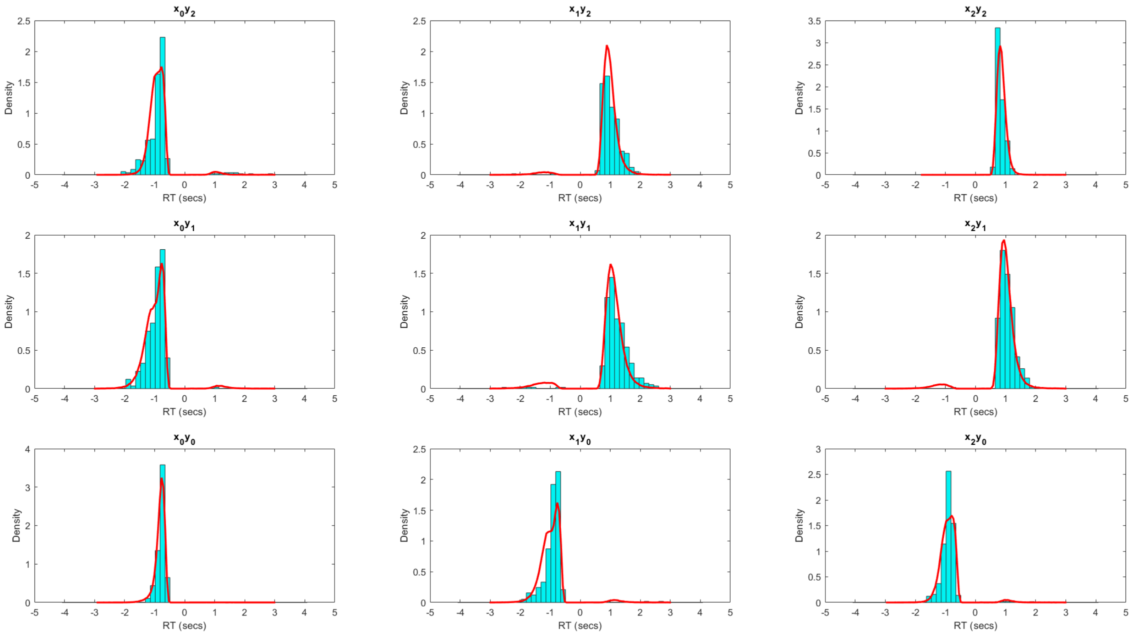

Posterior Parameter Distributions

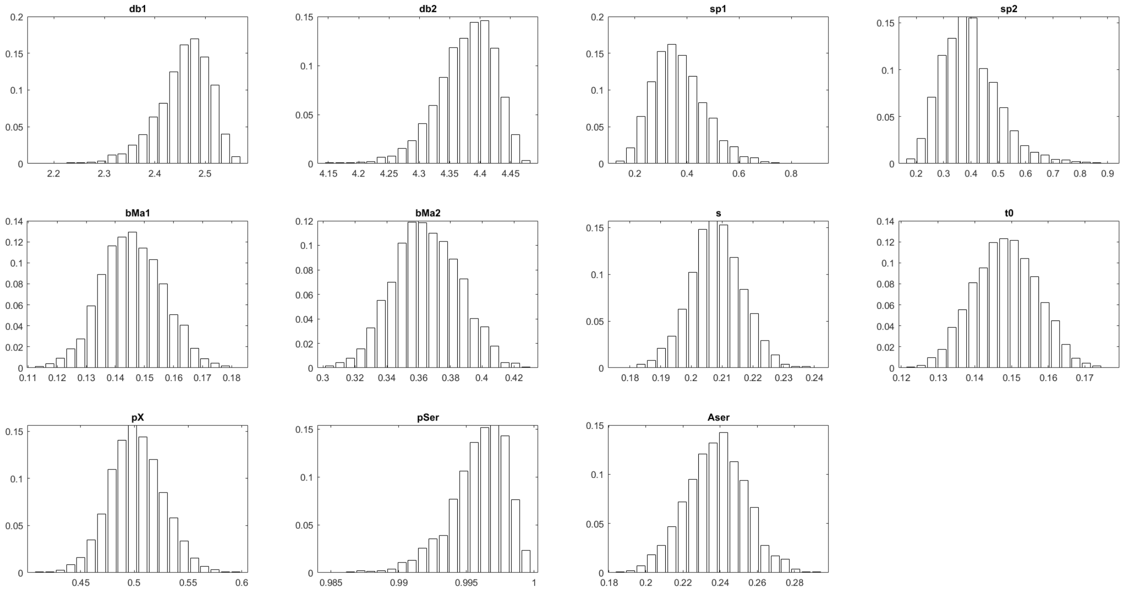

IA3: Mixed Serial-Parallel Model

Posterior Predictive Distributions

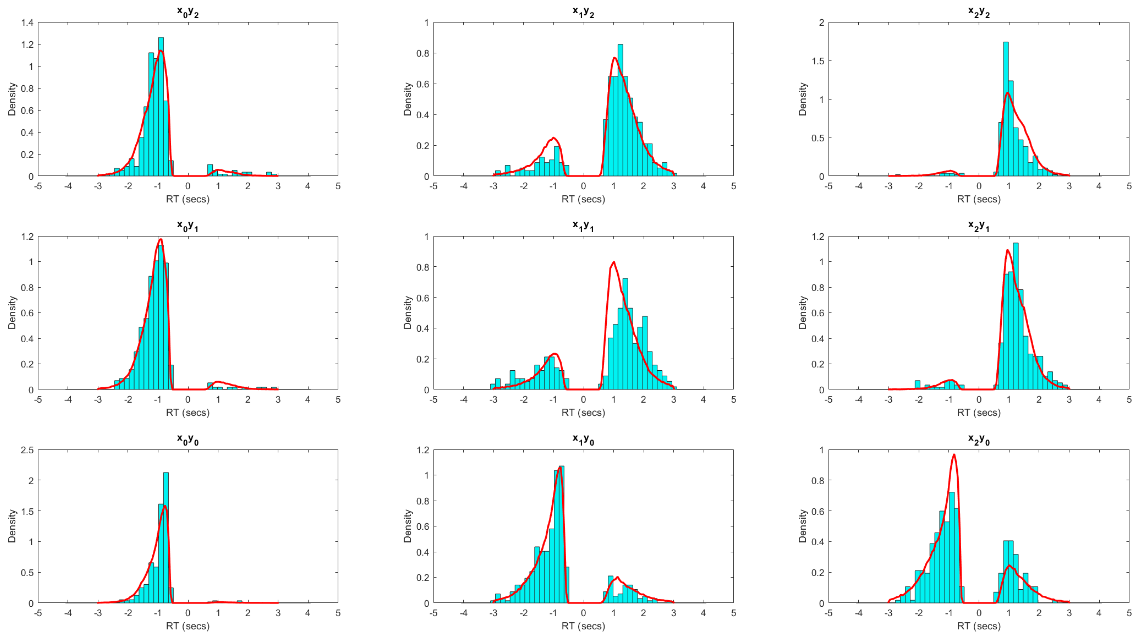

Posterior Parameter Distributions

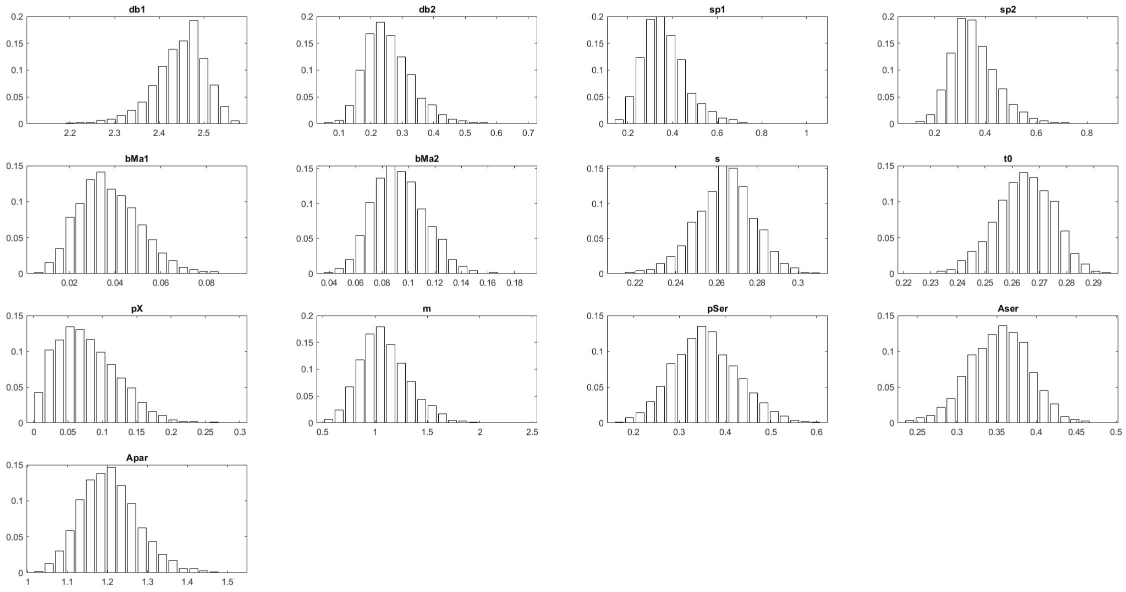

IA4: Mixed Serial-Parallel Model

Posterior Predictive Distributions

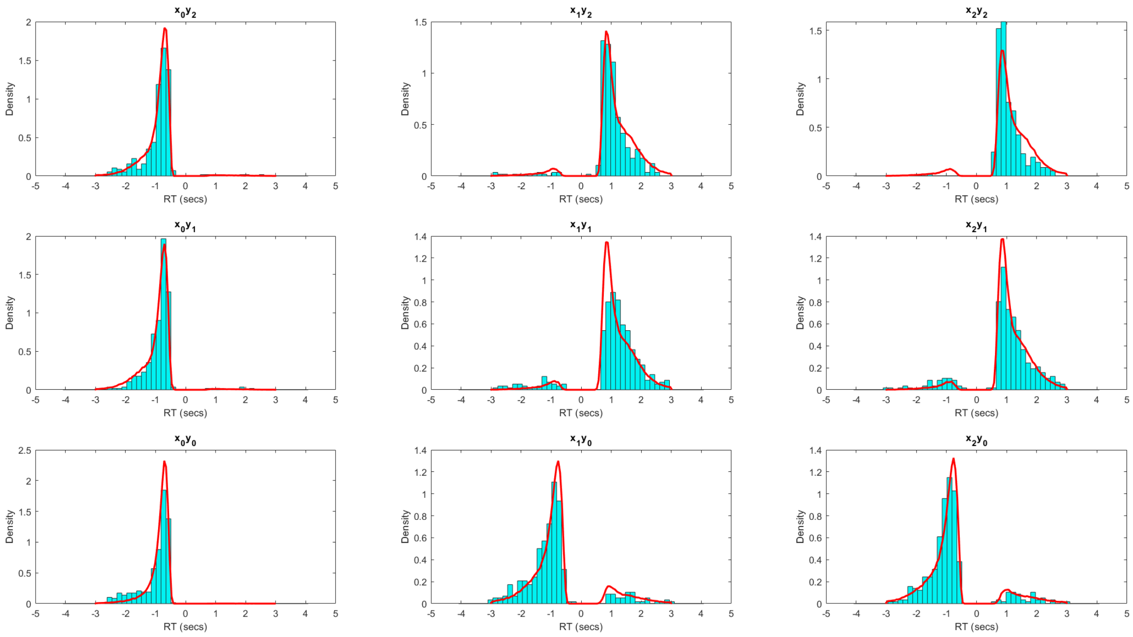

Posterior Parameter Distributions

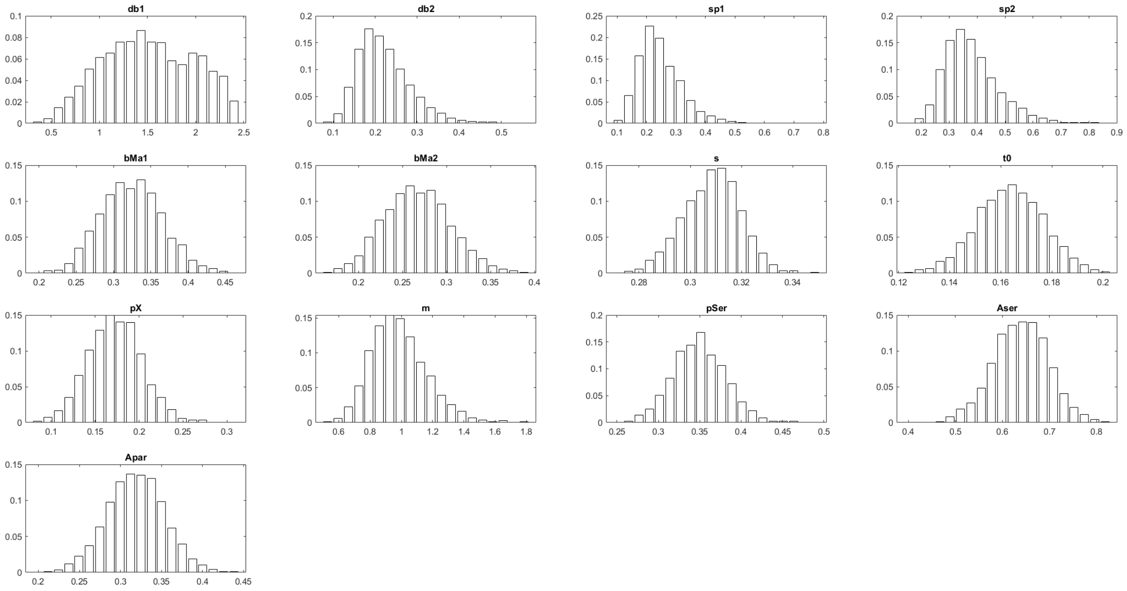

IM1: Mixed Serial-Parallel Model

Posterior Predictive Distributions

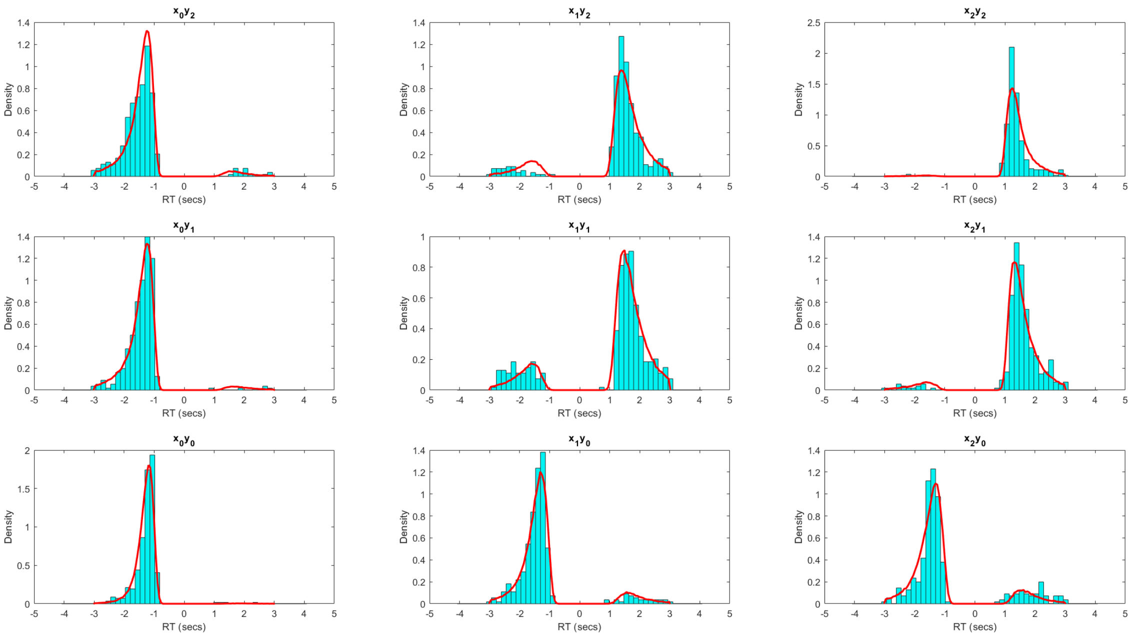

Posterior Parameter Distributions

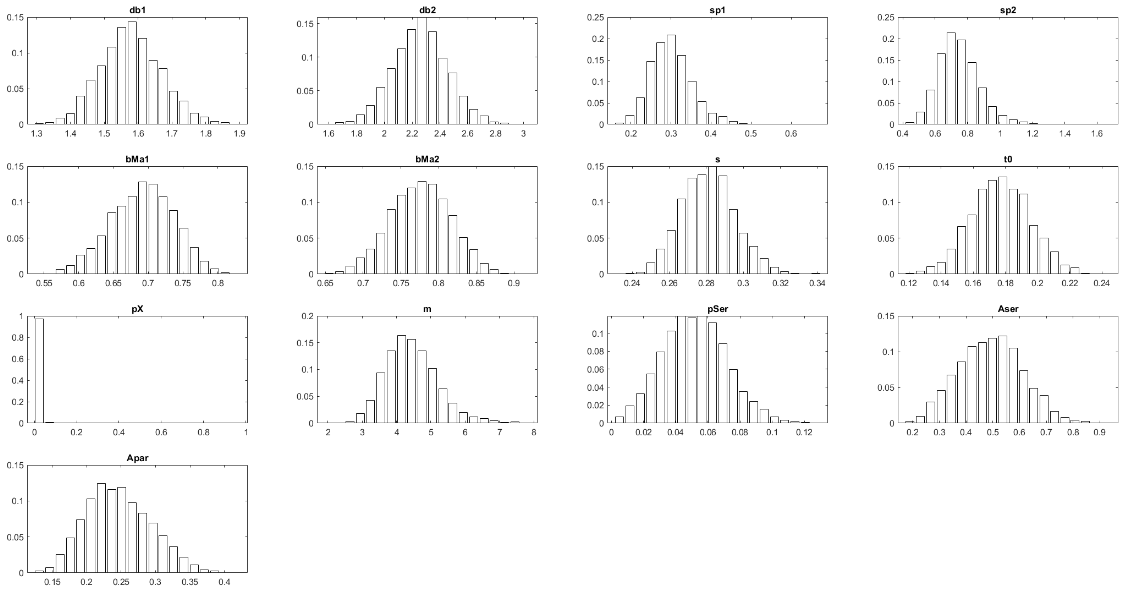

IM2: Serial Self-Terminating Model

Posterior Predictive Distributions

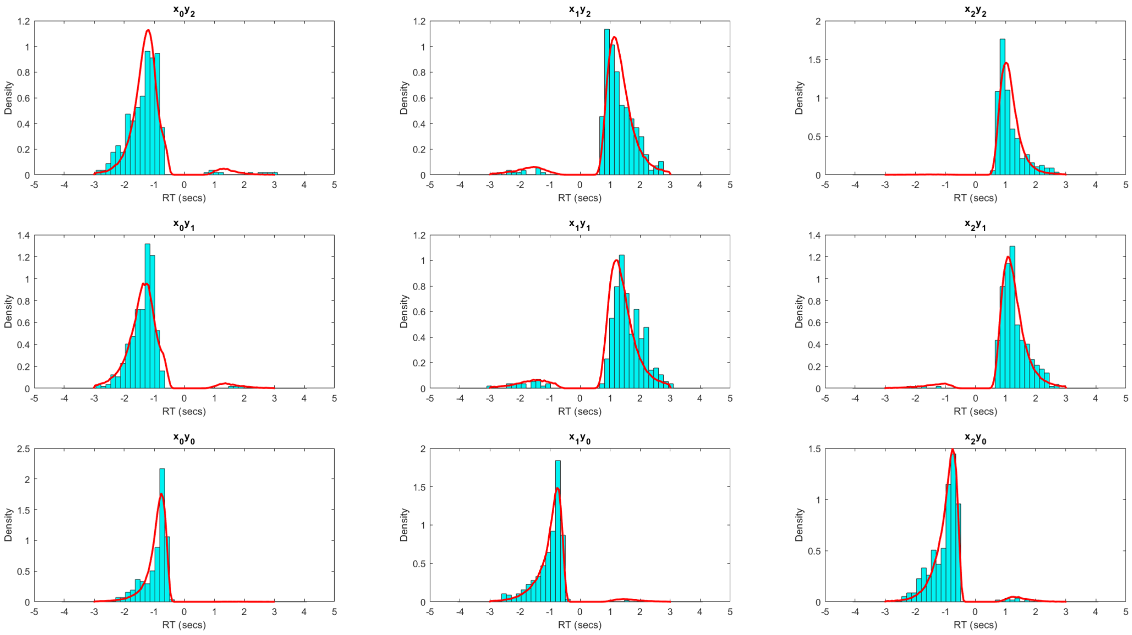

Posterior Parameter Distributions

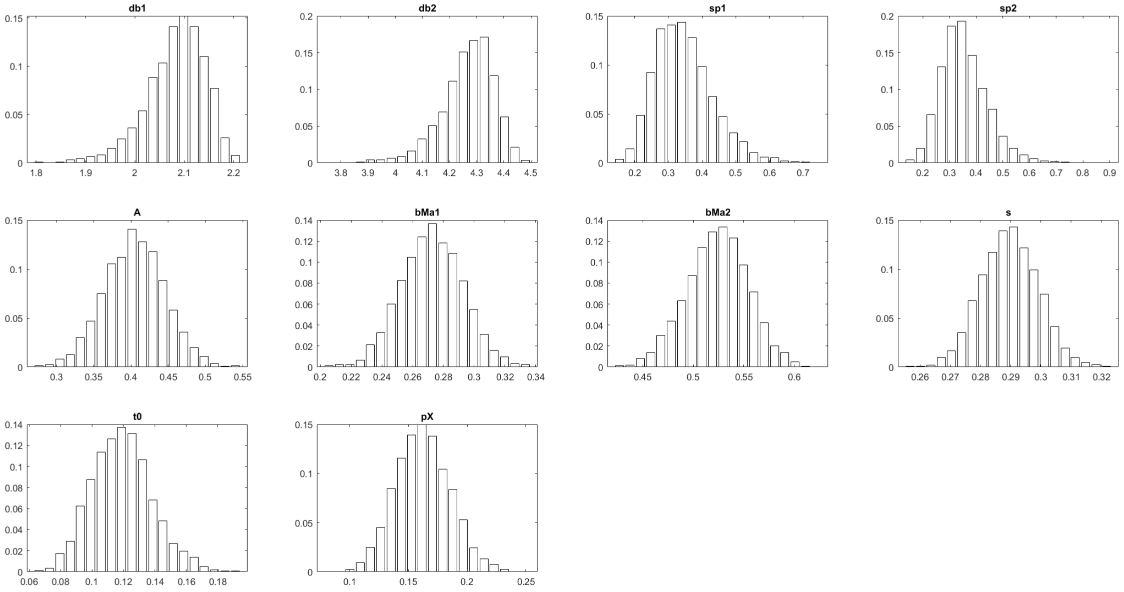

IM3: Mixed Serial-Parallel Model

Posterior Predictive Distributions

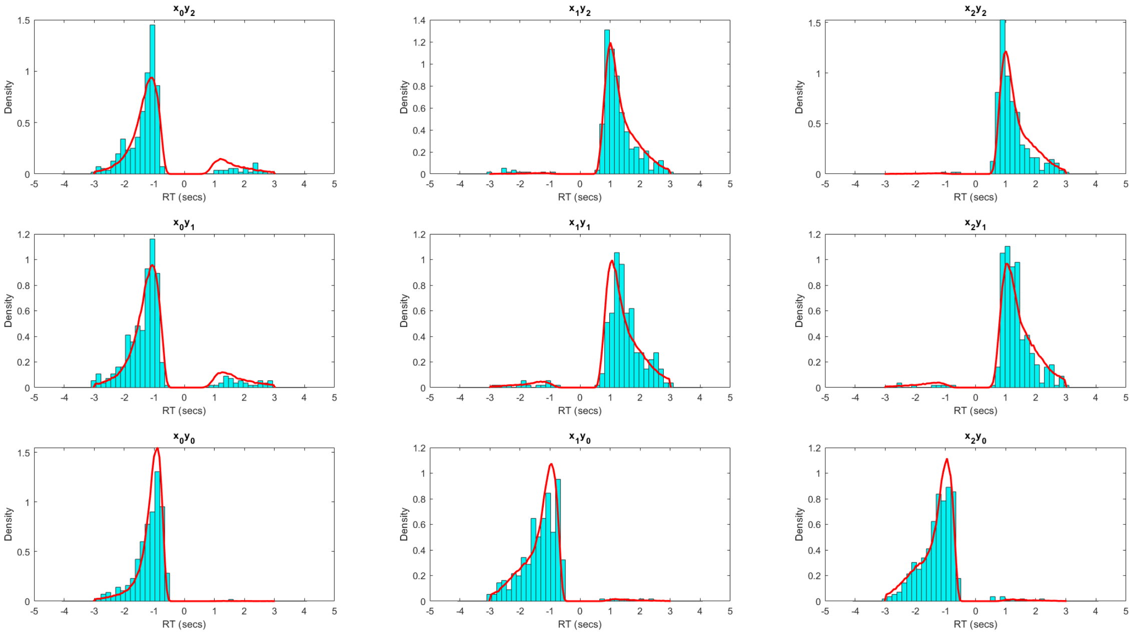

Posterior Parameter Distributions

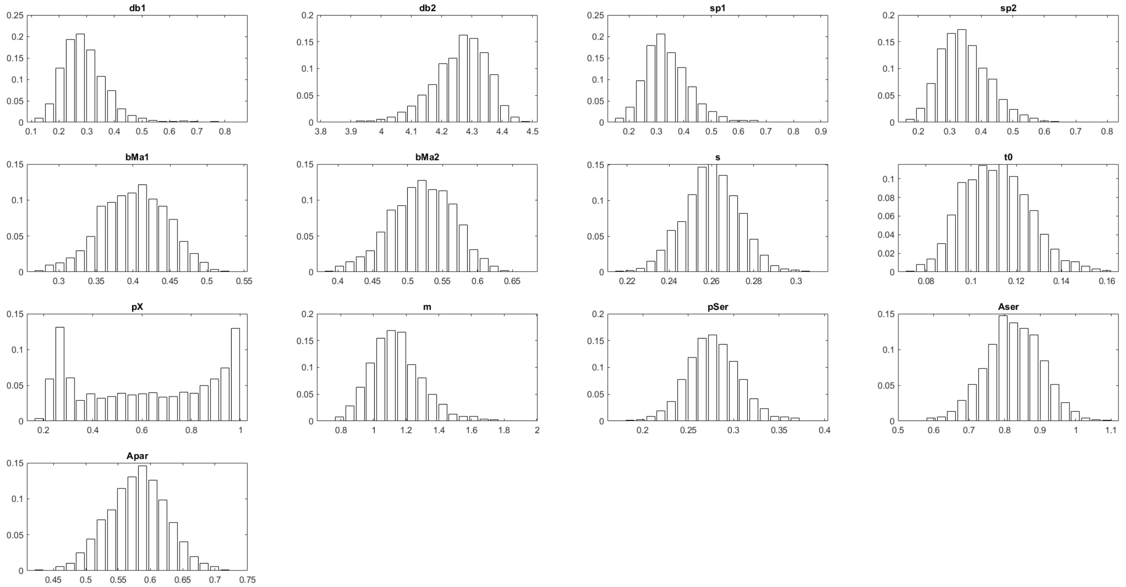

IM4: Mixed Serial-Parallel Model

Posterior Predictive Distributions

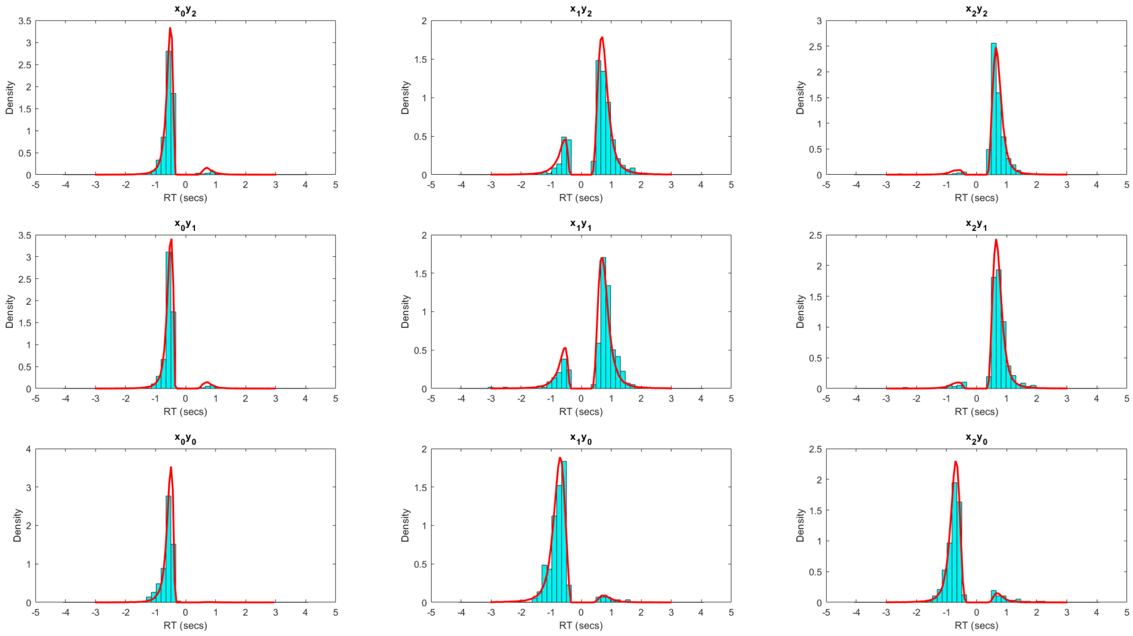

Posterior Parameter Distributions

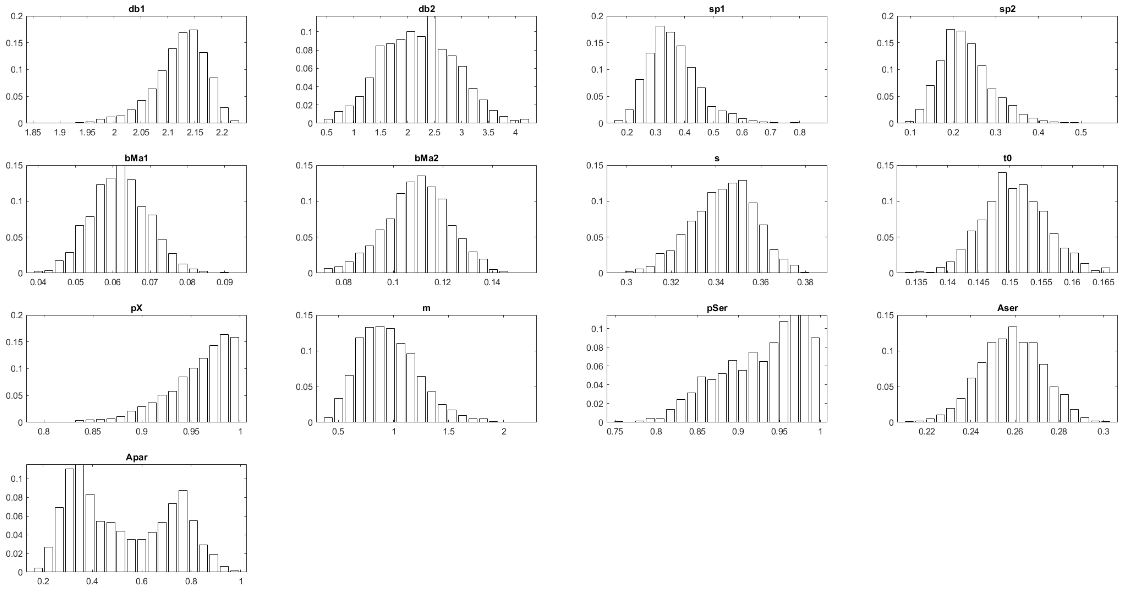

References

Cheng, X. J., & Little, D. R. (2025). Second-order facial features are processed analytically in composite faces.
(Manuscript in preparation)

Wagenmakers, E.-J., & Farrell, S. (2004). Aic model selection using akaike weights. *Psychonomic Bulletin & Review*, *11*, 192-196.
